# Vehicle-oriented and Sweden-framed life cycle assessment: Hydrogen for long-haul trucks

**DOI:** 10.1016/j.isci.2025.113607

**Published:** 2025-09-19

**Authors:** Jorge Enrique Velandia Vargas, Selma Brynolf, Maria Grahn, Felipe Rodriguez, David Blekhman

**Affiliations:** 1 Chalmers University of technology, Gothenburg, Sweden; 2 Colorado State University, Fort Collins, CO, USA; 3 California State University, Los Angeles, CA, USA

**Keywords:** Environmental science, Energy resources, Applied sciences

## Abstract

Hydrogen trucks are an alternative for decarbonizing the long-haul segment. However, the environmental footprint benefits depend on how hydrogen is produced, transported, and used but also in truck characteristics. We conduct life cycle assessment to quantify the impacts per ton-km. For centralized production, we included electrolysis and steam reforming cases, with dedicated transportation pathways but also included production onsite. We evaluated fuel cells and combustion engines and included supply chain hydrogen leakages. We found that global warming potential (GWP) of different truck versions varies up to 50tCO_2_eq per vehicle. Additionally, electrolysis powered by the Swedish grid appears more competitive than blue hydrogen, for most cases evaluated. For high hydrogen leakage scenarios (∼30%), GWP of green hydrogen, per ton-km, increases 2-fold. The low payload of tanker ships transporting hydrogen nullifies the benefits of importing green hydrogen. Truck manufacturing industries and low-carbon electricity enhance the potential for hydrogen to decarbonize the segment in Sweden.

## Highlights

• Life cycle assessment with a robust technical approach in Swedish conditions
• Hydrogen trucks offer decarbonization when compared to fossil counterparts
• Biofuels and electric trucks potentially offer more decarbonization than hydrogen
• Hydrogen leaks jeopardize the carbon mitigation obtained by renewable hydrogen

## Graphical abstract

**Figure:**
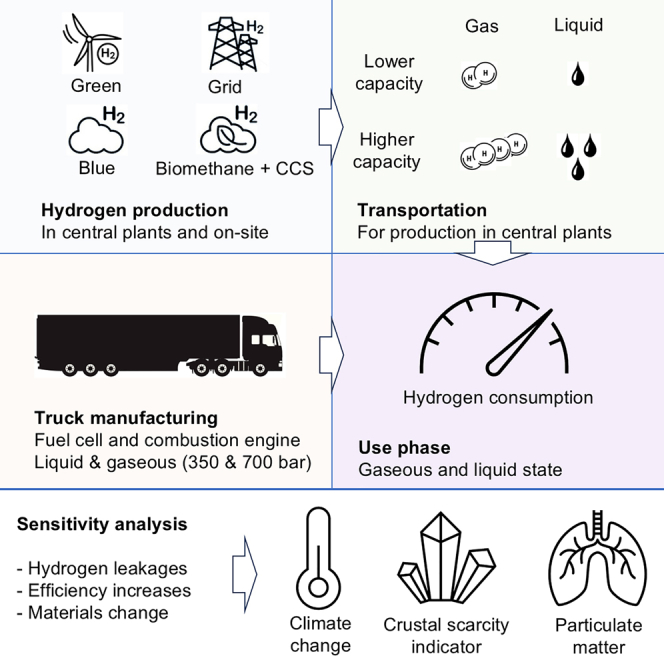

## Introduction

To keep global warming below 2°C, a substantial decarbonization of all economic sectors is necessary.^1^ Globally, transport is responsible for more than 50% of the oil demand,^2^ while the exclusive oil demand from road freight vehicles corresponds to one-fifth of global oil demand.^2^ Vehicles with a gross vehicle weight (GVW) greater than 15 tons are responsible for 65% of freight services, turning them into a key enabler of global economic activity.^2^

Road freight transport demand is expected to rise,^2^^,^^3^^,^^4^^,^^5^ increasingly putting more pressure on the sector’s oil demand. Although oil use from light-duty vehicles (LDVs) has been observed to plateau or decline,^2^ emissions from heavy-duty vehicles (HDVs), heavily reliant on diesel, have climbed to become the largest source of emissions in the European Union (EU).^6^ Decarbonization options for road freight transport include energy efficiency measures, use of biofuels and electrofuels (fuels produced from electricity, water, and carbon dioxide), and fleet electrification based on lithium-ion batteries (LiBs), as well as using fossil-free hydrogen in fuel cells (FCs)^3^^,^^5^^,^^7^ and in internal combustion engines.^8^

The potential of hydrogen for decarbonization of diverse economic sectors has been extensively included in decarbonization strategies around the world.^7^^,^^9^^,^^10^^,^^11^^,^^12^^,^^13^^,^^14^ The European Union (EU) hydrogen strategy^15^ leads to regulation 2021/1119, which established an EU target to reduce 55% of GHG emissions by 2030, compared to 1990 levels, aiming for full carbon neutrality by 2050.^5^^,^^16^ The hydrogen roadmap for Europe aims to deploy 45,000 HDVs, referring to buses and trucks, by 2030,^17^ which is nearly half the current market.^18^

Although FC technologies for LDVs have been, for years, in development by nearly every major automotive manufacturer, investments on the HDVs segment only started to gain momentum recently^19^; by the end of 2022, there were around 20 fuel cell truck (FCT) models available worldwide.^20^ A growing HDV fleet is expected in Europe as rapidly declining costs for FCs and batteries are expected to enable large-scale electrification of freight transport.^21^

Furthermore, automotive industry players and research institutes have launched pilot projects to explore the potential of hydrogen for internal combustion engine trucks (ICETs),^22^^,^^23^ which includes retrofitting of trucks to make them hydrogen compatible.^20^^,^^24^ Despite recent hydrogen efforts, batteries are the incumbent technology for decarbonization of road transport, and it has been estimated to be less emission-intensive than fossil-fuel-based alternatives in many regions.^25^

FCTs refuel faster than battery electric trucks (BETs),^16^ similarly to diesel trucks,^24^^,^^26^ and offer power/energy flexibility by adjusting FC stack capacity, hydrogen tanks size, and LiB capacity. Increasing energy or power attributes of BETs comes with a weight penalty,^19^ making them heavier than FCTs for similar payload and range.^27^^,^^28^^,^^29^ Shrinking BET battery capacity can match ICETs’ or BETs’ payload capacity but compromises range, which in turn could weaken the financial business case exacerbated by the hydrogen refueling station (HRS) scarcity. Additionally, fast charging imposes high loads in the grid,^30^ risking instability even at modest levels of electrification.^31^ Likewise, when demand exceeds capacity, costly upgrades are necessary, even for short-haul HDVs.^32^ Moreover, material scarcity risks have been reported for LiB supply chains.^33^^,^^34^^,^^35^^,^^36^^,^^37^

Nearly 60% of the global current hydrogen production in 2023—97 Mt per year—came from steam methane reforming (SMR) using natural gas (NG) as feedstock, while another 20% comes from coal.^38^ As the bulk of hydrogen production is currently linked to fossil-fuel feedstocks, adding carbon capture and storage (CCS) to the already mature technologies of SMR could allow the already available NG infrastructures to produce the so-called blue hydrogen (BH_2_). In fact, BH_2_ hydrogen is considered in European hydrogen policies for decarbonization.^39^^,^^40^ Nonetheless, warnings have been raised about its actual decarbonization potential.^41^^,^^42^^,^^43^^,^^44^^,^^45^^,^^46^ An alternative with higher decarbonization potential is electrolytically generated hydrogen from renewable electricity, also known as green hydrogen (GH_2_); however, it is estimated to be 2–3 times more costly than BH_2_.^47^

The adoption of hydrogen-based long-haul freight vehicles faces plenty of hurdles: lack of refueling infrastructure,^2^^,^^48^^,^^49^^,^^50^^,^^51^ high cost of hydrogen production and FCs,^19^^,^^47^^,^^52^^,^^53^^,^^54^^,^^55^ and a low well-to-wheel efficiency,^26^^,^^56^^,^^57^ which is greatly influenced by the energy consumption associated to compression and liquefaction processes associated to transport and storage of hydrogen.^56^^,^^58^^,^^59^^,^^60^^,^^61^^,^^62^^,^^63^^,^^64^ Moreover, concerns about the indirect GWP of hydrogen when released into the atmosphere place supply chain leakages in the spotlight.^65^^,^^66^

The purpose of this study is to estimate the environmental footprint of using hydrogen on FCTs and ICETs in Swedish conditions, through life cycle assessment (LCA). A literature review presented in Methods S1 (referring to section 1 of the supplementary methods) showed that only a few LCA studies on hydrogen vehicles focus on HDVs, mainly quantifying global warming. Moreover, our review found no studies that elaborated on the environmental footprint divergences caused by different approaches to truck design, for instance, variations linked to the physical state in which hydrogen is stored onboard: liquid (LH_2_) or compressed gaseous (CH_2_), and likewise, if CH_2_ is preferred, what differences are expected from choosing 350 bar or 700 bar tanks or from a larger FC or a larger LiB for dealing with peak power requirements. In addition, no study on hydrogen ICETs was found in our literature review, making it the first of its kind to the best of our knowledge. An in-depth modeling of eight truck configurations is included in Methods S2.1, while vehicle subsystems are presented in Methods S2.2.

**Figure 1:**
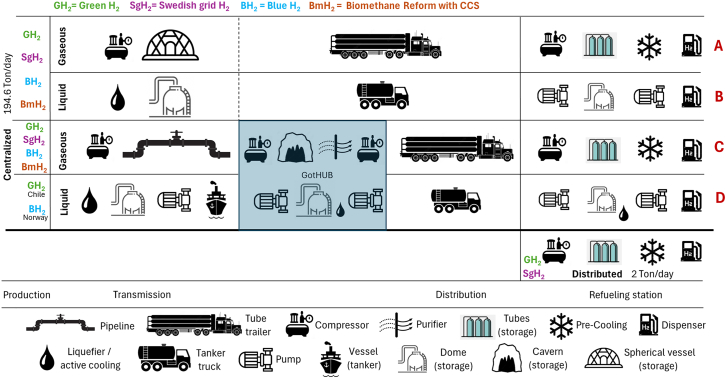
Hydrogen transportation pathways for centralized and distributed production cases Pathways A and B represent an early market, while B and D depict established supply chains.

In addition to GH_2_ and BH_2_, we also included electrolysis powered by the Swedish grid (SgH_2_) and biomethane reforming in combination with CCS (BmH_2_). Biomethane comes from upgraded biogas obtained from anaerobic digestion (AD) of biogenic waste. For GH_2_, we explored hydrogen production onsite, at the HRS, eliminating the need for transportation, but also at a central plant, enabling economies of scale and increasing electrolysis efficiency. See Methods S3.1 for GH_2_ and Methods S3.2 for BH_2_. For centralized production we proposed four transmission and distribution (T&D) pathways intended to represent early and mature supply chains using CH_2_ and LH_2_, as seen in Methods S4.1–S4.5. The developed market includes two cases of production abroad with subsequent import via tanker ship: Chile due to its low-cost electricity^9^ and Norway, displaying large NG reserves (Figure 1). Our literature review found no evidence of other studies performing in-depth technical LCA analyses of hydrogen supply chains in Sweden or northern Europe.

**Figure 2:**
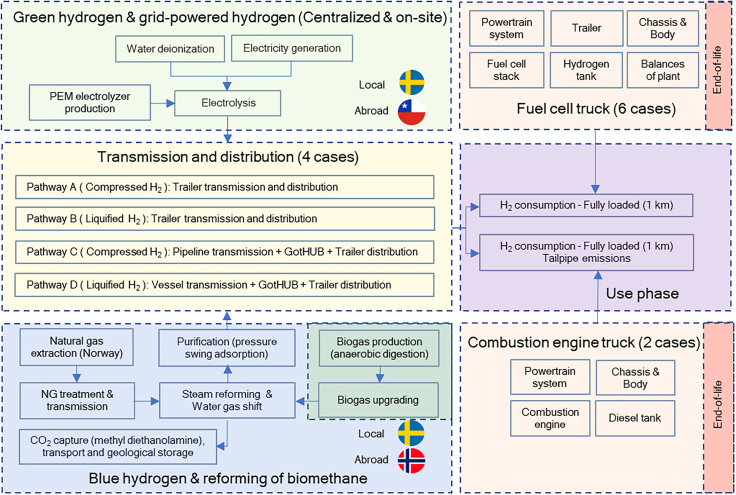
System boundaries This study includes hydrogen production in central plants and in distributed refueling stations. For centralized production, four transmission and distribution cases are evaluated.

For the use-phase, we estimated the hydrogen consumption per km for fully loaded FCTs and ICETs, based on literature review, own estimations, and exchanges with automotive industry experts. See Methods S5.1 for fuel consumption and Methods S5.3 for tailpipe emissions modeling. Finally, we performed a sensitivity analysis (SA) on salient parameters, including hydrogen leakages, efficiency increases, potential NG leakages for BH_2_ production, and two cases representing more disruptive technological changes: using recycled carbon fiber (CF) for hydrogen tanks and substituting conventional steel by steel based on direct reduction of iron (DRI) (Figure 4 and Methods S6.1–S6.3). The literature review found no LCA of hydrogen transport technologies including supply chain leakages or considerations of circular economy at the vehicle level. This study enriches the literature by providing a technically detailed LCA in Sweden, a key player in both long-haul truck manufacturing and in renewable generation. System boundaries are depicted in Figure 2.

We explored three mid-point impact categories: climate change (GWP-100 years) as parameterized by IPCC 6^th^ AR,^1^ crustal scarcity indicator (CSI),^67^ and particulate matter (PM-EF)^68^ as found in Environmental Footprint 3.0.^69^

## Results and discussion

### Overall results

**Figure 3:**
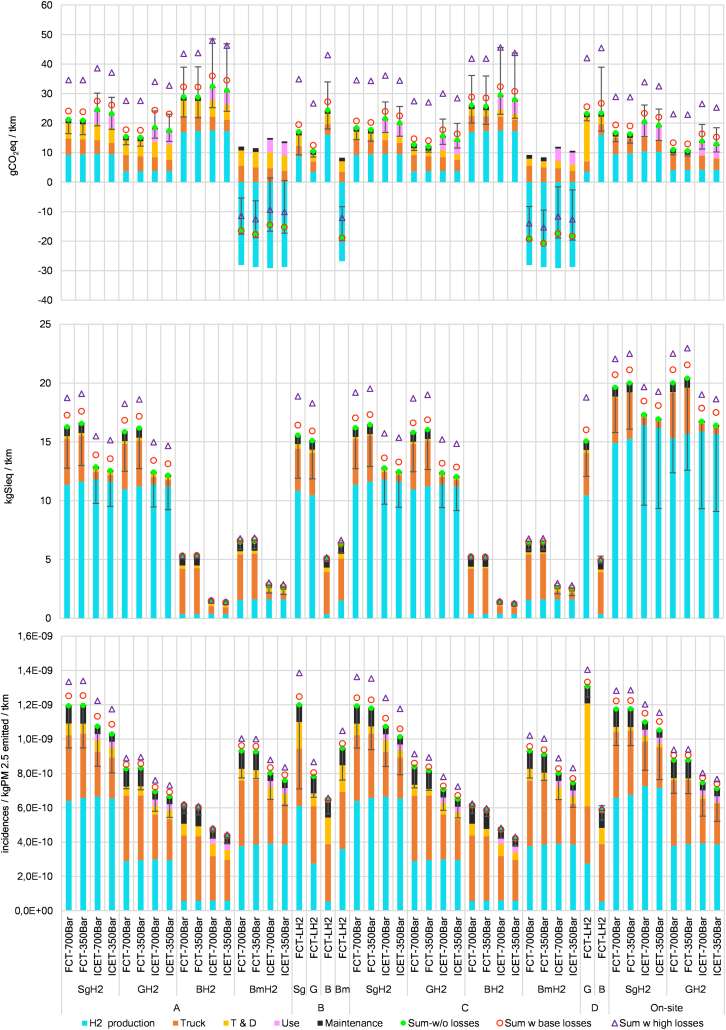
Climate change (GWP), crustal scarcity indicator (CSI), and environmental footprint-particulate matter (PM-EF) results for the chosen transportation pathways and truck configurations that incorporate fully loaded 42-ton trucks (GVW) All presented FCTs correspond to a 200-kW fuel cell—140 kWh vehicles. Bars represent the cases included in the sensitivity analysis (Figure 4). Hydrogen leakages for low and high estimations are represented by red circles and purple triangles, respectively. For all impact categories the effect of leakages refers to the extra production required to make up for the losses throughout the life cycle. In addition, for climate change, hydrogen leakages also include the low and high GWP effect estimations of releasing hydrogen into the atmosphere. Leakages are estimated with respect to the sum of impacts without any leakages (green circle). Low and high GWP_100_ factors for hydrogen are 11.6 ± 2.8 kgCO_2_eq kgH_2_^−1^. For on-site production, the small contribution of T&D refers to compression at the hydrogen refueling station. Key differences between the transport cases are the following: (A) trailer gas 1,000 kg (500 bar) 150 km; (B) trailer liquid 4,000 kg (−253°C) 150 km; (C) pipeline gas (70 bar) 150 km; and (D) vessel run on biodiesel (−253°C) 14,600 km.

Figure 3 presents GWP results, per ton-km (tkm), for all pathways. In BH_2_ and SgH_2_ cases, hydrogen production is the dominating contributor. BmH_2_ yields net negative GWP prompted by carbon fixing during biomass growth, the subsequent geological storage, and soil application of digestate.^70^ For GH_2_, the reduced footprint of production makes truck manufacturing the largest contributor while T&D becomes increasingly relevant for A and B pathways. For distributed production, only slight GWP differences per tkm are observed, compared to central production cases, despite the lower efficiency of smaller on-site electrolyzers (55.5 vs. 60 kWh kg^−1^) (see Methods S3.1.1).

**Figure 4:**
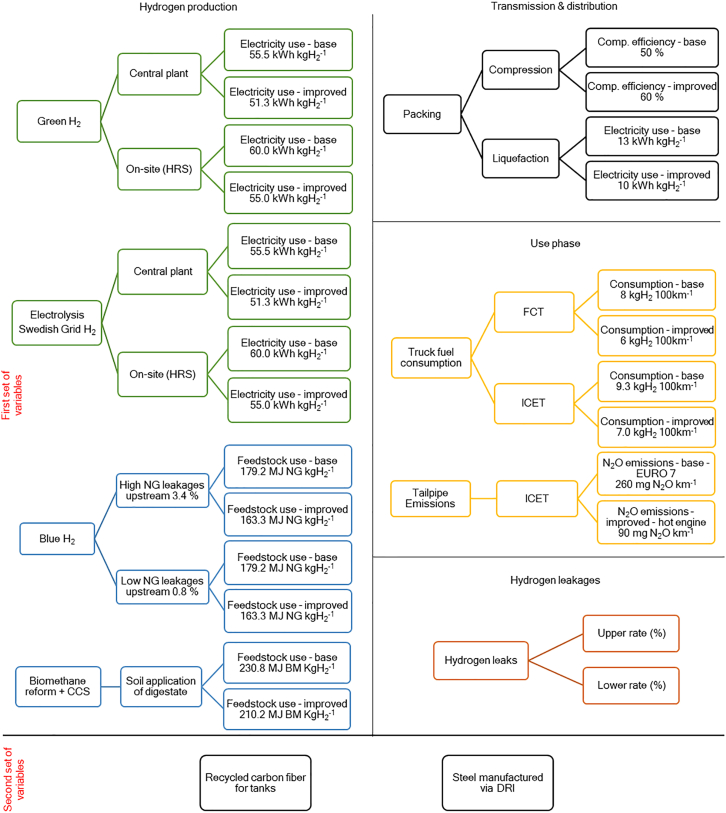
Variables included in the sensitivity analysis The first set of variables reflect technological changes likely to happen in the near future. In contrast, NG and hydrogen leaks reflect undesired characteristics of the proposed supply chains. Instead, the second set of variables represents more drastic changes in current technologies. Sensitivity analysis assumptions are presented in Methods S6.

The SA results are incorporated in Figure 3 and represent the first set of variables exhibited in Figure 4. Most variables in the SA reflect anticipated efficiency improvements, making the lower end of the range larger than the upper part, for all impact categories, except for climate change of BH_2_ and BmH_2_ cases. Counterintuitively, fuel consumption improvements in BmH_2_ cases result in GWP increase as the carbon removal from the atmosphere depends on the BmH_2_ produced; therefore, using less BmH_2_ translates into less carbon removal. The GWP of ICETs is higher than that of FCTs for same storage pressures, mainly due to N_2_O tailpipe emissions from ICETs. Such emissions are legally limited by the EURO 7 standard to 260 mg N_2_O kWh^−1^.^71^ However, in hot engine conditions, emissions reach 90 mg N_2_O kWh^−1^,^37^ or even lower.^72^^,^^73^ The GWP of the use-phase is driven by N_2_O emissions, with AdBlue and pilot fuel (biodiesel), displaying small contributions (see Methods S5.2).

Despite the low methane leakages, the GWP of BH_2_ from Norwegian NG is larger than that of on-site SgH_2_. Concerns about BH_2_ have been documented regarding emissions and energy consumption,^44^^,^^70^^,^^74^ unfulfilled expectations,^41^^,^^45^^,^^75^ prioritization of public funds for other areas,^46^^,^^75^^,^^76^ the intensive use of water during CCS,^77^ burden shifting from GWP to other impact categories,^70^ the few operational facilities worldwide,^78^ and the NG availability in Europe driven by the war in Ukraine.^79^^,^^80^ BH_2_ pathways display lower CSI impacts since the method does not have a characterization factor for lithospheric NG.^67^ CSI assesses elements in the Earth’s crust, but NG is not recognized as a component of lithosphere,^81^ rather a substance trapped in it. Besides, steam reformers do not use rare earths of other high CSI impact materials. Furthermore, the particulate emissions during BH_2_ production^70^ result in the lowest PM-EF impacts while combustion of biomass is the largest source or PM for electrolysis pathways amplified by the large electricity consumption.

Biomethane can replace NG with minimal changes to reforming facilities.^70^ However, net carbon removal stems not only from CCS but also from soil application of digestate and from avoiding land use change.^70^ Additionally, carbon balances depend on system boundaries and assumptions.^70^ Moreover, the availability of biomethane for steam reforming is far from guaranteed. Sweden, a leading European biogas producer,^82^generated ∼2.3 TWh in 2022 (67% of which was upgraded to biomethane),^83^ with studies revealing potential for 7–10 TWh in a few years^84^ by boosting food residue sorting and centralized organic waste processing.^82^^,^^85^ Still, biomethane supply is unlikely to be scalable in the magnitude required to power the EU’s HDV fleet. For instance, the total technical potential of biomethane in the EU-27 could replace only 8% of total NG consumption in 2030.^86^ Regarding economic potential, the percentage decreases to 2% in 2030 and increasing to 6% in 2050.^86^

GH_2_ pathways show the lowest GWP, except when imported from Chile. Conversely, GH_2_ and SgH_2_ present the highest CSI impacts driven mainly by iridium, and to a lesser extent by platinum, incorporated in PEM electrolyzers. Iridium is the raw material with the highest characterization factor in the CSI method.^67^ Switching from PEM to alkaline electrolysis (AE), a technology which is established, durable, cheaper, and free of precious metals,^87^ would drastically diminish CSI impacts. However, PEM was chosen for its fast response to the intermittency of renewables and its high-purity yield, crucial for FCs and ideal for storage and liquefaction. For climate change, PEM and AE have similar impacts per kg of hydrogen^88^^,^^89^ but having a production plant based on AE would either hinder the use of intermittent renewable sources or would require larger hydrogen storage facilities (see Methods S3.1 and S4.3) or the storing of such renewable electricity. Furthermore, in SgH_2_ cases, PM-EF impacts arise, compared to GH_2_, mainly from the upstream particulate emissions in biomass cogeneration and nuclear power, amplified by the intensive electricity use during electrolysis.

For CSI and PM-EF, the impact of hydrogen leakages exclusively refers to the extra hydrogen needed to replace losses. In electrolytic cases, leak-driven CSI impacts are substantial due to the PEM stack containing precious metals. For climate change, we added the GWP of leaked hydrogen (11.6 ± 2.8)^66^ (see Methods S6.1). Compared to no-leak scenarios, low-leak GWPs can rise by up to one-third, with the highest increases for GH_2_ cases, suggesting that leakages jeopardize the benefits of renewable hydrogen. Under high-leak scenarios, GWP can increase up to two-and-a-half times for GH_2_ in pathways B and D, highlighting the leakage risks for LH_2_.^64^^,^^90^^,^^91^^,^^92^ Once again, we observe opposing GWP effects when BmH_2_ is leaked. As each kilogram of hydrogen removes atmospheric carbon, replacing leaked BmH_2_ enhances net carbon removal.

### The vehicles

**Figure 5:**
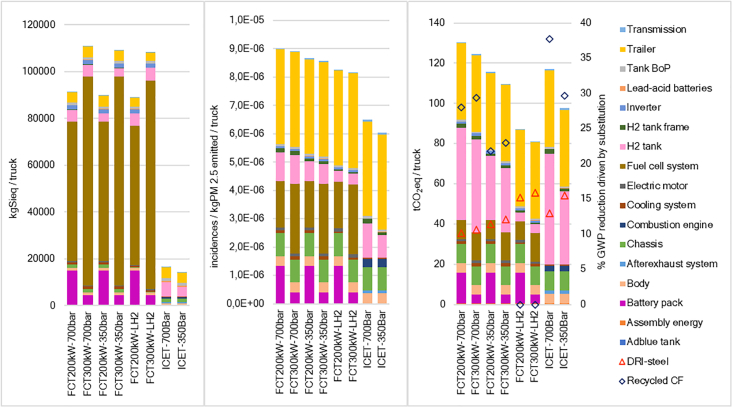
Truck manufacturing impacts (GWP_100_) for the CSI, PM-EF, and GWP_100_ FCT-200 refers to trucks with 200 kW fuel cells and 140 kWh LiB, while FCT-300 contains a 300 kW fuel cell and a 40 kWh battery. The right axis in the GWP graphic presents the GWP reduction caused by substituting conventional steel by DRI steel in selected components and by replacing virgin carbon fiber by carbon fiber recycled via pyrolysis.

The contribution of truck manufacturing to GWP per tkm has been estimated to be secondary to that of hydrogen production^16^^,^^57^^,^^93^^,^^94^ since hydrogen has mostly been produced from fossil feedstocks and the truck footprint per tkm is diluted over the entire vehicle lifetime (around 1,000,000 km). Still, truck manufacturing relevance per tkm is expected to increase as hydrogen goes low carbon. Our GWP estimations for the manufacturing of the eight evaluated configurations range from 130 tCO_2_eq to 81 tCO_2_eq per vehicle. For details see Methods S2.1 and S2.2. Truck footprint relevance is notorious when considering fleet sizes: during the first quarter of 2024 more than 67,000 HDVs >12 t were sold in the EU, nearly 50% of them truck trailers.^95^ Figure 5 shows the estimated GWP per truck, and the secondary axis shows the GHG mitigation (%) driven by the second set of variables in the SA: the substitution of virgin CF by recycled CF in the hydrogen tanks and the partial substitution of virgin steel by DRI-based steel.

Three hundred fifty bar CH_2_ trucks have lower GWP than their 700 bar counterparts due to reduced CF use in their tanks. For 350 and 700 bar tank dimensioning, see Methods S2.2.3. ICET configurations exhibit the highest GWP since they require an extra tank, compared to their FCT counterparts, due to lower powertrain efficiency (Methods S5.1) and aiming to match the range of 1,000 km offered by diesel trucks, a strategy that alleviates range concerns due to HRS scarcity while keeping short refueling times. Payloads remain on par with analogous commercial diesel trucks^29^^,^^57^ and comply with EU regulations permitting a 2 t GVW increase for clean fuel vehicles.^96^^,^^97^

Besides the tanks, the vehicle subsystems contributing the most to GWP are LiBs, FCs, and trailers. FCT_300_ versions outperform FCT_200_ by about 6 tCO_2_eq per truck by prioritizing larger FCs over bigger batteries. However, it is unclear what approach truck manufacturers will prefer to face peak power demands: larger batteries or larger FCs or even if different truck versions will be commercialized. Large GWP from LiB is mostly traced to Chinese battery cells whose manufacture is powered by a carbon-intensive grid.^98^ Shifting production to greener grids is expected to reduce GWP; however, smaller facilities may raise per unit footprints.^99^

FC GWP is dominated by the platinum loading in the stacks. Although platinum use represents both an economic and mineral depletion hotspot, it is unclear whether material reduction will be the focus of research in the future. Instead, it is suggested that future research may shift from low-platinum cells toward durability and integration solutions for longer HDV lifetimes.^19^ Moreover, selective catalytic reduction (SCR) for hydrogen ICETs was assumed to use TiO_2_ while no oxidation catalyst is required, sparing the use of platinum. For CSI, platinum-based FCs are the dominant contributor over LiBs (NMC 611), as cobalt’s characterization factor in CSI is lower than that of platinum.

LH_2_ trucks have the lowest GWP since CF is absent from their steel tanks.^100^ Although the high energy demand from liquefaction raises upstream impacts, it is partly offset by the high share of renewables in the Swedish grid. Cryo-compressed hydrogen (CCH_2_) combines attributes from CH_2_ and LH_2_, reducing carbon fiber needs and boil-off.^100^ CCH_2_ trucks are beyond the scope of this study but its GWP is expected to range between that of CH_2_ and LH_2_.

Despite the considerable environmental footprint of manufacturing each truck, hydrogen production is observed to be the main GWP contributor per tkm (see Figure 3). This is particularly relevant as the massive ramp-up in GH_2_ production forecast for the next decades seems to teeter under the weight of its own optimistic goals,^101^^,^^102^^,^^103^^,^^104^ and Europe is unlikely to meet its 2030 targets.^105^ To turn the situation around, cost reductions in electrolyzers and renewable electricity^9^^,^^47^ are indispensable.

Additionally, LiBs evolve rapidly, suggesting that BETs could be viable for long-haul. Studies argue that reduced ranges are not necessarily a dead-end for BETs,^106^ emphasizing that frequent stops for recharging, due to shorter range, match the compulsory driver stopovers in EU legislation.^107^ Others go further and argue that the techno-economic developments in battery technology threaten the window opportunity for FCTs to establish a relevant market share, despite the high energy demand per km of BETs.^54^ The weight penalty BETs formerly suffered, compared to FCTs, has been forecast to diminish to only 0%–10% in 2030, for ranges of up to 1,000 km,^29^^,^^108^ suggesting a payload penalty reduction, decreasing the environmental footprint per tkm for BETs. Furthermore, in the UK, only 10%–19.5% of HDV trips are weight constrained,^109^ while in Germany, around 30% of HDV trips are driven empty,^110^ indicating that BET footprint per tkm would only be penalized at high load factors.

Moreover, BET fleets require a charging network operating at high power.^30^^,^^54^ The distance between such points has been estimated to be as short as 50 km^111^ although AFIR declares it at 60 km.^112^ Fast charging infrastructure could cause grid instability even at modest levels of electrification,^31^ and costly updates can be required when demand exceeds the system capacity even for short-haul HDVs.^32^ Conversely, studies have estimated that high-power charging points (>1 MW) would only represent a small share of the total charging infrastructure.^113^

### Transportation pathways

Regarding hydrogen transported from central plants, pathways A and B, which represent early markets with low trading volumes, exhibit the highest GWP. In pathway A, tube-trailer transport (1 t H_2_ payload) is the dominant contributor. In pathway B, liquefaction powered by the Swedish grid is the main contributor; when liquefaction is powered by wind, as in GH_2_ cases, tank-trailer transport (4 t H_2_ payload) overtakes liquefaction as the largest burden. Pathway C, picturing a market with trading volumes large enough to justify the construction of pipelines and the GotHUB, yields the lowest GWP, with tube-trailers used for distribution contributing the most.

Pathway D covers imports via tanker ship from Chile (GH_2_) and Norway (BH_2_). GWP impacts from the Chilean case are vastly dominated by transmission and are associated to the low payload capacity of the LH_2_ tanker (9,800 t), resulting in the highest GWP, negating GH_2_ emissions mitigation, and resulting in impacts similar to those of SgH_2_, in pathway A or B. Impacts of transmission from Norway are tempered by shorter transmission distance. CSI impacts of T&D per tkm are negligible for all cases; PM-EF impacts primarily arise from tanker-ship transport. Nonetheless, geographic differentiation of intake factors complicates PM-EF evaluation^68^ when emissions happen in such dissimilar conditions. Thus, PM-EF results are intrinsically limited by the uncertainties inherent to emissions released in different locations with different degrees of exposition to humans.

Hydrogen purity was not identified as a concern in pathways A, B, or D since all storage and T&D vessels are hermetic, while all produced hydrogen is FC-grade, either electrolytic^114^ or SMR+PSA.^70^ For LH_2_, high purity is necessary for storage and liquefaction.^64^ Pipelines and LRC in pathway C risk hydrogen contamination,^64^ so purification at GotHUB is included before distribution to the HRS (see Figure 1). ICETs do not necessitate FC-grade hydrogen, eliminating purification needs. Nonetheless, sharing T&D infrastructures for FCT- and ICET-intended hydrogen is challenging as it risks polluting the FC-grade hydrogen required for FCTs but also the equipment. Moreover, small-scale SMR of biomethane can supply hydrogen pure enough for ICETs without further purification but the presence of pollutants demands attention to hydrogen embrittlement^115^ for future storage or transportation in pressurized vessels. Moreover, pollutants in hydrogen are incompatible with liquefaction.

Moreover, our results suggest which infrastructures are more effective for GWP mitigation. For instance, pathway B consistently appears to have one of the lowest GWP for all production technologies, which support the adoption of large-scale infrastructures for LH_2_ storage at production plants, but concerns about hydrogen leakages must be addressed. In contrast, the deployment of large LH_2_ infrastructures at the GotHUB does not automatically translate into the lowest GWP, as early tanker ships are heavily penalized by their low payload capacity. This renders pathway D very sensitive to transportation distance. Indeed, Norwegian case displays the lowest GWP of all BH_2_ while Chilean hydrogen shows the largest GWP among GH_2_ cases. Whether transoceanic transport of LH_2_ will represent a significant share of the market or it will mostly happen in the form of liquid organic hydrogen carriers will likely depend on future payload capacities.

In addition, the large investments in pipelines within pathway C do not necessarily translate into the lowest GWP due to the high impact of the distribution leg, via tube-trailer. In fact, pathway A, based on 1t tube trailers consistently presents the highest GWP impacts. Larger capacities are achievable if carbon fiber tanks replace the conventional metallic tubes but a much larger GWP from the tube trailer is expected and a trade-off will take place. Investigating such trade-offs is beyond the scope of this study. In any case, storage capacity will be necessary at the HRS. We believe this storage will take place in a gaseous state as the boil-off percentage increases in smaller LH_2_ tanks, compared to larger units (see Methods S4.3). A public policy focused on measuring and mitigating hydrogen leakages is beneficial for every pathway, even for onsite production.

### GWP comparison with other truck alternatives

**Figure 6:**
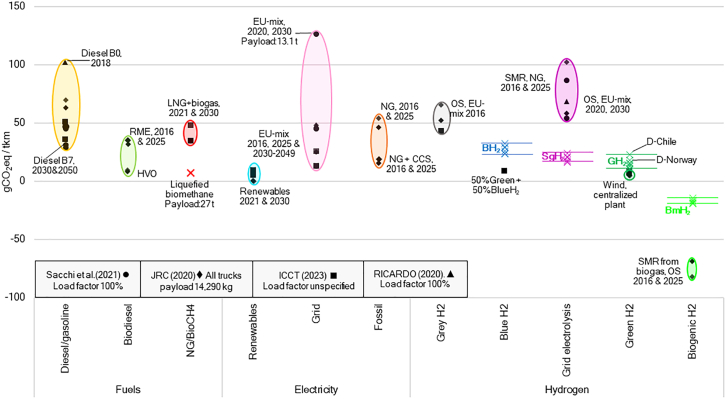
GWP results per tkm compared to estimations in the literature RME, rapeseed methyl ester; HVO, hydrotreated vegetable oil; OS, onsite. In Sacchi et al.,^57^ vehicle range is 800 km while lifetime is 1,050,000 km. In ICCT,^16^ vehicle range is 500 km for both FCTs and BETs while life expectancy is 1,243,000 km. In JRC,^37^ FCT range is 614 km in 2016 and 746 km in 2025, BET range is 372 km in 2016 and 376 km, and ICET range is 3,400 km while life expectancy was undisclosed. In Ricardo,^93^ range is 500 km and lifetime is 800,000 km. More transparent shapes represent future estimations for the same study, while X marks the results of this study. The case where liquefied biomethane is directly used in ICETs is indicated with a red X (see Methods S7).

Figure 6 compares our GWP results with the literature.^16^^,^^57^^,^^93^^,^^94^ For the SgH_2_ cases, electricity mix we considered (41.2 gCO_2_eq kWh^−1^)^116^ resulted in a total GWP per tkm that is similar to that of BETs powered by the EU-grid for 2021–2040 (197 gCO_2_eq kWh^−1^) and 2030–2049 (129 gCO_2_eq kWh^−1^)^16^ despite the longer range and shorter lifetime of our trucks, the much lower well-to-wheel efficiency of hydrogen, and the fact that the BETs do not represent a fully loaded truck.^16^ Moreover, SgH_2_ performs better than any FCT using EU-grid-powered hydrogen while rivaling 2050 ICET projections for diesel ICETs,^57^ despite our trucks having longer range, shorter lifetimes, and lower payload capacity (29.7 t vs. ∼24 t). It is important to understand that our methodological choices impose limitations on the conclusions we can draw from the SgH_2_ results. Due to the attributional nature of this study, we use an average Swedish electricity mix.^116^ However, this is not ideal when the countries form a common electricity market^117^ like the one found in Scandinavia. Furthermore, the carbon intensity of the Swedish grid varies, depending on the electricity price zone and the hourly profile.^118^

GH_2_ cases appear competitive with HVO-ICETs^94^ and renewable-hydrogen-powered BETs,^16^ the lowest GWP alternatives in this comparison. Even the highest GWP, found in Chilean-GH_2_, performs better than any fossil ICET. In contrast, BH_2_ cases present comparable GWP to 2050 estimations for fossil ICETs,^16^^,^^57^ 2030 projections of LNG+biogas,^16^ and 2030–2049 projections of EU-grid BETs.^16^ BmH_2_ net carbon removal is lower than other SMR biomethane estimates.^94^ A case where liquefied biomethane is directly used in ICETs is included, see Methods S7.

From all production technologies, BH_2_ displays the highest GWP followed by SgH_2_ and GH_2_. Variations caused by truck version and T&D pathway are visible. The SA warns against the GWP of potential NG leakages for BH_2_ and N_2_O tailpipe emissions for ICETs. Using recycled CF for hydrogen tanks and replacing conventional steel by DRI steel may reduce GWP by about 30% and 15% per truck, suggesting potential synergies between truck manufacturers and two particular cases of decarbonization, one based on circular economy approach and the other based on decarbonization of steel, both of which are of high interest to Sweden. Hydrogen leaks can increase GWP with the steepest increases observed in GH_2_ and LH_2_ cases, which present the highest percentual rises, thus jeopardizing the carbon mitigation gains obtained by investing in renewable generation.

Moreover, although flexible, CH_2_ distribution based on tube trailers results in the highest GWP due to the low capacity of tube trailers. In contrast, LH_2_ transport by tank trailers presents the lowest GWP, but this benefit will not extend to pathways including tanker ships, unless the payload capacity of the vessels increases. Indeed, when imported from Chile, GH_2_ has comparable GWP as the SgH_2_ produced at the HRS despite the lower efficiency. ICETs exhibit higher GWP compared to FCTs linked to the heavier tank system, included to equalize ICET and FCT range, but also to small N_2_O emissions. Regarding CSI, electrolysis-based pathways present the largest impacts due to iridium and platinum in PEM electrolyzers while SMR cases require no rare earths. For PM-EF, the largest impacts are traceable to the electricity used for electrolysis or to the transport via tanker ship.

### Limitations of the study

The technical choices in this study represent Swedish conditions and imply multiple assumptions over entire supply chains that still do not exist and integrate rapidly changing technologies for which up-to-date data are difficult to obtain. Our approach aimed at providing an in-depth technical analysis of key technologies. Nonetheless, technological breakthroughs could transform the depicted processes beyond our estimations in the SA. Obtaining first-hand data was unfeasible. For the trucks, it is unclear which vehicle versions will be released commercially, especially regarding the hydrogen storage, FC, and battery capacities for FCTs and the engine and exhaust aftertreatment specifics for ICETs. Likewise, T&D modeling is heavily influenced by storage times, transportation distances, and leakage estimations, especially for LH_2_ pathways.

Moreover, the life cycle modeling integrated different processes from different sources. This results in system boundaries that sometimes overlap, potentially inducing double counting of inputs, while in other occasions gaps might appear. In both cases, we devoted efforts to correct the problem and to present coherence with an attributional LCA approach; for instance, in BH_2_ LCIs we removed the electricity for compression till 200 bar as it is included in our T&D stage (see Methods S3.2). Furthermore, using average data to describe the Swedish electricity mix, a common practice in attributional LCA, is not ideal to describe a national grid part of a common market. Thus, we are unable to capture carbon-intensity variations caused by marginal dispatch of fossil-based generation or surge in imports from other countries during demand peaks. Fortunately, this impact would only be relevant in SgH_2_ cases. Furthermore, the carbon balance made for original BmH_2_ LCIs are not adequate for including impact categories other than climate change. However, we do not expect any representative impacts in CSI or EF-PM from biomethane production.

Furthermore, this study is unable to quantify the depletion of NG used for BH_2_ production as CSI only considers minerals present in the lithosphere and considers NG as a substance trapped in the ground rather than a part of earth’s crust. Moreover, the geographic dispersion of the particulate emissions over the entire supply chains and the large uncertainties regarding the exposure of humans to such emissions translates into larger uncertainties in PM-EF evaluation in cases where hydrogen is imported.

## Resource availability

### Lead contact

Requests for further information and resources should be directed to and will be fulfilled by the lead contact, Jorge Enrique Velandia Vargas (jevelandiav@unal.edu.co).

### Materials availability

This study did not generate new unique reagents.

### Data and code availability

• All data reported in this paper will be shared by the lead contact upon request.
• This article does not report original code.
• Any additional information required to reanalyze the data reported in this paper is available from the lead contact upon request.

## STAR★Methods

### Key resources table

**Table:** 

REAGENT or RESOURCE	SOURCE	IDENTIFIER
**Deposited data**		

Ecoinvent 3.8 (database)	Ecoinvent	https://support.ecoinvent.org/ecoinvent-version-3.8
GREET	Argonne National Laboratory	https://greet.anl.gov/greet/versions.html

**Software and algorithms**		

OpenLCA	GreenDelta	https://www.openlca.org/

### Method details

#### Scope, system boundaries and impact assessment

This LCA study aims to estimate the environmental footprint of using hydrogen in long-haul FCTs and ICETs in Sweden. As it is usual for long-haul trucks to cross borders with other countries on the EU market, we assume the trucks must comply with EU regulations in terms of maximum GVW and dimensions.

As the data obtained from the literature review is bound to large uncertainties due to the rapid evolution of trucks and hydrogen technologies for production transportation and storage, we defined a time scope that comprises the decade between 2020 and 2030. Proposing a narrower time scope would be inaccurate as the hydrogen supply chains modeled here are still non-existent, implying multiple assumptions. Thus, we do not claim to be able to determine the specific evolution of technologies in the defined time scope, rather, we explore what the technology would look like during this decade. For better dealing with uncertainties, we performed an SA for parameters deemed as relevant from an environmental footprint viewpoint.

We employed an attributional approach. Foreground data collection was based on peer-reviewed studies and discussions with experts in the truck industry with experience in hydrogen applications.^119^^,^^120^^,^^121^ For background data we appealed to Ecoinvent V3.8 cut-off datasets, including the allocation criteria for multifunctional processes. The functional unit of the study was set to “the service of transporting one ton in a fully loaded 42 t GVW truck over one kilometer”. Looking for a fair comparison between different truck configurations, all hydrogen storage systems are designed to provide roughly 1,000 km range while the propulsion system provides 350 kW, and the trailer was assumed to be identical for all truck versions.

The main intended audience of this study includes OEMs from the automotive sector, truck fleet operators, policy and decision makers. We considered three mid-point impact categories: global warming potential (GWP 100years) as parameterized by IPCC 6^th^ AR,^1^ crustal scarcity indicator (CSI)^67^ and particulate matter (PM-EF)^68^ also known as respiratory inorganics as found in Environmental Footprint 3.0.^69^

#### Hydrogen production

Despite not being a formal classification, hydrogen production is colloquially categorized by colors. This study includes the production of green hydrogen (GH_2_), which refers to electrolytic production powered 100% by dedicated wind farms. We explore the GH_2_ production in Sweden, but also a case of production in Chile, a country estimated to have the lowest levelized cost of hydrogen,^122^ hence, we evaluate if the potential for low-cost production translates into low environmental footprint.

Hydrogen produced via SMR of NG is labeled as gray hydrogen. When this process is followed by a CCS stage, aimed at avoiding the release of fossil carbon to the atmosphere, it is informally called blue hydrogen (BH_2_). We explore one case where the NG is imported from Norway and BH_2_ production happens in Sweden, and the opposite case, where Norwegian BH_2_ is transported to Gothenburg via ship. Norway is a natural choice for NG provider due to its abundance, considering that competitiveness of fossil fuel-based hydrogen hinges on the availability of relatively low-cost gas resources.^123^ Furthermore, we explore one case of SMR including CCS but using biomethane as feedstock instead. This has the potential to produce hydrogen while also removing CO_2_ from the atmosphere.^70^ FC grade hydrogen (99.97% purity) is produced in all production pathways.

Beyond the environmental considerations, choosing a centralized or distributed approach depends on the trade-off between the cost of producing hydrogen, at a given location, and the cost of transporting it to the HRS. For GH_2_ the cost will depend on the available electricity sources and electrolyzer capacity factor^49^ whereas for BH_2_, the costs will depend on the access to existing gas grids or carbon storage locations.^124^ In any case, this discussion needs considering the transport distance, the amount to be conveyed, the physical state: CH_2_ or LH_2_, as well as the means of transport.^49^

#### Green hydrogen and Swedish grid hydrogen

The environmental footprint of electrolysis has been vastly studied.^125^^,^^126^^,^^127^^,^^128^ These studies have estimated that the use of precious metals is significant for metal scarcity impact categories while the carbon footprint of hydrogen is dominated by the carbon intensity of the process electricity. In fact, Lotrič et al.^129^ suggest that the operation phase contributes the most to the carbon footprint while the contribution of manufacturing is comparatively small.

Different production scales also affect the environmental impacts. Hydrogen can be produced both on large, centralized, production plants, which is advantageous due to economies of scale. Consequently, hydrogen transport from the central plant to the HRS is then required; an energy and cost intensive process.^49^^,^^56^^,^^60^^,^^61^^,^^62^^,^^63^^,^^64^^,^^130^^,^^131^ In contrast, hydrogen can be produced in each HRS, also known as distributed, or on-site, production, which eliminates the necessity of distribution to end users. However, this relinquishes the advantages of mass scale production. We included both cases to explore the environmental footprint tradeoffs linked to the choice of centralized and distributed production, powered by wind farms and the Swedish grid. The LCIs for the electrolyzer were obtained from Bekel & Pauliuk,^114^ which are based on PEM technology.

Additionally, as the source of electricity is crucial for the environmental footprint estimation, we explore two additional variants for each, centralized and distributed facilities, but this time having the electrolysis powered by the Swedish electricity grid (SgH_2_). For the Swedish mix we included the Ecoinvent dataset “*electricity, high voltage | electricity, high voltage, market for*| *cutoff”* which pictures a mix dominated by low carbon sources; small hydro 30.3%, large hydro 7.6%, nuclear 39.6% and wind 10.1%, with around 0.9% from fossil sources, among them cogeneration based on natural gas, anthracite and fuel-oil. 2.1% of the electricity is imported from Denmark while 5.4 comes from Norway. The total GWP_100_ of the Swedish grid is estimated as 41.2 gCO_2_eq kWh^−1^. This value is in consonance with other estimates.^132^

The intermittent nature of wind generation – and its lower capacity factor– means that, to guarantee a constant hydrogen supply, hydrogen storage is required.^50^ Swedish researchers have estimated the storage capacity for a prospective production plant to be 15% (20% on the conservative side) of the total production,^50^^,^^133^ although this storage could jeopardize the financial feasibility of the project.^134^ For simplicity, we assumed the SgH_2_ cases will not require storage facilities considering the flexibility granted by the network. These cases do not strictly classify as GH_2_.

There is great uncertainty on what would be the total demand for hydrogen in Sweden.^50^ Likewise, future hydrogen demand for hydrogen trucks is also uncertain. Founded on discussions with truck manufacturers we assumed the first generation of HRSs would need to supply 2 tons of hydrogen per day, enough for 25 FCTs storing CH_2_ at 700 bar. Moreover, for centralized plants we assumed the equivalent of a 500 MW electrolysis plant would satisfy the first stages of a hydrogen economy. Methods S3.1.1 exhibits the assumptions (efficiency, capacity factor, electricity inputs, etc.) for each of the four evaluated cases. The electrolyzers’ performance and dimensions incorporated in this study are in consonance with the Stage 2 of electrolyzers’ deployment forecast by IRENA,^47^ whose underlying assumption is that module-sized PEM electrolyzers range from 20 MW to 100 MW while large plants range from 0.1 to 5 GW.

#### Blue hydrogen and biomethane-based hydrogen

Despite only representing 0.6% of global hydrogen production in 2022, its potential for GHG emissions mitigation has inspired research on the current and future state of the technology^135^^,^^136^ and its techno-economic performance.^53^^,^^137^^,^^138^^,^^139^ Likewise, the environmental footprint has been estimated, from a life cycle perspective,^44^^,^^70^^,^^74^ whereas mixed LCA & Economic approaches are also found.^140^ We found consensus in pointing at three main contributors for environmental footprint: the methane emissions from NG supply chains, the production technologies, which define the carbon capture rates, and the choice of metrics for quantifying the GWP impacts.

Although the GHG effect of methane emissions has been recognized,^141^^,^^142^ estimating the specific GWP requires a specific time frame. The estimated GWP of methane varies over time, since the half-life of methane in the atmosphere is approximately 12 years, far less than that of CO_2_.^1^ Therefore, methane GWP is estimated to be around 30 and 85 times higher than that of CO_2_ over 100 and 20 years, respectively.^1^^,^^44^^,^^74^ In fact, considering a 3.5% methane leakage rate from NG and estimating GWP factors for 20 years, the total CO_2_ equivalent emissions for BH_2_ are only 9–12% lower than those of gray hydrogen.^44^

Research has indicated that the potential of BH_2_ in scaling up low-carbon hydrogen volumes will only be reached if strict emissions criteria are met.^9^^,^^44^^,^^70^^,^^74^^,^^135^ Furthermore, the design specifics of each production facility affect the process performance and therefore, the environmental footprint.^44^^,^^53^^,^^70^^,^^137^ Methods S3.2 presents the literature review results for SMR performance, with and without CCS, and specifics about LCI adaptations.

In contrast to GH_2_, we did not include the distributed production of BH_2_. Although small-scale SMR technologies are readily available,^63^^,^^139^ the distributed approach lacks the economies of scale to reduce its impact over the hydrogen total cost. Likewise, capturing CO_2_ from small-scale SMR facilities is expected to be harder and more expensive than from larger sources,^143^ whereas small-scale reformers will not have easy access to existing pipelines for transporting CO_2_ to the carbon storage location e.g., depleted gas and oil fields, or saline aquifers. Indeed, Wang et al.^124^ concluded that BH_2_ production will likely be located near large hydrogen consumers. Hence, we considered the distributed production of BH_2_ highly unlikely within the scope of this study.

Besides NG, we explored biomethane as alternative feedstock for reforming. Biomethane is produced by upgrading biogas, via amine scrubbing. This biogas was obtained via AD of biogenic waste. AD has been the dominant technology for biogas production in the EU,^82^ and it produces two outputs: biogas and digestates; additionally, it provides the service of waste management. Digestate could be applied in fields as fertilizer, but the actual amount of carbon sequestered depends on the specific of the agricultural practices, and the soil characteristics.^70^

We obtained the life cycle inventories (LCI) from Antonini et al.^70^ The two configurations selected for this study, one for NG and one for biomethane, represent SMR with low and high temperature water gas shift, while the CO_2_ capture is based on methyl-diethanolamine (MDEA) with 90% capture rate from the syngas,^70^ a representative value for current technologies.^135^ This LCIs originally dealt with electricity surplus as an avoided product. Aiming for consistency with attributional LCA guidelines we adjusted the LCIs so the electricity is considered as coproduct and energy allocation was performed. However, variations compared to the original approach were minimal as the surplus electricity is small.

Differences in process performance between the two feedstock configurations, NG and biomethane, are minimal, while the two feedstocks are comparable in terms of process efficiency.^70^ The modeling includes the transport and geological storage of CO_2_, over 200 km via pipeline reaching a saline aquifer at a depth of 800 m. We performed adaptations to the Ecoinvent 3.8 datasets to more adequately represent the methane emissions in NG supply chains. Furthermore, we removed the compression energy after the hydrogen leaves the reformer, which was originally included in the system boundaries in Antonini et al.^70^ since it does not belong to the hydrogen production stage outlined in this study. Details are presented in the Methods S3.2.

#### Vehicles

Hydrogen acts as energy carrier for both FCTs and ICETs. However, different strategies to guarantee energy storage and power supply during driving alter the specific truck components, resulting in different environmental footprints. Two factors are crucial for the transport sector, namely payload and range; the former typically determines the powertrain dimensioning, while range requirements define the amount of energy stored onboard.^19^^,^^57^

The different truck configurations in this study allow us to explore the environmental impacts of different design strategies that manufacturers are likely to adopt to address performance challenges. Firstly, achieving range competitiveness with conventional trucks implies storing enough energy onboard to reach a range of around 1,000 km. This is critical as refueling infrastructure is scarce or non-existent^2^^,^^51^^,^^54^ and long-haul trucks need more hydrogen and FC capacity to meet performance and daily range requirements compared to medium-haul trucks.^24^^,^^57^ To make matters worse, hydrogen has a low volumetric energy density compared to diesel: nearly one-fourth when liquid, and one-seventh when gaseous and compressed at 700 bar.^27^^,^^130^^,^^144^ Thus, we proposed truck configurations storing CH_2_ at 700 bar, inspired by Volvo’s approach,^145^ and a LH_2_ configuration, as in Daimler’s approach.^146^ For ICETs, the onboard hydrogen requirements are larger, given the lower powertrain efficiency compared to FCTs.^26^ In addition, we included cases for storage at 350 bar, a valid alternative for reducing CF use in the tanks. Methods S2.2.3 elaborates on tank estimation. Cryo-compressed storage systems were not evaluated in this study.

Using LH_2_ in trucks takes advantage of the higher volumetric energy density at the expense of facing boil-off in the tanks, caused by heat transfer, which results in tank pressurization, leading to venting. Studies suggest that venting could be diminished if idling periods are reduced^55^^,^^64^ or even could be rendered infrequent and negligible from a total cost of ownership perspective.^91^ In fact, venting could be recirculated into an FC to generate electricity.^64^ Despite the advantages, the energy required for reaching the liquefaction point at 20 K amounts to nearly one-third of the total energy available.^64^^,^^90^^,^^91^^,^^130^

In contrast, CH_2_ volumetric energy density is lower than that of LH_2_ and varies depending on the storage pressure. For compression to 700 bar the energy requirements are around 10%^56^ of total hydrogen energy, depending on the compression efficiency. Although hydrogen at 700 bar allows for rapid refueling, it requires pre-cooling at −40 C, due to temperature increase on the tanks.^2^^,^^51^^,^^56^ Furthermore, compression tanks contain energy intensive CF,^147^ estimated to cause up to 65% of the storage system GWP burden.^100^ CF was assumed to be produced in Germany, the largest market in the EU in 2023.^148^

Besides guaranteeing a minimum range, FCTs must address peak power dynamics, caused by steep topography or speed variations. As FC efficiency decreases at higher loads^149^ a LiB is included to reduce peak power demand from the FC during acceleration, optimizing operational efficiency,^2^^,^^19^ increasing the FC lifetime^19^^,^^149^ and enabling regenerative braking.^37^ However, it is unclear whether FCTs will be FC or battery dominant.

Thereby, we included two truck configurations: the first one powered by a 200 kW FC and a 140 kWh LiB, labeled as FCT_200_, while the second is powered by a 300 kW FC and a 40 kWh LiB, named FCT_300_. None of the FCT configurations are plug-in, meaning LiBs are only recharged via FC or regenerative braking. For ICETs the peak power is totally met by the engine since the lead-acid batteries are only for auxiliary purposes.

The modeled trucks are comprised of tractor and trailer combination. Tractor modeling was based on a bottom-up approach in which vehicle topologies were proposed for FCTs and ICETs, establishing a list of required subsystems e.g., hydrogen storage system, powertrain, body & chassis. Then, material composition was defined for each subsystem, see Methods S2.2.

Aiming for a fairer comparison between all vehicle configurations, the propulsion system of all powertrains delivers the same nominal power, 350 kW. Likewise, the trailer was kept the same for all truck configurations to ensure the same space for payload. Additionally, GVM was restricted according to EU regulations. In the EU, the limit weight of combination trucks (tractor + trailer), having five or six axles, is 40 ton^150^; however, for zero tailpipe emission trucks the permissible weight increases to 42 ton.^96^ As tractor configurations contain different components, tractor curb weights are different, meaning overall payload capacities are estimated to vary. Methodological choices, an index of subsystems and payload specifications are displayed in Methods S2.1 and S2.2.

As part of the SA, we explore cases in which the CH_2_ is stored at 350 bar as in most of the existing medium and heavy-duty FCTs.^26^ This reduces the energy required for compression and material costs as less CF is required^24^^,^^100^ while pre-cooling stage during refueling is eliminated.^26^ Nonetheless, a successful 350 bar storage system, feeding a 42 ton long-haul truck, designed for currently available truck platforms, is still to be proven as available volume onboard diminishes,^130^ given the increased number of tanks.^151^ Automotive experts consulted for this study disagree with this claim and see potential for 350 bar storage.^120^

To summarize, two of our FCT configurations store LH_2_, one is FC dominant while the other is battery dominant. In addition, we have two FCT configurations storing CH_2_ at 700 bar: FC dominant and battery dominant. For ICET, we include one configuration at 700 bar. In the same way, we will explore the storage of CH_2_ at 350 bar: two FCTs, one FC dominant while the other is battery dominant, and one ICET.

#### Transmission & distribution

There is a vast body of research on hydrogen delivery from an economic perspective,^49^^,^^61^^,^^131^^,^^134^^,^^152^ but also evaluating the technological status of transportation, storage, and refueling.^9^^,^^51^^,^^62^^,^^64^^,^^130^^,^^153^^,^^154^^,^^155^^,^^156^^,^^157^^,^^158^^,^^159^^,^^160^ There are also examples of LCA at the supply chain level^59^^,^^60^^,^^92^ with cases focused on underground storage.^161^ Likewise, there are studies estimating the GHG emissions and energy consumption for transportation.^56^^,^^63^^,^^152^

The most cost-effective way of delivering hydrogen depends on the amount and distance^2^^,^^49^^,^^61^^,^^63^^,^^64^^,^^131^^,^^162^ but also on the intended end use. Methods S4 includes a literature review on the characteristics of each transport method while also presenting the modeling data, assumptions and motivations for pathway selection. The selected pathways A to D were depicted in Figure 1.

The process of transporting hydrogen from the central plant to the HRSs is divided into transmission, until it reaches the GotHUB, and distribution, which refers to delivery to the HRSs. For transportation, hydrogen is either compressed or liquefied, a process also known as packing, which increases its volumetric density, significantly reducing the required volume for transportation and storage.^63^^,^^130^^,^^163^ Other methods for transporting hydrogen include its adsorption into metal hydrides or its use for the synthesis or liquid organic hydrogen carriers,^9^^,^^60^^,^^164^ which have been anticipated to be economically competitive for transport over long distances.^49^ These two methods are out of the scope of this study as only transportation of pure hydrogen is included.

The physical state in which hydrogen is transported, stored and finally pumped into the trucks defines the hydrogen delivery chain requirements. Multiple technically feasible methods for transportation are available, including ships, trailers and pipelines^55^^,^^60^^,^^64^^,^^159^^,^^162^ while combinations between them are usual. Each stage of storage, transportation, and packing requires energy,^56^^,^^152^ infrastructures^51^^,^^165^ and results in losses, irreversibilities and even hydrogen leaks.^40^^,^^64^

Pathway A assumes the hydrogen will be transported, in gaseous state, directly from the production plant to the HRS via tube trailer, without storage at the GotHUB. The tube trailer has a capacity of 1,000 kg H_2_ at 500 bar. Pathway B is analogous to pathway A as hydrogen is directly transported to the HRS, but this time in liquid state, in tank trailers, with 4,000 kg capacity. Pathways C and D include storage at the city gates in the GotHUB. This hub is proposed as a future hydrogen market is expected to require large storage facilities to guarantee supply for the region. An LRC is the selected storage method. Pathway C assumes that the hydrogen will come via pipeline to the GotHUB and then it will be distributed, via gaseous truck at 500 bar, to the HRS. Pathway D describes hydrogen produced in other countries (Chile for GH_2_ and Norway for BH_2_) and then transported, via tanker, to the GotHUB, then stored and distributed, always as LH_2_.

The transportation distance is uncertain as no exact location for future production plants has been defined. However, it will likely be located around the so-called hydrogen valleys, which are regions of high demand such as cities, industrial clusters, ports, and other commercial developments.^124^ Gothenburg region is a hub for technology and industry, home to the largest port in Sweden, and a solid location for laying pipelines^166^ which might eventually connect to European networks.^124^ Considering this, we assume the production facilities will be located 150 km away from the GotHUB while the distance from the GotHUB to the HRS is 50 km. For transportation via truck or ship, we included the burden of the empty return of the vehicles.

#### Use phase

The use phase encompasses hydrogen consumption and maintenance for FCTs and ICETs. For ICETs it also includes the tailpipe emissions and the AdBlue consumption used in the exhaust after-treatment system. All estimations represent a fully loaded truck but for the sake of simplicity we assume all truck configurations present the same fuel consumption per km. A higher load-factor is known to decrease the environmental burden when estimated per tkm^57^^,^^167^ while simultaneously increasing the results per vehicle km (vkm) due to higher hydrogen consumption.

Two estimations for hydrogen consumption are included; a base case, intended to represent the current performance of FCs, LiBs, and energy management strategies for the FCT. Parallelly, estimation for ICETs represents the current state of HPDI engines running on hydrogen while tailpipe emissions were set based on discussions with experts,^121^ and the legal limits imposed by EURO 7 regulation. Additionally, an optimistic estimation was included in the SA, where hydrogen consumption reduces as technology improves. Methodology for fuel consumption, emissions estimation, and vehicle maintenance are found in Methods S5.1–S5.4.

#### Sensitivity analysis

There are multiple sources of variability in the chosen cases. Two sets of variables were included in the SA, the first set represents changes in the modeled supply chains; we included efficiency improvements during hydrogen production, packing, and in hydrogen consumption onboard the trucks. For ICET pathways we explored different N_2_O tailpipe emission levels whereas for BH_2_ production we explored different rates of methane leakages in the NG supply chain. Additionally, the potential hydrogen leakages along the different pathways were included.

Moreover, the second set of variables explores more drastic changes in the modeled supply chains: the substitution of virgin CF by recycled CF in CH_2_ trucks and the replacement of conventional steel by steel based on DRI. Further details on the modeling approach are found in Methods S6. SA parameters are shown in Figure 4.

### Quantification and statistical analysis

This study does not include statistical analysis or quantification.

## Acknowledgments

Funding has been received through three Swedish competence centers: (1) The Competence Center Technologies and innovations for a future green Hydrogen economy (TechForH2) also hosted by Chalmers University of Technology, which is financially supported by the 10.13039/501100004527Swedish Energy Agency (P2021-90268) and the member companies 10.13039/100004741Volvo, 10.13039/501100018893Scania, Siemens Energy, 10.13039/501100014815GKN Aerospace, PowerCell, Oxeon, RISE, Stena Rederier AB, 10.13039/501100023964Johnson Matthey, Insplorion, and Manntek; (2) The Competence Center for Catalysis (KCK), which is hosted by Chalmers University of Technology and financially supported by the 10.13039/501100004527Swedish Energy Agency (Project No. 52689-1) and the member companies 10.13039/501100023964Johnson Matthey, Perstorp, PowerCell, 10.13039/100018558Preem, 10.13039/501100018893Scania CV, 10.13039/501100019931Umicore, and 10.13039/501100001730Volvo Group; and (3) The Swedish Electromobility center (SEC) founded by the 10.13039/501100004527Swedish Energy Agency in partnership with Swedish automotive industry and academia, funding the project “Fossil-free long-haul trucks in Europe” with grant number 12058. We would, further, like to thank the European Union Interreg Baltic Sea Region program for funding the project “Developing a transnational network of hydrogen refueling stations for trucks (HyTruck)” with grant number #C031. Moreover, financial support from 10.13039/501100001858Vinnova and the Swedish Energy Agency through the FFI Energy and Environment program funding the project “Hydrogen Engine Emissions Reduction (HEER)” with grant number 2020-016027 is greatly acknowledged.

## Author contributions

J.E.V.V. had a role in conceptualization, methodology, software, validation, investigation, data curation, writing (original draft, review, and editing), visualization, and project administration; S.B. and M.G. had a role in conceptualization, validation, writing (review), supervision, project administration, and funding acquisition; F.R. and D.B. had a role in validation.

## Declaration of interests

The authors declare no competing interests.
