# Supplementary material for: Vehicle-oriented and Sweden-framed life cycle assessment: Hydrogen for long-haul trucks
Source: iScience. 2025 Sep 19;28(10):113607. doi: 10.1016/j.isci.2025.113607 (PMC12538105; doi:10.1016/j.isci.2025.113607)
Supplement: Document S1. Figures S1–S6, Tables S1–S51, and Methods S1 [file mmc1.pdf]

iScience, Volume *28*

Supplemental information

Vehicle-oriented and Sweden-framed life cycle assessment: Hydrogen for long-haul trucks

Jorge Enrique Velandia Vargas, Selma Brynolf, Maria Grahn, Felipe Rodriguez, and David Blekhman

Methods S1: Technical background, estimations and assumptions

1. Literature review ... 3
2. The vehicles ... 6
   2.1. Modelling ... 8
   2.2. Truck subsystems ... 11
      2.2.1. Chassis and body ... 11
      2.2.2. Powertrain and transmission ... 11
      2.2.3. Hydrogen storage ... 12
      2.2.4. Fuel cell system ... 15
      2.2.5. Batteries ... 16
      2.2.6. Exhaust After-treatment ... 17
      2.2.7. Trailer ... 17
      2.2.8. Cooling, air and water management system ... 18
      2.2.9. Assembly ... 18
3. Hydrogen production ... 19
   3.1. Green hydrogen ... 19
      3.1.1. Electrolyzer performance ... 19
      3.1.2. Centralized production ... 22
      3.1.3. Distributed production ... 22
   3.2. Blue hydrogen ... 22
      3.2.1. Emissions sources ... 27
      3.2.2. The CO_2 path ... 27
      3.2.3. The current landscape ... 28
      3.2.4. Feedstocks ... 29
4. Transmission and distribution ... 35
   4.1. Packing ... 39
      4.1.1. Compression ... 39
      4.1.2. Liquefaction ... 39
   4.2. Transport ... 41
      4.2.1. Gaseous ... 41
      4.2.2. Liquid ... 45
   4.3. Storage ... 47
      4.3.1. Gaseous state ... 48
      4.3.2. Liquid form ... 50
   4.4. Purification ... 50
   4.5. Refueling station ... 51

4.5.1. Gaseous refilling ... 51
4.5.2. Liquid refilling ... 53
5. Use phase & end of life ... 54
5.1. Hydrogen consumption ... 54
5.2. AdBlue consumption ... 55
5.3. Tailpipe emissions ... 56
5.4. Maintenance & truck lifetime ... 56
5.5. End of life ... 57
6. Sensitivity analysis ... 58
6.1. Hydrogen leaks ... 58
6.2. Direct reduction of iron ... 63
6.3. Recycling of tanks ... 64
7. Biomethane comparison ... 65
8. Results ... 66
8.1. Climate change ... 66
8.2. Crustal scarcity indicator ... 72
8.3. Particulate matter-Environmental footprint ... 77
References ... 82

1. Literature review

The environmental footprint of fuel cell vehicle technologies has been frequently estimated and compared with the footprint of vehicles propelled by fossil fuels, biofuels and batteries; most of these studies focus on light duty vehicles (LDVs) [1–6] and usually evaluate carbon footprint exclusively. Research has identified the hydrogen production stage as a dominant contributor to the total life cycle impacts while emphasizing the relevance of the electricity source, for electrolysis, and the raw material containing the hydrogen, for thermochemical processes like steam methane reform (SMR) [7–9].

In contrast, life cycle assessment (LCA) studies for hydrogen-propelled heavy-duty vehicles (HDVs) are less frequent and mainly focused on carbon footprint estimation also. Ricardo [10] was the only identified study to perform LCA, for both LDV and HDV, for multiple powertrain technologies. Only a handful of studies present a Cradle-to-Grave perspective [11–13] while others evaluate emissions on a Well-to-Wheel (WTW) perspective[14–16]. Lee et al.[14] estimated that switching from conventional diesel internal combustion engine trucks (ICET) to fuel cell trucks (FCTs) would reduce lifecycle fossil fuel consumption, greenhouse gas (GHG) emissions, and air pollutants emissions while Liu et al.[15] found that, even for Natural gas (NG)-based SMR, fuel cell LDVs exhibit lower WTW fossil energy use and lower WTW GHG emissions. However, although emissions and energy consumption are included throughout several life cycle stages, no information about the global warming potential (GWP) characterization method was disclosed in either Lee et al. [14] or Liu et al. [15].

Furthermore, Sacchi et al.[17] studied the effect of load factor and range on the GWP of using HDVs on current and future scenarios and concluded that, by 2040, battery electric trucks (BET)s and fuel cell trucks (FCT)s are promising options if a low GHG intensity of electricity is achieved. Moreover, no LCA studies for hydrogen-powered ICETs were found. Table 1 presents the literature review on LCA studies of hydrogen-propelled trucks. Nevertheless, as the functional unit, time and geographical scope, and system boundaries vary, caution is advised while drawing conclusions from the direct comparison of the studies.

There is also evidence of studies focusing on the environmental footprint of specific stages of the hydrogen supply chain. For instance, Frank et al.[18] performed a techno-economic and life cycle emissions model for hydrogen production and delivery pathways while the European Commission [19] performed an LCA of hydrogen delivery options within Europe. Similarly, Tayarani & Ramji [20] performed an LCA of hydrogen production pathways and delivery including liquefied hydrogen (LH_2) and compressed hydrogen (CH_2) whereas Lotrič et al.[21] focused on hydrogen production via electrolysis and their consequences in terms of EU critical raw materials use. A further detailed review of studies estimating energy consumption and emissions associated with green hydrogen (GH_2) and (BH_2) is presented in Section 3.

Other researchers have aimed at performing LCA of vehicle components or technologies: Weiszflog & Abbas [22] performed an LCA for the hydrogen storage system onboard FCTs, including CH_2 at 700 bar, LH_2, and cryo-compressed hydrogen, quantifying the GWP impacts linked to carbon fiber (CF) tanks. Franz & Liljenroth [23] presented the life cycle inventories (LCIs) of an automotive fuel cell system (FCS) based on a commercially available model [24]. Wolff et al [25] developed a study intended to provide scalable life cycle inventories modelling ICETs, BET, and hybrid electric trucks. Iyer et al. [26] presented detailed datasets for medium duty vehicles and HDVs, including three different FCTs, which are included in GREET model [27]. However, this study represents vehicles intended for the U.S. market: heavier and larger than their European counterparts. Usai et al. [28] suggested that fuel cells exhibit potential for reduction of environmental footprint considering the current high learning rates and the possibility of using renewable energy. Joint Research Centre [8] adapted ICET data to model FCTs and BETs for medium-haul ranges, in the European context, but data regarding the specific truck subsystems was not disclosed. Simons and Azimov [29] compared the specific life cycle impacts of the HDV propulsion systems, including fuel cells and batteries.

A lack of information on the environmental footprint of some supply chain processes such as storage or purification was identified in the literature review while similar conclusions were achieved by the European Commission [30]. This data gap extends to the hydrogen transportation via pipeline, and via liquid tankers. As lack of harmonized data prevents accurate comparisons between studies. It has been proposed that LCA studies should report the physical state, purity, temperature and pressure, production flow rate and even impurities [8]. In consonance, Valente et al.[7] stated the importance of disclosing the allocation criteria, heating value, purity, the use of capital goods, and expressing the results per life cycle stage. Hydrogen production performance parameters are seen in Section 3.

Table 1. Literature review results

Reference	System boundaries	Time scope	Geographical scope	Functional unit	Impact categories	Vehicles	Gross vehicle mass & curb weight	Range (km)	H_2 onboard capacity (kg)
ICCT (2023)	Cradle to grave	2020 & 2030	EU (grid average)	1 travelled km	Global warming (GWP IPCC 2013)	ICETs , BETs & FCTs	12 t & 4.2 t<br>40 t & 14.9t	12 t (200-250)<br>40 t (500)	12 t (20)<br>40 t (45)
Liu (2020)	Well-to-Wheel	2018	U.S.	1 travelled mile	GHG emissions. Fossil energy use. CO & NOx emissions	LDVs	-	-	-
Lee (2018)	Tank-to-Wheel & Well-to-Wheel	2016	U.S. Average and 8 regional grids	1 travelled mile	GHG emissions, Fossil fuel use, Air pollutants (VOC, CO, NOx, $PM_{2.5}$ & PM_{10}).	7 types of MDVs and HDVs	20.5 t & 10.21 t	>350 miles	Unspecified
JEC (2020)	Well-to-Wheel	2016 & 2035	EU	1 tkm	GHG emissions	ICETs, BETs, FCTs. Includes Type 4 (up to 18 ton) and type 5 (up to 40 ton) trucks.	40 t & 6.86 t	614	40 t (42)
Sacchi (2021)	Tank-to-Wheel	2020-2050	EU	1 travelled km & 1 tkm	Global warming (GWP IPCC 2013)	ICETs, BET and FCT	40 t & 14.5 t	800	66 kg (2020 case)
Drawer (2024)	Cradle to grave	Unspecified	EU	1 tkm	GHG emissions, Mineral resource scarcity (Recipe 2016)	FCT build based on a second-life diesel truck	32.1 t & 10.1 t	Unspecified	Unspecified
Booto (2021)	Cradle to grave	Unspecified	Norway	1 travelled km	GWP, abiotic resource depletion potential, acidification potential, human toxicity potential (CML 2001)	ICETs, BET and FCT	12 t & unspecified	300	Unspecified

Table 1 continued. Literature review results

Reference	Onboard H_2 state	H_2 production	H_2 transport	Results (GWP per Functional Unit) - Only FCTs are included	Notes
ICCT (2023)	Gaseous (unspecified pressure) & Liquid	Grey H_2 [a]<br>Green H_2 [b]<br>50% green 50% blue [c]	Onsite production	2021-2040 scenario:<br>1,080gCO_2eq CH_2 [a]; 1,233gCO_2eq LH_2 [a]; 190g CO_2eq CH_2 [b]<br>2030-2049 scenario:<br>240gCO_2eq CH_2 [c]; 175g CO_2eq CH_2 [b]	Same tank for onboard storage of liquid and gaseous. Infrastructure for vehicle production, recycling and refueling not included.
Liu (2020)	-	Grey H_2<br>Grid-powered electrolysis (U.S. average and California)	CH_2 via tube-trailer & LH_2 via cryogenic tanker	-	LCIA performed for GHG only.
Lee (2018)	Gaseous-350 bar & Liquid	Grey H_2 [a]<br>Green H_2 as sensitivity analysis [b]	Average U.S. conditions	1,400gCO_2eq CH_2 [a]; 2,000gCO_2eq LH_2 [a]; 200gCO_2eq CH_2[b]	Not a proper LCA in the strict definition. Data represents a tractor-trailer. Other vehicles are included in the study.
JEC (2020)	Gaseous 700 bar	[a] Central electrolysis, wind<br>[b] Central electrolysis, coal electricity<br>[c] Elec EU mix, electrolysis<br>[d] SMR NG (NG transported 4000 km)	[a] Pipeline<br>[b] Road LH_2<br>[c] On-site<br>[d] On-site	2016 scenario:<br>6gCO_2eq [a]; 290gCO_2eq [b]; 102gCO_2eq [c]; 68gCO_2eq [d]<br>2030 scenario:<br>6gCO_2eq [a]; 240gCO_2eq [b]; 58gCO_2eq [c]; 52gCO_2eq [d]	Two separate studies were published: Well to Wheel analysis and Tank to wheel analysis.
Sacchi (2021)	Gaseous 350 bar	[a] Electrolysis, EU mix<br>[b] Electrolysis, Hydro<br>[c] Electrolysis, solar PV<br>[d] SMR, NG<br>[e] SMR, NG with CCS	Not specified	per tkm:<br>75gCO_2eq [a]; 26gCO_2eq [b]; 37gCO_2eq [c]; 74gCO_2eq [d]; 45gCO_2eq [e]<br>per km:<br>270gCO_2eq [b]	Diesel, Diesel hybrid, plug-in diesel hybrid, compressed NG, FCT and BETs are included. Seven size classes included. Three application ranges. Presented GWP for 40t truck.
Drawer (2024)	Gaseous 700 bar	[a] Electrolysis, German mix<br>[b] Electrolysis, wind & solar	Not specified	93gCO_2eq [a] ; 33gCO_2eq [b]	Diesel trucks converted into FCTs. Credits for reusing the Diesel truck.
Booto (2021)	Gaseous 700 bar	SMR & Electrolysis with electricity from hard coal	On-site production and storage	477g CO_2 eq	No battery included in the FCT. Fuel consumption represents an empty truck.

2. The vehicles

The trucks in this study are intended for long-haul transportation of goods in Sweden. However, as trucks in Europe usually operate within the common market, the vehicle needs to fulfill the dimensional and weight regulatory restrictions applied in the EU. Specifically, a 24 m length constraint applies to truck trailers [31], while the maximum allowed weight is defined by the number of axles [31,32]. We selected a long-haul combination vehicle (5 axles and more) as representative of long-haul transportation in Sweden. These vehicles are known in different countries as semi-trailer trucks, big rigs, 18 wheelers, prime movers, or lorries. Here we refer to the vehicle merely as truck and it comprises of tractor and trailer.

The tractor unit refers to the towing engine vehicle that provides power for hauling a given load whereas the trailer is the unpowered vehicle which encases the load and is towed by the tractor, as portrayed in Figure 1. Tractor units in FCTs and ICETs rely on different powertrain technologies. Thus, the material composition and onboard subsystems vary. Aiming to evaluate the life cycle impacts linked to diverse tractor configurations we included 6 versions of the FCT and 2 of the ICET.

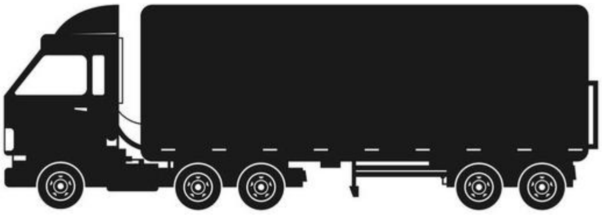

Figure 1. Basic scheme of a tractor (left) and trailer (right) combination.

The different truck versions in this study are intended to represent different design strategies to address key challenges in truck design. Firstly, whether to store hydrogen as LH_2 or as CH_2. Then, if CH_2 is chosen, what will be the storage pressure; 350 bar or 700 bar. And finally, how to deal with peak power dynamics; in other words, what is the proportion of fuel cell capacity compared to battery capacity. These power demand dynamics are caused by steep road topography and acceleration during driving. Two FCT versions address this question; the first one depicts a 200 kW fuel cell and a 140 kWh Li-ion battery (LiB), labelled as FCT_{200}, while the second has a 300 kW fuel cell and a 40 kWh LiB, labeled as FCT_{300}. This methodology allows us to estimate the environmental footprint differences linked to different truck configurations. The truck versions are listed in Table 2.

As each truck version has a specific layout of components - or subsystems- in the tractor, the curb weight (CW) also varies. The CW is defined as the weight of the vehicle, including a full tank of fuel and the standard equipment; however, it does not include any passengers or cargo. In contrast, all truck versions have the same trailer. By assuming the same trailer, we guarantee that the volume for payload positioning is identical. In addition, all the truck versions display 350 kW traction power.

The GVM is the maximum operating weight that the fully loaded truck is allowed to transport, as specified by the local regulation. Thus, the GVM is effectively the CW plus the payload and additional accessories (*e.g.*, canopies or additional lamps). We did not include any additional accessories. In the EU, the weight limit of combination trucks (tractor + trailer), having five or six axles, is 40 t [31], however, for zero tailpipe emission trucks the permissible weight increases to 42 t [33,34]. The GVM for truck configurations is 42 t. Payload capacity is shown in Table 4.

Table 2. List of truck versions included in this study.

Hydrogen storage	Gaseous form (700 bar)			Gaseous form (350 bar)			Liquid	
Powertrain	FCT_{200}	FCT_{300}	ICET H_2	FCT_{200}	FCT_{300}	ICET H_2	FCT_{200}	FCT_{300}

Table 3. Index of the different truck components for each different vehicle configuration.

		FCT_{200}	FCT_{300}	ICET H_2	Reference (weight)	Reference (bill of materials)
H_2 system	Fuel cell stack	✓	✓	×	Powercell [24], Franz & Liljenroth [23]	Argonne, national lab [27]
	Balance of plant	✓	✓	✓	Weiszflog & Abbas [22]	Weiszflog & Abbas [22]
	Hydrogen tanks	✓	✓	✓	Weiszflog & Abbas [22], complemented with own modelling for 350 bar	Weiszflog & Abbas [22], complemented with own modelling for 350 bar
	Frame (for H_2 tank)	✓	✓	✓	Weiszflog & Abbas [22]	Weiszflog & Abbas [22]
	Safety frame for fuel cell system	✓	✓	×	Volvo experts [35]	Volvo experts [35]
	Cooling, water & air management system	✓	✓	✓	Own estimation	Own estimation
ICE powertrain	Combustion Engine	×	×	✓	JRC [8]	Volvo experts [35]
	Diesel tank	×	×	×	-	-
	AdBlue tank	×	×	✓	JRC [8]	Own estimation
Transmission	Transmission	✓	✓	✓	Argonne, national lab [27]	Argonne, national lab [27]
Electric powertrain	Inverter	✓	✓	×	Nordelöf et al. [36]	Nordelöf et al. [37]
	Converter	✓	✓	×	Nordelöf et al. [36]	Nordelöf et al. [36]
	Electric motor	✓	✓	×	Nordelöf et al. [37]	Nordelöf et al. [37]
Batteries	Lead-acid battery	×	×	✓	Argonne, national lab [27]	Argonne, national lab [27]
	Li-ion battery	✓	✓	×	Ellingsen et al [38]	Ellingsen et al [38]
	Chassis & body	✓	✓	✓	Argonne, national lab [27], own estimation	Argonne, national lab [27]
	Trailer	✓	✓	✓	Argonne, national lab [27]	Argonne, national lab [27]

FCT_{200}: Includes a 200 kW fuel cell & 140 kWh LiB; FCT_{300}: Includes 200 kW fuel cell & 140 kWh LiB; Cooling system is required for both fuel cell stack and ICE. For the LH_2 trucks the balance of plant was modelled as in the GH_2 version due to lack of data.

2.1. Modelling

All truck versions were built based on a bottom-up approach which initially identified the vehicle subsystems as seen in the FCT and ICET topologies in Figure 2. Subsequently, based on scientific literature or suggestions from experts, we proceeded to define the weight and material composition of each subsystem. The material composition is also known as the bill of materials (BoM). The adopted topologies are expected to be valid approximations for FCTs and ICETs but are not intended to be an exact representation of the truck layout.

Indeed, topology variations might occur depending on the design criteria adopted by each automaker. Table 3 exhibits the references for the BoM and subsystem weight in each truck version. For obtaining the BoM we relied on the literature, especially the data included in GREET model [27], Joint Research Centre [8] and also discussions with automotive sector experts[35]. Finally, the results were then validated and discussed with Volvo specialists [35]. Table 4 presents the component weight for each truck version and the manufacturing locations assumed for this study. Our intention is to reflect current trends in the automotive manufacturing industry where some of the subsystems are manufactured abroad and then transported for assembly.

Adapting the literature data to model a truck suitable for use in Sweden and the EU requires recognizing that the trucks represented by GREET datasets [27] are trucks representative of the US market and not the EU market. The trucks manufactured for the US and the EU markets are different. For instance, European models display a cab-over style, with the cabin of the truck sitting above the front axle and engine, in contrast to US models, where the engine is mounted in front of the cabin, also known as bonnet-style.

This is due to the particular needs associated with each market. Transportation distances as well as driving shifts are often longer in the US. Most truck drivers in Europe are allowed by law to drive for 8-9 hours a day, in contrast to 12 hours per day in the US. Indeed, drivers in the US usually live for long periods in their trucks, requiring extra features in the cabin for comfort. Moreover, although European highways are wide enough for trucks, when they leave the highway and enter urban areas, roads present turns and narrower streets, requiring better maneuverability. Thus, trucks in Europe are more compact, lighter, and have a shorter wheelbase.

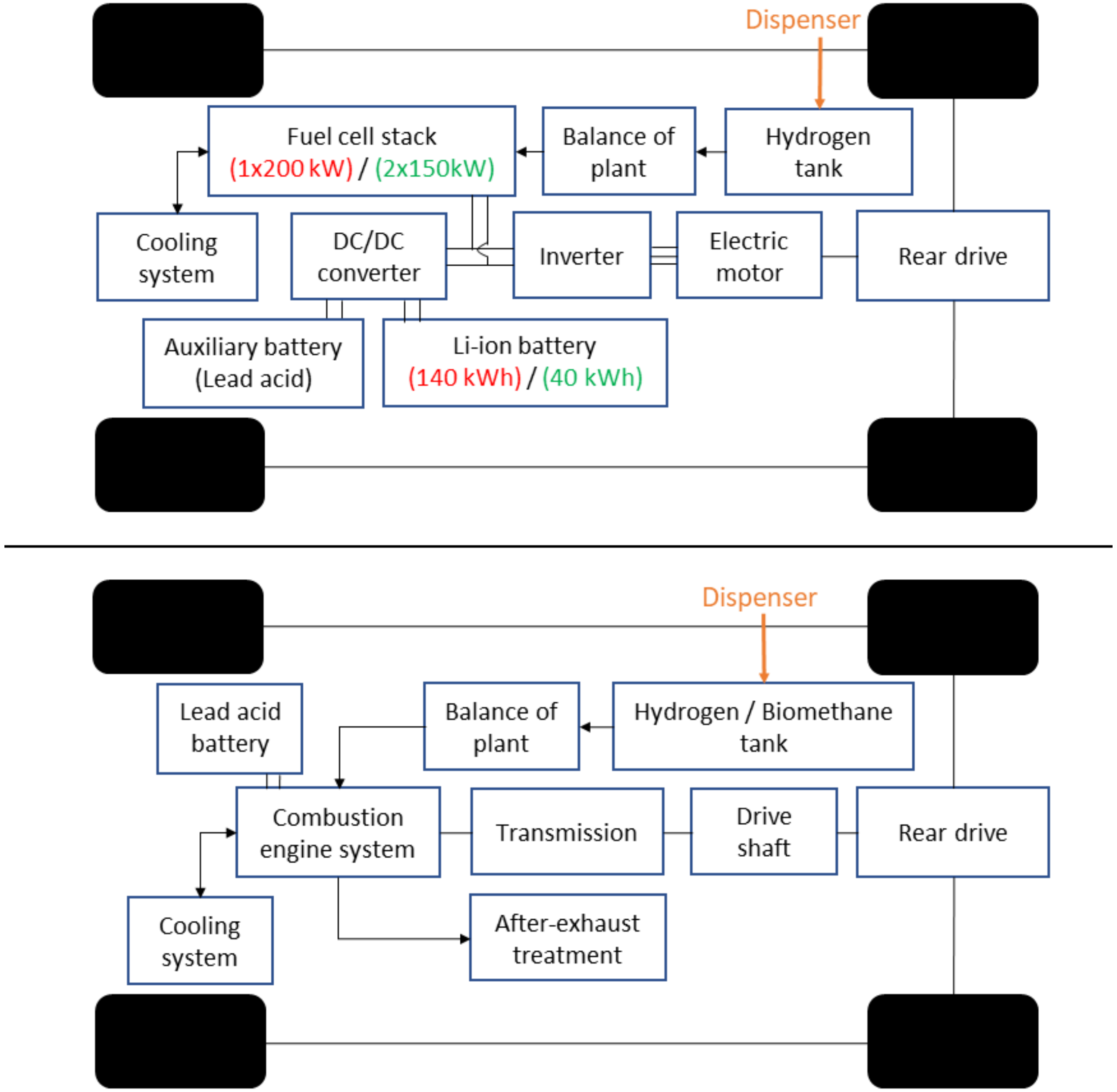

Figure 2. Topology for fuel cell trucks (top) and internal combustion engine trucks (bottom). For fuel cell trucks, battery and fuel cell capacity are shown in different colors. FCT_{200} configuration is shown in red while FCT_{300} configuration is presented in green.

Table 4. Component mass (kg) for each truck configuration.

	Component	Gaseous H_2 (700 bar)			Gaseous H_2 (350 bar)			Liquid H_2		Location
		FCT_{200}	FCT_{300}	ICET H_2	FCT_{200}	FCT_{300}	ICET H_2	FCT_{200}	FCT_{300}	
H_2 system	Fuel cell stack system	424	636	-	424	636	-	424	636	Germany
	Balance of plant (tank)	110	110	110	110	110	110	200	200	Germany
	Hydrogen tank	1,475	1,475	1,770	1,042	1,042	1,190	687	687	Germany
	Frame for H_2 tanks	1,120	1,120	1,344	784	784	904	480	480	Sweden
	Fuel cell stack cage	100	100	-	100	100	-	100	100	Sweden
Cooling system	Cooling system*	250	300	100	250	300	100	200	250	Sweden
ICE powertrain & fuel	I.C. Engine	-	-	1,160	-	-	1,160	-	-	Sweden
	Exhaust aftertreatment	-	-	261	-	-	261	-	-	Sweden
	AdBlue tank	-	-	15	-	-	15	-	-	Sweden
	Diesel tank	-	-	-	-	-	-	-	-	Sweden
Transmission	Transmission	186	186	428	186	186	428	186	186	Sweden
Electric powertrain	Motor	173	173	-	173	173	-	173	173	Germany
	Converter	40	40	-	40	40	-	40	40	Germany
	Inverter	31	31	-	31	31	-	31	31	Germany
Batteries	Li-ion Battery	900	267	-	900	267	-	900	267	China (cells)
	Lead acid batteries	31	31	130	31	31	130	31	31	Germany
	Body & chassis	5,800	5,800	5,800	5,800	5,800	5,800	5,800	5,800	Sweden
	Trailer	7,500	7,500	7,500	7,500	7,500	7,500	7,500	7,500	Sweden
	**Only tractor**	10,640	10,269	11,675	9,871	9,500	10,453	9,252	8,881	
	**Fluids (w/o fuel)**	168	168	240	168	168	240	168	168	
	**Driver**	80	80	80	80	80	80	80	80	
	**Gross vehicle mass****	10,888	10,517	11,439	10,119	9,748	10,419	9,500	9,129	
	**Estimated payload**	23,612	23,983	23,061	24,381	24,752	24,081	25,000	25,371	

*Cooling system for FCT_{200} & FCT_{300} includes the air management system and the water management system.

**Fuel not included in GVM.

2.2. Truck subsystems

2.2.1. Chassis and body

The chassis is the central supporting element of the truck and consists of two longitudinal beams, steer and drive axles, suspensions, wheels and tires, fifth wheel and auxiliaries. Moreover, the body refers to the rest of the vehicle structure, which encases and supports the mechanical and operational components and provides shelter for the driver. The body comprises interiors and exteriors, including glass, but excluding the power transmission components and energy storage units.

Initially, the weights for the chassis and body were taken from the Sleeper-cab truck, intended for the US market, included in GREET [27]. As both chassis and body are likely to be lighter in the European version, we adjusted the chassis weight to 4,400 kg and the body weight to 1,400 kg. Table 5 and Table 6 depict the body BoM and the chassis BoM and the Ecoinvent v3.8 processes used for modelling.

Table 5. Bill of materials for the truck body and Ecoinvent processes used for modelling.

Input	%	Provider
Cast Aluminum	3.5	market for aluminium, cast alloy | aluminium, cast alloy GLO
Wrought Aluminum	10.4	market for aluminium, wrought alloy | aluminium, wrought alloy GLO
Silica	0.1	Silica sand production | ceramic tile DE
Copper	0.3	market for copper cake | copper cake GLO
Damask	3.2	fleece production, polyethylene | fleece, polyethylene RER
Leather	1.7	fleece production, polyethylene | fleece, polyethylene RER
Glass fiber-R. plastic	15.4	glass fibre production | glass fibre RER
Graphite	0.1	graphite production | graphite RER
Latex	2.5	market for latex | latex RER
Plastic	18.1	polypropylene production, granulate | polypropylene, granulate RER
Steel, stainless	2.4	market for steel, chromium steel 18/8 | steel, chromium steel 18/8 GLO
Steel	31	market for steel, low-alloyed | steel, low-alloyed GLO
Rubber	1.9	synthetic rubber production | synthetic rubber RER
Glass	9	tempering, flat glass | tempering, flat glass RER

Table 6. Bill of materials for the truck chassis and Ecoinvent processes used for modelling.

Input	%	Provider
Cast Aluminum	5	market for aluminium, cast alloy | aluminium, cast alloy GLO
Cast iron	7.5	market for cast iron | aluminium, wrought alloy GLO
Plastic	0.1	polypropylene production, granulate | polypropylene, granulate RER
Steel	78	market for steel, low-alloyed | steel, low-alloyed GLO
Rubber	9.4	synthetic rubber production | synthetic rubber RER

2.2.2. Powertrain and transmission

The powertrain includes the devices that generate and deliver power to the wheels. It incorporates the drivetrain mechanisms which are in charge of converting and transmitting torque to the wheels. For the ICETs we included the engine unit, the fuel storage, the exhaust system, and the emission control electronics. For FCTs it includes the electric motor, converter, and inverter. Transmission elements were also incorporated.

The motor capacity for all truck versions was estimated as 350 kW, based on JRC [8], and validated during personal communications with automotive industry experts[35]. For the sake of simplicity, we decided to keep the same motor capacity for all truck versions despite their GVM differences. Material composition for the motor and the inverter was based on Nordelöf et al. [36,37]. For the inverter, we assumed that a 10% overcapacity (adding up to 385 kW) was required to cope with potential power peaks while voltage was assumed as 600 V. For converter weight we appealed to JRC [8] while BoM was taken from Nordelöf et al.[36].

Automatic transmission is included for both FCTs and ICETs. Transmission weight between ICETs and FCTs vary, and BoM was modelled as in GREET [27]. Table 7 depicts the transmission BoM and the Ecoinvent v3.8 processes used for modelling while Table 8 exhibits the ICE data.

Table 7. Bill of materials for the truck transmission and Ecoinvent processes used for modelling.

**Input**	**%**	**Provider**
Magnet	0.2	market for permanent magnet, for electric motor GLO
Wrought Aluminum	0.1	market for aluminium, wrought alloy | aluminium, wrought alloy GLO
Plastic	5.4	polypropylene production, granulate | polypropylene, granulate RER
Steel	86.3	market for steel, low-alloyed | steel, low-alloyed GLO
Rubber	0.6	synthetic rubber production | synthetic rubber RER
Cast iron	7	market for cast iron | aluminium, wrought alloy GLO
Copper	0.4	market for copper cake | copper cake GLO

Table 8. Bill of materials for the ICE and Ecoinvent processes used for modelling.

**Input**	**%**	**Provider**
Cast Aluminum	3.5	market for aluminium, cast alloy | aluminium, cast alloy GLO
Wrought Aluminum	3.5	market for aluminium, wrought alloy | aluminium, wrought alloy GLO
Cast iron	50	market for cast iron | aluminium, wrought alloy GLO
Steel	35	market for steel, low-alloyed | steel, low-alloyed GLO
Ceramic	2	market for ceramic tile | ceramic tile | Cutoff, U - GLO
Copper	1	market for copper cake | copper cake GLO
Plastic	4	polypropylene production, granulate | polypropylene, granulate RER
Rubber	1	synthetic rubber production | synthetic rubber RER

2.2.3. Hydrogen storage

The hydrogen storage system comprises storage tanks, auxiliary components for delivering hydrogen to the fuel cell stack -also known as balance of plant (BoP)- and a safety frame that protects the hydrogen tanks from potential impacts and attaches them to the chassis & body. Modelling data for the CH_2 tanks at 700 bar and the LH_2 storage tanks was obtained from Weiszflog & Abbas [22], while carbon fiber was assumed to be produced in Germany, the largest carbon fiber market in the EU in 2023 [39]. The system boundaries in Weiszflog & Abbas [22] encases the system from the hydrogen filling receptacle to the point where hydrogen is delivered to the fuel cell stack.

CH_2 tanks at 700 bar (70 MPa) are type IV, which indicates the presence of a polymer inner liner, that acts as a hydrogen permeation barrier, overwrapped by carbon fibers in an epoxy matrix and a cap, also known as boss [22,40]. As a safety precaution, hydrogen tanks incorporate a pressure relief valve that vents gas to avoid tank rupture in case of overpressure [40]. The current state of the art for tanks in LDVs is 700 bar, while urban bus fleets often exhibit 350 bar carbon fiber tanks [40].

For the 350 bar tanks we modelled the BoM assuming capsule-shaped tanks with 33 cm radius (r) and 134 cm height (h), giving a total length (l) of 200 cm, see Figure *3*. The wall thickness of the 700 bar tank was assumed to be 41 mm based on discussions with industry partners. Based on this wall thickness we calculated the strength of the material using Barlow's formula. We assumed the same materials for the 700 bar and 350 bar tanks and calculated the needed wall thickness for the 350 bar case based on the maximum allowed stress of the material. The weight of the carbon fiber reinforced polymer (CFRP) is based on a density of 1.75 g $cm^{3\ -1}$. The ratio of carbon fibers to epoxy is 3:2 [22].

Furthermore, we estimated the amount of liner needed considering the tank inner radius, a liner thickness of 2 mm, and a density of 0.97 g $m^{3\ -1}$. The tank inner liner is made from high density polyethylene (HDPE) via injection molding while the boss is aluminum-based. In terms of weight, the most significant contributor is the

external layer made of CFRP [22]. One boss per tank is included with an estimate weight of around 2.5 kg per boss based on the 700 bar tank in Weiszflog & Abbas [22].

The number of tanks needed for each FCT configuration was estimated based on the inner volume of the tanks and the density of compressed hydrogen at 20 °C of 38.7 kg m^{3-1} and 23.7 kg m^{3-1} for the 700 bar and 350 bar respectively. Five tanks were estimated to be required for the 700 bar FCT as in Weiszflog & Abbas [22] while ,for the 350 bar case, seven tanks were estimated to be needed to keep a range of 1000 km. The calculations are summarized in Table *9* and a summary of the BoM and the Ecoinvent datasets is presented in Table 10.

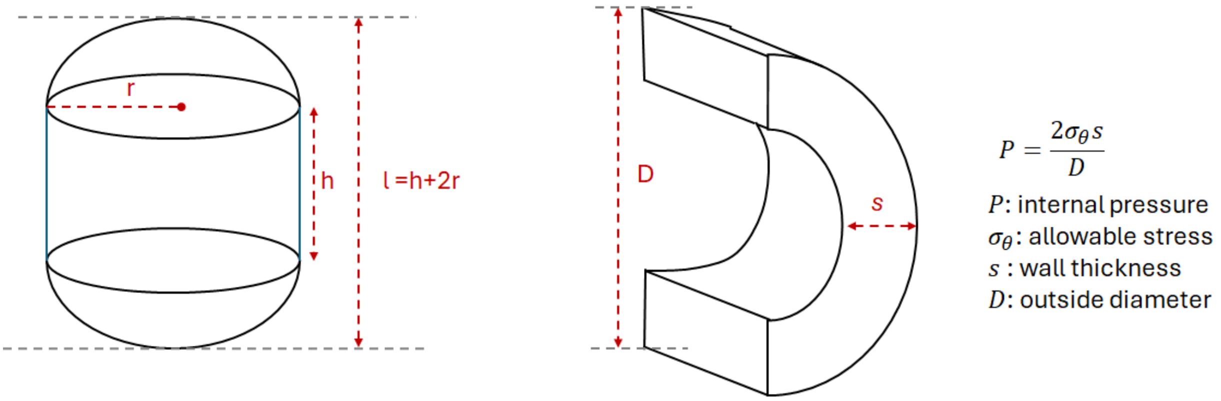

Figure 3. Illustration of the dimensions of the tanks and Barlow's formula.

Table 9. Summary of data characteristics used in the estimation of FCT tanks for 700 bar and 350 bar tanks.

	Tank 700 bar	Tank 350 bar	Unit
Outer diameter (cm), 2r	66	66	cm
Total length (cm), h+2r	200	200	cm
Thickness carbon fiber +epoxy resin	4.1	2.05	cm
Thickness liner	0.2	0.2	cm
Density H2, 20 degree C	38.73	23.69	kg/m3
Total amount of H2 on truck	80	80	kg
Volume of tanks w & w/o thickness	2.07	3.38	m3
Number of tanks	5	7	
Volume carbon fiber + epoxy resin per tank	0.16	0.07	m3/tank
Total volume carbon fiber + epoxy resin	0.78	0.52	m3
Estimated specific weight carbon fiber + epoxy resin	1.75	1.75	g/cm3
Estimated specific weight carbon fiber + epoxy resin	1750	1750	kg/m3
Total weight carbon fiber + epoxy resin	1367	905	kg
Volume liner per tank	0.01	0.01	m3/tank
Total volume liner	0.035	0.054	m3
Liner density	970	970	kg/m3
Boss weight per tank	2.5	2.5	kg/tank
Total boss weight all tanks	10	17.6	kg
Total weight of the tanks	1411	1042	kg

HDPE Liner weight was kept as 119 kg as in Weiszflog & Abbas [22]

Table 10. Material composition for the 700 bar and 350 bar tanks and Ecoinvent processes used for modelling.

Component	700 bar (%)	350 bar (%)	Provider
Carbon fiber	58	56	Benitez et al. [6]
Epoxy resin	39	37	market for epoxy resin | epoxy resin, liquid - RER
HDPE liner	2	5	Benitez et al. [6]
Boss	1	1	Weiszflog & Abbas [22]

Compared to 700 bar, the 350 bar storage tanks are expected to require less CF per tank [27]. However, if the same amount of hydrogen were to be stored onboard at a lower pressure, more tanks, and therefore, more volume onboard would be required for tank placement. Considering the length restrictions for trucks in the EU [32,41], this might reduce space onboard for other systems.

Despite this constraint, we assumed the same amount of hydrogen (80kg) could be stored in the 350 bar system while still respecting the maximum truck dimensions and weight. This decision was made based on conversations with automotive experts[35] which stated that improvements in design have the potential to deliver a truck able to pack the additional tanks in such a way that the same amount of hydrogen is stored, compared to the 700 bar case, while also observing the regulatory constraints [42]. However, we highlight the uncertainties regarding tank pressure and how it could affect fuel consumption and payload capacity.

In contrast to CH_2 storage systems, the LH_2 system maintains the fuel in liquid form, at -253 °C or 20 K. This is advantageous for automotive applications as LH_2 has an energy density nearly twice that of compressed hydrogen at 700 bar [43,44]. However, LH_2 storage tanks necessitate exceptional thermal insulation to keep the hydrogen in liquid state [45]. Despite this, some heat will always seep through into the tank causing the LH_2 temperature to increase and vaporize [40,46,47]. To prevent excessive pressure inside the tank some of the vaporized hydrogen must be released when the pressure reaches a critical threshold [48]. This is concerning from an economic viewpoint as it implies venting expensive hydrogen while also raising serious environmental concerns, as the GWP of hydrogen has come into discussion [49–51].

The LH_2 vessels are comprised of an inner liner, an external protective layer, and a boss[22]. In addition, there is insulation between the outer part and the inner liner provided by vacuum and aluminum layers. LH_2 tanks are primarily made of steel, and they do not need the CFRP required for withstanding high pressures in CH_2 tanks. Although hydrogen could negatively affect the mechanical properties of austenitic stainless steels [52], the ultimate stress in austenitic stainless steel is high enough for the structural integrity not to be compromised [48]. The LH_2 tanks were modelled as in Weiszflog & Abbas [22].

After the hydrogen leaves the tank, it is delivered to the fuel cell under specific conditions. For instance, it needs to reach at least -40 °C before entering the fuel cell system; hence, LH_2 tanks require a heat exchanger at the exit point [22]. In addition, in case the tank pressure drops below 6 bar, the hydrogen is gasified by heaters that might be integrated in the cooling system of the vehicle [22]. Thus, the BoP will likely comprise of heat exchangers, heaters, pipes, valves, etc. Nonetheless, the exact composition of the BoP is uncertain and depends on the specific system design; hence, component variations are expected. For instance, Ahluwalia et al. [48] proposed two different BoP systems; one including pumps and one without pumps.

We found no BoM describing the BoP for LH_2 trucks in the scientific literature. In this study, we included the BoP material composition as in a 700 bar gaseous hydrogen truck. This is a rough proxy as such systems are expected to be different; for starters, the BoP of LH_2 storage needs to deal with a large temperature gap between the tank and the fuel cell inlet whereas the BoP for the gaseous storage deals with a large pressure drop. We addressed these variations by proposing different BoP weights as seen in Table *4*. However, both CH_2 and LH_2 BoP were assumed to have the same materials: stainless steel, aluminum, and rubber.

Regarding the boil-off in LH_2 trucks, Ahluwalia et al. [48] discussed the possibility of completely avoiding venting losses in LH_2 vehicles if idling periods are short. This way, the driving ends up consuming all the evaporated hydrogen before the tank pressure reaches the relief limit. This is favorable for the long-haul segment as the trucks are not expected to idle for long periods, and they operate year-round [53]. Additional boil-off could be used for electricity generation in the fuel cell, for powering other vehicle functions.

The hydrogen tanks in ICETs are identical to the tanks in FCTs. Consequently, as we aim for a 1,000 km range in all truck configurations and hydrogen consumption per km is expected to be higher for ICETs, compared to FCTs (see section 5.1), more tanks are needed for ICETs. Onboard hydrogen storage for FCTs at 700 bar is estimated to be 80 kg while for ICETs 92.8 kg are necessary. For 700 bar storage 6 tanks (rounded from 5.8 tanks) are required while for 350 Bar, 8 tanks were estimated (rounded from 8.1). The BoP for both ICET versions (350 bar and 700 bar) was assumed to have the same material composition as their FCT counterparts.

Pipes and valves in the 700 bar BoP are expected to be thicker compared to the 350 bar BoP, as they withstand larger pressures. In contrast, the 350 bar system is expected to connect a larger number of tanks, requiring more material for pipes and valves. Thereby, the exact mass or material composition of both BoP is influenced by design parameters, which are beyond the scope of this study. For the sake of simplicity and expecting a low environmental footprint from the BoP, for all impact categories, we kept identical BoP for all vehicle versions.

Regarding the carbon fiber production data, Weiszflog & Abbas [22] appealed to data in Benitez et al.[6], which was assumed to be produced in Germany. Benitez et al. [6] include exhaust gas treatment for the stabilization, in addition to low and high temperature carbonization. All reactions were assumed to be stoichiometric.

2.2.4. Fuel cell system

The fuel cell weight is based in the Powercell MS-100 fuel cell system (FCS) [24]. Overall, the FCS comprises of the fuel cell stack and the auxiliary components for hydrogen management, air management, thermal management and water management systems [54]. The MS-100 system contains the following components: fuel cell stack, control system, cathode air subsystem, cooling subsystem, and hydrogen subsystems. Total weight is 212 kg.

The BoP of the fuel cell should not be confused with the BoP of the H_2 tank system depicted by Weiszflog & Abbas [22], which incorporates valves, pipes, and instruments required for transporting the hydrogen to the fuel cell. In fact, Weiszflog & Abbas [22] indicate that their data does not contain any auxiliary components within the FCS. Since it is unclear which exact components are included in the hydrogen tank BoP, we might be counting

some components twice in the hydrogen management system or in the cooling system. Nonetheless, in case of double counting, it would likely refer to ducts or valves, which are expected to have little environmental footprint impact. The air filter for the cathode system was not included due to lack of data.

As the specific inputs for the fuel cell stack in Franz & Liljenroth [23] were not disclosed due to confidentiality agreements, we appealed to GREET [27] for material composition data. The platinum content was added based on the estimations for heavy duty vehicles in WPIC [55]: 0.53 g Pt kW^{-1} in 2022 but platinum use reduction is forecast to reach 0.25 g Pt kW^{-1} driven by the efforts to reduce platinum loadings [56]. Based on the Powercell MS-100 FCS we estimated a power delivery per kg of fuel cell of 0.47 kW kg FC^{-1} which results in approximately 0.25 g Pt $kgFC^{-1}$.

Table 11. Bill of materials per kg of fuel cell system, according to GREET. Platinum inputs are our own estimation.

**Input**	**%**	**Provider**
Wrought aluminum	5.7	market for aluminium| aluminium, wrought alloy GLO
Cast aluminum	7.4	market for aluminium | aluminium, cast alloy |GLO
Carbon	1.6	market for carbon black | carbon black GLO
Carbon paper	1.1	market for carbon black | carbon black GLO
Iron	0.2	market for cast iron | cast iron | GLO
Chromium	0.1	chromium production | chromium | RER
Copper	2	market for copper cake | copper cake |GLO
Glass fiber	3.8	glass fibre production | glass fibre | RER
Nickel	0.3	market for nickel, class 1 | nickel, class 1 | GLO
Nylon	0.1	market for nylon 6, glass-filled | nylon 6, glass-filled |RER
Platinum	0.25g/kgFC	market for platinum | platinum | GLO
PET	2.9	polyethylene terephthalate, granulate, amorphous |RER
Plastic	2.1	polypropylene production, granulate | RER
Polypropylene	10	polypropylene production, granulate | RER
PSS + others	2.2	market for polyphenylene sulfide | GLO
Stainless steel	52.3	market for steel, chromium steel 18/8 | GLO
Steel	5.7	market for steel, low-alloyed | GLO
Rubber	0.9	synthetic rubber production | synthetic rubber | RER
PTFE	0.4	tetrafluoroethylene production | tetrafluoroethylene | RER
PFSA	0.8	market for alkyl sulphate (C12-14) | alkyl sulphate (C12-14) GLO

2.2.5.Batteries

LiB in FCTs serve as buffer to manage peaks in propulsion energy demand, reducing the fuel cell dynamics, increasing its lifetime and enabling brake energy recovery [8], while facilitating operational efficiency optimization [53]. It is unclear what specific type of LiB chemistry is going to be deployed in FCTs by the truck manufacturers. Still, the fact that LiB technology for electric buses is dominated by nickel manganese cobalt (NMC), lithium titanium oxide (LTO), and lithium iron phosphate (LFP) [38], might suggest these are likely candidates to appear in heavy-duty applications.

NMC offers the highest energy density, resulting in the lightest of the three alternatives, while offering the highest weight-to-storage capacity ratio and also increasing the potential for regenerative braking. In contrast, NMC has the shortest cycle life of the three alternatives, a higher cost due to its cobalt use [57–59] and concerns due to raw material availability [60–62]. Moreover, LTO has the highest cycle life, but also the lowest energy density, implying heavier batteries, compared to LFP and NMC [38]. Besides, low capacity LTO batteries might not be able to take full advantage of regenerative braking. Finally, LFP features fall between LTO and NMC in many aspects [38].

The energy density variation between NMC, LTO and LFP, along with the different LiB capacities in FCT_{200} and FCT_{300}, suggests that different truck configurations might use different battery chemistries depending on different customer needs. FCT_{200} & FCT_{300} represent two opposite approaches to address peak power

dynamics. The first relies more on the fuel cell capacity while the latter is more reliant on the LiB capacity. Thus, the lower battery capacity in FCT_{300} seems to fit well for NMC's higher energy density while also offering the highest regenerative braking capacities.

However, energy density might not be the major driver for battery design in FCT_{200} as the energy storage is not the LiB itself, but the hydrogen stored in the tanks. This suggests that the battery in FCT_{200} is likely to be dedicated for power density, instead of energy density. Caused by physical constraints, LiBs involve a trade-off between energy density and power density; basically, a high-power output requires thin battery electrodes for a fast response, whereas high energy storage is linked to thicker plates. Besides, battery cells can adopt pouch or cylindrical shapes, where cylindrical shapes usually have higher power density [63].

Due to the lack of data on the specific LiB employed, we decided to adopt the pouch NMC battery data as in Ellingsen et al. [38] for all truck versions. The material composition data was scaled to represent a 140-kWh battery for FCT_{200} and a 40-kWh battery for FCT_{300}. For the active material we took data from Chordia et al. [63] representing an NMC-811, instead of the original NMC-622 included in Ellingsen et al. [38]. We acknowledge a mismatch in this adaptation as the active material in Chordia et al. [63] is valid for a density range of 210-240 Wh kg^{-1} while Ellingsen et al. [38] refers to a 240 Wh kg^{-1} cell. In any case, we expect the active cathode material to use progressively less cobalt to tackle supply unavailability and high costs. On the anode side, conventional graphite was kept as in Ellingsen et al. [38].

Battery cell manufacturing was assumed to take place in China, including the cathode active materials. LCA data for the electricity was taken as in Ecoinvent v3.8 process "*market group for electricity, medium voltage | electricity, medium voltage | CN*". However, we assumed the battery packs to be transported and then assembled in Belgium. This choice was inspired on recently built battery manufacturing facilities near Ghent, by Truck manufacturer Volvo [64].

Finally, lead-acid batteries are included in all vehicles to provide power for auxiliaries. We adopted the mass and material composition as in GREET [27]. Table 12 depicts the BoM and the Ecoinvent v3.8 processes used for modelling.

Table 12. Bill of materials for the lead-acid batteries and Ecoinvent processes used for modelling.

**Input**	**%**	**Provider**
Plastic	6.1	polypropylene production, granulate | polypropylene, granulate | RER
Lead	69	market for lead | lead | GLO
Sulfuric acid	7.9	market for sulfuric acid | sulfuric acid | RER
Fiberglass	2.1	glass fibre production | glass fibre | RER
Water	14	market group for tap water | tap water | RER
Others	1	glass fibre production | glass fibre | RER

2.2.6. Exhaust After-treatment

In contrast to diesel combustion, hydrogen combustion produces only small amounts of unburnt hydrocarbons (linked to the engine lubricants of the pilot fuel), and carbon monoxide [65,66]. Therefore, the system design is expected to vary compared to diesel trucks. For instance, oxidation catalysts, used for oxidizing hydrocarbons and carbon monoxide into carbon dioxide, but also for oxidizing nitric oxides to nitrogen dioxide, are likely unnecessary for treating exhaust gasses [65,66]. Diesel particle filters are therefore also likely unnecessary. The major design driver of the selective catalytic reduction (SCR) system for hydrogen trucks is the treatment of NOx emissions. The specifics of the after-treatment system are also expected to vary between regions [65].

Due to the uncertainties, we assumed SCR as the chosen technology for treatment. By assuming no catalyst oxidation systems are necessary, the need for platinum catalyst is eliminated. Nonetheless, the SCR system will require catalysts. Research has assessed the use of copper or vanadium as candidates [67].

System weight was taken from JRC [8] while the material composition was based on Ecoinvent dataset "*catalytic converter production, selective catalytic reduction, 200 liter* " whose catalyst is titanium dioxide.

2.2.7. Trailer

Trailer total mass was assumed as in JRC [8]. The material composition was taken from the Class 8 sleeper cab truck in GREET model [27] and comprises of trailer body, trailer chassis, and trailer auxiliaries.

2.2.8. Cooling, air and water management system

The total mass and material composition of the cooling system for FCTs and ICETs was established based on discussions with experts[35] and our own assumptions. The heat to be dissipated from the FCS originates from the fuel cell stack, which works at a lower temperature when compared to the ICE working temperature. Consequently, the FCS cooling system will likely require larger radiators. The cooling system will likely consist of a low temperature circuit for cooling the compressor discharge air and a high temperature circuit for the stack coolant[54]. Despite the differences, the material composition for compressors, radiators, hoses, fans, pipes, valves, and instruments is not expected to vary radically.

Moreover, the water management system is in charge of recirculating the hydrogen stream but also in charge of ensuring longer membrane lifetimes by avoiding hot dry operation by including a humidifier. In contrast, the air management system includes an air compressor to guarantee enough supply to the cathode side in addition to an air filter. Electronics for control are also required but the exact material composition will strongly depend on the specifics of the system design.

In sight of this, but also driven by the lack of specific data, we assumed the material composition displayed in Table 13. Moreover, the larger fuel cell found in FCT_{300} will likely need a more robust cooling system compared to FCT_{200}; thus, the cooling system in the FCT_{300} is heavier than in the FCT_{200}. We are aware the MS-100 data [24] includes the cooling system elements associated with the FCS, but such data does not contain the external cooling module, which is expected to contain heavy devices *e.g.* radiators, compressors, evaporators. The material composition for the ICET cooling system was considered to be similar but weight is expected to be lower.

Table 13. Bill of materials for the cooling, air, and water management system and Ecoinvent processes used for modelling.

Input	%	Provider
Cast Aluminum	30	market for aluminium, cast alloy | aluminium, cast alloy GLO
Steel, stainless	25	market for steel, chromium steel 18/8 | steel, chromium steel 18/8 GLO
Rubber	20	synthetic rubber production | synthetic rubber RER
Steel	10	market for steel, low-alloyed | steel, low-alloyed GLO
Electronics	5	electronics production, for control units | electronics, for control units | RER
Plastics	10	polypropylene production, granulate | polypropylene, granulate RER

2.2.9. Assembly

Data for energy assembly was taken from GREET [27] and it represents the energy required for the assembly of the chassis and body. Included inputs are the electricity consumption for air compression, welding, material handling, HVAC & lighting, and paint production. Heating for vehicle assembly and painting was also included.

3. Hydrogen production

3.1. Green hydrogen

Electrolysis splits water into hydrogen and oxygen by using electricity. Currently, alkaline electrolysis (AE) is the most mature and commonly used technology [40,68,69]. By middle of this decade, around 60% of the total installed capacity is still expected to be AE; however, a shift towards polymer electrolyte membrane (PEM) electrolyzers is expected, so by 2030, the total capacity could be split 50/50 between AE and PEM [70].

Although PEM electrolyzers have been mostly used in small scale installations, it has potential for expansion, especially as the dynamic power response of PEM electrolyzers is more flexible than in AE [68,69,71], rendering PEM technology particularly suited for facilities backed by intermittent electricity supply as in the case of wind or solar power [69,72,73]. In addition, due to the modular nature of some PEM electrolyzers, the capacity can be scaled up or down according to the specific requirements of each application [74], as already seen commercially [75].

Furthermore, the presence of impurities in hydrogen, with potential to deactivate the catalyst and degrade the fuel cells, such as carbon monoxide or hydrogen sulfide [76,77], is exceedingly rare in PEM electrolysis. This is advantageous as automotive fuel cells require hydrogen to be 99.97% pure [77]. The presence of oxygen, water and nitrogen compounds, found in the air used for the reaction, can be removed via purification [78].

On the downside, PEM electrolyzers need expensive, and scarce, precious metals, namely platinum and iridium. In fact, around 12 % of the electrolyzer costs, at 125 USD kW^{-1} in 2022, were linked to metals, including steel, aluminum and titanium, but still largely linked to platinum and iridium costs [70]. However, efforts are being made to reduce the material needs of platinum group metals [70] and iridium-free PEM electrolyzers are under development [79]. Electrolysis costs are expected to decline based on the reduction of Capex costs, the decline in the levelized cost of energy and increasing utilization levels [40].

Platinum and iridium loadings were originally included in Bekel et al. [5] as 0.8 mg $cm^{2\ -1}$ and 1.5 mg $cm^{2\ -1}$, respectively. To account for material reduction up to 2030, iridium ink loading was adjusted to 0.9 mg $cm^{2\ -1}$, as more recently estimated by Gulotta et al. [80], while maintaining the same plate dimensions (70 x 40 cm) and number of plates (167) as in Bekel et al.[5]. Indeed, pure iridium loading should be even lower than 0.9 mg $cm^{2\ -1}$ since the ink applied to the anode is in the form of Iridium-Ruthenium-Oxide ($IrRuO_2$) [80] which contains 54% of iridium, 28% of ruthenium while the rest is oxygen, according to their molar masses. Despite $IrRuO_2$ representing a small share of the fuel cell stack, Gulotta et al. [80] estimated it as less than 0.02% of the stack's total mass, the characterization factor of iridium is the largest in the crustal scarcity indicator (CSI) method [81]. In addition, we identified a lack of LCIs representing iridium in Ecoinvent or in literature. Thereby, we employed the Rhodium datasets in Ecoinvent as a proxy, only adapting its CSI characterization factor to match that of iridium $7.6E^9$ kgSieq,

This is a rough approximation and is a limitation for this study. Nonetheless, an in-depth analysis for the platinum group metals in the fuel cell stack aiming for 2030, or the construction of LCIs for iridium production is beyond the scope of this research. For Nafion we maintained the film thickness as 180 μm.

All in all, the choice of PEM electrolyzers for this study recognizes their quick dynamic power response and also considers that falling prices will make it attractive enough for attending the heavy-duty market. However, AE presents potential as well, especially for cases where electrolysis is powered by the grid.

3.1.1. Electrolyzer performance

We included four cases of electrolytic hydrogen production, two for centralized and two for distributed (also known as "on-site") production. More specifically, we include two cases of electrolysis 100% powered by wind energy, also known as green hydrogen (GH_2) and two cases in which the electrolyzer is connected to the electricity grid (SgH_2) as illustrated in Figure 4. Aiming for a clearer picture of the performance parameters influencing the operation of PEM electrolyzers, a literature review for both central and distributed production facilities was performed, as seen in Table *14*.

Table 14. Literature review results for the technical parameters associated to PEM electrolytic production of hydrogen and values for this study.

	Functional unit	Time scope	Capacity (MW)	Electricity Consumption (kWh KgH_2^{-1})	Water Consumption (Kg KgH_2^{-1})	Average flow rate (KgH_2 day^{-1})	Capacity factor (%)	Stack lifespan (yr)	Purity (%)
Distributed production									
Wulf & Kaltschmitt [73]	1 Kg H_2	2032	-	50	19	526.03	0.457[(1)]	8 years	-
Bekel & Pauliuk [5]	1 km [(2)]	-	1.42	63	11.2	350.68	0.65	60,000	-
H_2a 2014-case. NREL [82]	1 Kg H_2@450psi	2014	3.94	55.8	13.2	1,500	0.86	52,735	99.6
H_2a future case. NREL [82]	1 Kg H_2@450psi	Future (unspecified)	3.8	51.4	14.3	1,500	0.86	75,336	99.6
HyLYZER [75]	1 Kg H_2	2021	25	55.5-59.9	<15.5	449.5	N/A	-	99.998
KPI[(4)]. IRENA [68]	-	2020	-	50-83	-	-	-	50,000 - 80,000	99.9-99.9999
KPI[(4)] targets . IRENA [68]	-	2050	-	<45	-	-	-	100,000-120,000	99.9-99.9999
This study	1KgH_2@1bar	2020-2030	5.09	60	15.5	See details[(6)]	See details[(6)]	60,000	99.97[(5)]
Centralized production									
Delpierre et al [72] "w/o wind power scenario"[(3)]	1 Kg H_2@20bar	2050	100	50	10	-	-	80,000	Not disclosed
Delpierre et al [72] "Full H2 power scenario"[(3)]	1 Kg H_2@20bar	2050	1,000	50	9	-	-	130,000	Not disclosed
H_2a 2014 case. NREL [82]	1 Kg H_2@450psi	2014	130.7	55.5	14.3	50,000	0.97	59,480	99.6
H_2a future case. NREL [82]	1 Kg H_2@450psi	Future (unspecified)	126.4	51.3	14.3	50,000	0.97	84,972	99.6
This study	1KgH_2@1bar	2020-2030	500	55.5	14.3	See details[(6)]	See details[(6)]	80,000	99.97[(5)]

(1) 100% fed by renewables

(2) Functional unit 1 km travelled

(3) LCIs in Delpierre et al [72] is based on adaptations to Wulf & Kaltschmitt [73]

(4) KPI: Key performance indicators

(5) Considered as High Purity Grade. ISO 14687 e2:2012 states vehicle fuel cells require a purity of at least 99.97%

(6) Table 15 displays the information for each one of the 4 evaluated configurations.

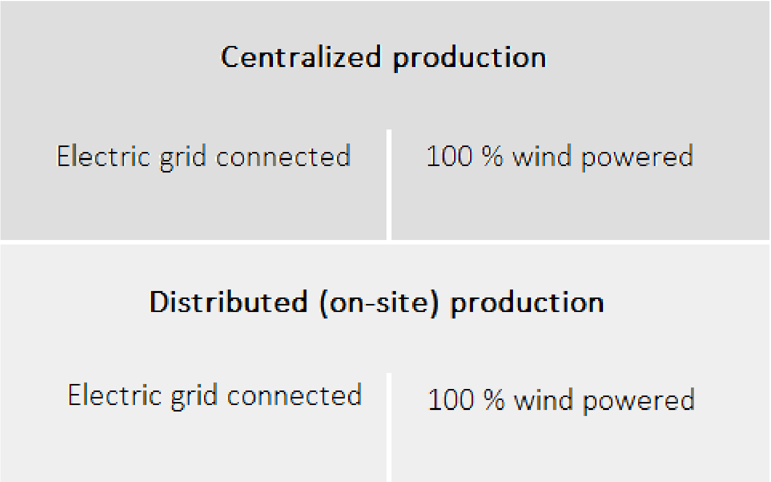

Figure 4.Four cases of electrolytic hydrogen evaluated.

The electrolyzer capacity (MW) defines both the hydrogen flow rate and the amount of materials required for the electrolyzer itself. Larger hydrogen flow rates not only imply larger electrolyzer stacks, but also more auxiliary pipes, valves and other instruments required for moving the gaseous hydrogen along the electrolyzer, this set of instruments is known as the balance of plant (BoP) of the electrolyzer. The hydrogen output is a function of the electrolyzer capacity (MW), efficiency (%), and the capacity factor (%) which measures the overall utilization of the hydrogen production infrastructure over a given period.

We established the hydrogen production rate as a design constraint, meaning that, independently from the source of the electricity being fed to the electrolyzer, the stack capacity, or the capacity factor, the on-site PEM electrolyzer must produce 2 t H_2 day^{-1} or around 83.3 kg hr^{-1}. On the other hand, the mean flow rate from the centralized production plants will be around 8,823 kg hr^{-1}. Table 15 displays the modelling parameters for all cases of electrolytic hydrogen.

Table 15. Modelling parameters for centralized and distributed (on-site) production of electrolytic hydrogen.

Parameter	On site-grid	On site-wind	Central-grid	Central-wind
Mean Hour production (kg hr^{-1})	83.3	83.3	8,823.5	8,823.5
Stack lifetime (hr)	60000	60000	80,000	80,000
Electricity (kWh kg^{-1})	60.0	60.0	55.5	55.5
Capacity factor (%)	0.9	0.46	0.9	0.46
Hourly production given capacity factor (kg h^{-1})	92.6	182.5	9,803.9	19,323.5
Plant capacity (kw)	5,092.6	10,037.5	500,000	985,500
Max hourly production (kg hr^{-1})	92.6	182.5	9009.9	17,756.8
Production during stack lifetime (t)	5,555.6	10,950	720,720.7	1,420,540.5
Mean yearly production (t)	730	730	71027.0	71027.0
Lifetime stack (yr)	7.6	15	10.1	20
System lifespan (yr)	20	20	20	20
Stacks along lifespan (number)	2.6	1.3	2.0	1.0
Excess of stack (%)	13	13	13	13
Total number of stacks (number)	2.97	1.51	2.23	1.13
Total production along lifetime (t)	14,600	14,600	1,420,540.5	1,420,540.5
Electrolyzer used per kg H_2 (amount)	3.81E-07	7.50E-07	3.52E-07	6.94E-07
System efficiency (%)	54.6	54.6	59.0	59.0
Minimum required daily production (kg)	2,000	2,000	8,823.5	8,823.5

As the capacity factor of a PEM electrolyzer linked to a wind farm is lower than the capacity factor of a grid-connected one, but the required mean hourly production is the same, the wind-powered PEM electrolyzer would need a larger capacity to work at full power during the renewable electricity surplus hours. In other words, to achieve the required throughput, a larger electrolyzer and storage facilities will be required when producing GH_2. Such storage infrastructure, in this study, is assumed to be spherical pressure vessels for CH_2 as described by Papadias et al.[83] and cryogenic spherical vessels for LH_2 as currently found in NASA facilities [84]. Specifics regarding the storage facilities can be found in Section 4.

3.1.2. Centralized production

The production capacity proposed for the centralized production facilities (500 MW) is in consonance with IRENA's projections for electrolyzer deployment in the medium term, more specifically, Stage 2 of electrolyzer deployment [68]. Stage 2 assumes that electrolyzer development has reached a point where the largest players are benefiting from economies of scale (60%-70% stack cost reduction in MW, compared to 2019), reaching the 1 GW $year^{-1}$ milestone. Thus, our proposed production plant capacity (0.5 GW) is reasonable. Furthermore, electricity consumption was assumed as 55.5 kWh kgH_2^{-1}, at the system level, as in NREL [82], which is also in line with IRENA [68], which states a 50-83 kWh kgH_2^{-1} range.

For the wind-powered centralized plant we considered an offshore wind farm installation along the western Swedish shores. In contrast, for distributed production of hydrogen we assumed the electricity to come from a smaller onshore farm. The electrolyzers were assumed to be manufactured in Germany and display an efficiency of 59.0% (lower heating value); another conservative assumption for PEM electrolyzers considering the potential gains due to economies of scale [68].

For grid-connected electrolysis we assumed a 90% capacity factor while for wind-powered electrolysis a capacity factor of 45.7% was adopted as in Wulf & Kaltschmitt [73]. This is relevant, as lower capacity factors, and hence less yearly functioning time, was assumed to extend the lifetime of fuel-cell stacks, based on the simplification that frequent on/off cycles will not negatively affect the fuel-cell stack lifetime. To more realistically estimate the number of fuel-cell stacks required during the entire lifetime of the electrolyzer we included 13% of required excess stack, as in H2A current scenarios defined by NREL [82]. The stack excess aims to compensate for eventual stack degradation which diminishes the flow rate of production.

The specific life cycle inventories (LCI) for the electrolyzer were based on Bekel & Pauliuk [5].

3.1.3. Distributed production

For on-site production we adopted a minimum production capacity of 2 t H_2 day^{-1}. Currently, this rate of production could be achieved by installing several module-sized PEM electrolyzers. for instance, the Silyzer 300 consists of 24 PEM electrolytic modules that together draw 17.4 MW of power to produce up to 730 pounds (330 kg) an hour of high purity grade hydrogen with no CO_2 emissions [85,86]. Moreover, the HyLYZER-5000-30 produces around 5,000 Nm^3 hr^{-1} or about 450 kg hr^{-1} with a 25 MW electrolyzer [75]. Analogously to the case of centralized production, this study assumes that within our time frame (2020-2030), IRENA's Stage 2 of electrolyzer deployment would be reached [68], meaning that 20 MW-100 MW modules will be usual in the next 10 years. The module-sized electrolyzer is assumed to be produced in Germany. Electricity consumption was taken as 60 kWh kgH_2^{-1} at the system level, which matches the performance for commercially available PEM electrolyzers [75], at a system efficiency of 54.6%. Lower than that of centralized production.

Regarding the life expectancy, IRENA [68] reports lifetimes of more than 50,000 hours in PEM fuel-cell stacks. Likewise, the H2a program assumes 59,480 hours lifetime in their calculations [82] while Bekel & Pauliuk [5] consider 60,000 hours. All in all, while there is some variability in the reported electrolyzer lifetime, there is general agreement that current PEM systems present lifetimes of approximately 60,000 hours [87].

3.2. Blue hydrogen

Blue hydrogen (BH_2) is praised for its potential synergy with existing NG infrastructures [49,88,89] considering that CO_2 underground injection technologies are mature [90] and exhibit a long history of applications, mostly for enhanced oil recovery (EOR) [91–93]. As 83% of the world hydrogen in 2022 came from fossil sources [79], adding an extra carbon capture and storage (CCS) stage to existing SMR processes could potentially decarbonize the hydrogen production in already existing production facilities. Currently, BH_2 is cheaper than GH_2 [68,93], although estimations suggest that price parity could come as early as 2030, driven by larger electrolysis plants and decreasing costs in renewable electricity [68,89]. In contrast, analysts argue price parity could take at least a

decade to arrive [94]. Hence, including NG-based hydrogen production in European decarbonization plans could result in considerable savings; Deloitte Finance [95] estimated those savings in €2 trillion by 2050.

The SMR process exhibits several stages, where severe operating conditions are found. Conversion of methane is complete at temperatures over 700 °C [78] or even 900 °C, depending on operation parameters, such as pressure and steam excess [96]. Firstly, the fuel is processed into a reactor by adding air -or oxygen- and steam to produce a blend consisting mainly of hydrogen and CO, also known as synthesis gas or syngas [97]. Steam reforming is basically an endothermic conversion of hydrocarbons [98] taking place in hundreds of parallel, externally heated tubes, in presence of a nickel catalyst [78], where steam is fed in excess to reduce coke formation [78,96]. Heat is recovered and recycled back to the reactor to diminish fuel consumption [96,99,100]. The steam reforming reaction for an arbitrary hydrocarbon is written in Equation 1.

$$C_xH_y + x \cdot H_2O \rightarrow \left(\frac{y}{2} + x\right) H_2 + x \cdot CO \qquad \text{Equation 1}$$

In the second stage, more hydrogen and CO_2 are produced by reacting CO with steam in a water gas-shift (WGS) reaction. The WGS is aimed at producing a higher hydrogen yield [96] and is often divided in a high temperature stage, containing iron and chrome catalysts, and in a low temperature stage, which works on zinc and copper catalysts [78]. The high concentrations of CO_2 in the syngas leaving the WGS reactor- between 15 and 60% by volume on a dry basis- and the high pressures involved are auspicious for CO_2 separation. At this point, the hydrogen and CO_2 present in the syngas can be separated. If the CO_2 is removed and stored, the hydrogen can be considered a carbon-free energy carrier. Around 85-95% of the CO_2 in the syngas is usually captured by the system [99] resulting in an overall capture rate of about 80%. The WGS reaction related to SMR is shown in Equation 2.

$$CO + H_2O \rightarrow CO_2 + H_2 \qquad \text{Equation 2}$$

Hydrogen produced in current industrial complexes is mostly intended for oil-refining and nitrogen fertilizer production, which do not require high levels of purity, while little is dedicated for energy due to its high costs compared to fossil fuels [100]. To be suitable for use in fuel cells, hydrogen requires 99.97 % purity [77]. Thus, a purification stage is required, since hydrogen leaving the WGS reactor still contains impurities, harmful for fuel cells [73,96]. Purification is typically carried out via pressure swing adsorption (PSA) [78,96,99,101], and is based on the physical binding of gas molecules to adsorbent material which can be zeolites, silica or activated carbons[101]. PSA is commonly used since it has low capital costs and high reliability [93], which can result in purities of up to 99.999% [97,101].

Although PSA produces very pure hydrogen it does not separate the CO_2 from the stream of gases that remains after the hydrogen is removed. Consequently, this stream of gases coming out from the PSA unit contains methane, CO_2, CO, and traces of H_2, which is then mixed with NG and recirculated as fuel in the reforming reactor [99,102]. In fact, SMR plants usually produce energy in excess, driven by the combustion of the feedstock, of that required for the reforming process, which can be used to generate electricity [103]. However, this situation depends on the specific configuration of the process, for instance, when CCS is added, extra electricity or heat is typically required to run the process [104], instead of exported, changing the balance of inputs and outputs. From an economic perspective, researchers have estimated that, when CCS is added, costs increase [105,106].

The LCIs in this study were obtained from Antonini et al. [99], which evaluated cases of SMR, including and not including CCS, and found that SMR without CCS was the only configuration not requiring any electricity consumption from the grid. Nonetheless, for cases including CCS, the electricity requirements were relatively small per m^3 of hydrogen produced. Based on the same data, Bauer et al. [103] reached similar conclusions. Such findings are in consonance with IEA [70].

Moreover, when specifically referring to the efficiency of the SMR process itself, and not the total plant efficiency, the cases where CCS was added to conventional SMR, had a larger efficiency than the cases of pure SMR, without adding CCS [99]. This counterintuitive outcome is the result of the variations in the recirculated tail gas burnt in the reformer furnace: the more CO_2 that is captured from the syngas, the less CO_2 that will be present in the tail gas, therefore less CO_2 is present in the mix of fuel and tail gas coming into the SMR reactor. Consequently, the heating value of the tail gas will be higher, and the furnace will require less additional fuel [99]. Thereby, efficiency gains can arise because of adjustments in each specific plant. Two decades ago, CO_2

capture, and its subsequent compression, could increase the energy needs around 10–40% compared to plants without CO_2 capture [97], current increases are lower than that [99,103].

Unavoidably, some CO_2 is emitted during BH_2 production [100] whereas some methane is emitted unburned to the atmosphere during NG extraction and transportation, caused by leaks or intentional venting [100]. The GHG emissions from the BH_2 production process arise from three sources: 1) the SMR process itself, where methane is transformed into CO_2 and hydrogen; 2) the energy used to generate the heat and high pressure needed to drive the process, which generally comes from natural gas [100]; and 3) energy needed to run the CO_2 capture equipment [78,100,103]. The results of the literature review are presented in Table 16. It includes an additional configuration, where the CO_2 in the flue gases is captured. However, in this study we do not consider the capture of the flue gases.

Even though we strived to present harmonized results from the literature review, most of the studies are not fully comparable, due to differences in the system boundaries. Indeed, in some cases the studies failed to explicitly identify the system boundaries, making a direct comparison unfeasible.

For instance, the system boundaries in IEAGHG [104] include the production of demineralized water while in Antonini et al. [99] and in NREL [82] such inputs are placed outside the boundaries. Wulf & Kaltschmitt [73] do not specify the origin of demineralized water at all. Only the study by Antonini et al.[99] could be strictly classified as an LCA study. This problem with harmonization in LCA studies applied to hydrogen was already discussed by Valente et al.[9]. We addressed their recommendations by specifying the production parameters for our study, as seen in Table 16.

Besides methane, NG contains small percentages of CO_2, N_2 and heavier hydrocarbons and even a few ppm of chloride and sulfur which are harmful for the catalysts in the reformer; hence, those compounds need to be removed [101]. The system boundaries in Antonini et al. [99] already include this purification stage.

We performed some adjustments to the LCIs found in Antonini et al.[99]. Firstly, we removed the energy required for compression. The original LCIs included the compression from 26 bar, at the reformer exit, to 200 Bar, which was the storage pressure in tanks. The reason is that we considered the compression not to belong to the production stage. In personal communication with the authors, we were informed that their compressor required 11.2 MWe for compressing 9 t $hour^{-1}$ of hydrogen, which translates into an electricity consumption of 1.25 kWh kg^{-1}. For comparison, a similar requirement for compression, 1.13 kWh kg^{-1}, from 22 to 200 Bar was indicated by Valente et al.[7]

The GWP of BH_2 is estimated to increase as methane leakages increase in the upstream NG supply chain [99,100,103]. Nonetheless, as the NG leakages from extraction in Norwegian gas fields in the North Sea are estimated to be practically inexistent [107], the base value for leakages only included transmission to Sweden, estimated as 0.8%[108]. Aiming to test the sensitivity we proposed a pessimistic case where leakages reach a value of 3.4%; 2.6% from extraction and 0.8% from transmission. Leakages of 3.4% are in line with Ecoinvent V3.8 datasets [108], which derive from data from Norwegian fields in the north sea[109] but is slightly larger than the 2.1% estimated average for offshore leakages on European oil fields[107]. The low presence of leakages in Norwegian fields mean that GWP results are expected to be similar for time-scopes of 20 and 100 years [99,100,103] highlighting instead the importance of the carbon removal technology. We chose the MDEA (methyl diethanolamine) technology with a 90% removal rate from the syngas, which represents a mature technology and a realistic performance [99].

Table 16. Literature review results for the technical parameters associated to blue hydrogen and grey hydrogen.

	Functional unit	Time scope	Flow rate (KgH$_2$ hr^{-1})	Natural gas consumption (MJ KgH$_2^{-1}$)	Electricity consumption (kWh KgH$_2^{-1}$)	Water consumption (Kg KgH$_2^{-1}$)	Capacity factor (%)	Plant lifespan (yr)	Purity (%)	Carbon Capture rate(%)	Emitted CO_2 (g MJ^{-1})	Emitted CH4 (g MJ^{-1})	Coproducts
Grey hydrogen cases (NO CCS)													
Wulf & Kaltschmidt [73(1)]	1 Kg H_2	2032	48	158	0.2	14.4	0.86		-	-	71.26	-	5.3 Kg Steam KgH$_2^{-1}$
IEAGHG [104(2)]	100,000 Nm^{3}H$_2$	2015	8,994.0	158.08	-	6.64[(3)]	0.7 & 0.95[(4)]	25	>99.5	-	75.01	-	9,918 MW[(5)]
Howarth & Jacobson [100(6)]	1 MJ H_2	-	-	142.83[(7)]	-	-	-	-	-	-	70.30	0.90	-
NREL [82], H2a central SMR[(8)]	1 Kg H_2 @450psi	2015	18,112.5	165.0	0.57	3.36	0.9	-	99.6	-	77.37	-	-
Mehmeti et al [110]	1 Kg H_2	-	-	165.0	1.11	21.87	-	-	-	-	-	-	-
Antonini et al [99 (9)]	1 MJ H_2 @200 bar [(10)]	-	8,133.6	182.08	0.01	7.54	0.95	25.00	>99.97	-	74.35	5.11E-4	
Blue hydrogen cases, NG as feedstock (CCS in the syngas stream using Methyl diethanolamine (MDEA)													
IEAGHG [104(2)]	100,000 Nm^{3}H$_2$	2015	8,994.0	163.26[(8)]	-	6.80	0.7 & 0.95[(4)]	25	>99.5	55.7	34.34	-	1,492 MW[(5)]
Howarth & Jacobson [100(1)]	1 MJ H_2	-	-	159.57	-	-	-	-	-	85	45.80	1.01	-
NREL [82], H2a central SMR	1 Kg H_2 @450psi	2015	14,227.0	177.0	1.50	8.13	0.9	-	99.6	96.3	3.08	0	-
Antonini et al [99(11)]	1 MJ H_2 @200 bar [(9)]	-	8,133.6	179.16	-0.77	7.54	0.95	25.00	>99.97	90.0	21.75	4.67E-4	0.77 kWh KgH$_2^{-1}$ [(15)]
This study[(12)] NG as feedstock	1 MJ H_2@26bar[(13)]	2020-2030	8,133.6	179.16[(14)]	-0.77[(15)]	7.53	0.95	25.00	>99.97	90.0	25.99	4.70E-4	0.77 kWh KgH$_2^{-1}$ [(15)]
This study[(12)] BioCH$_4$ as feedstock	1 MJ H_2@26bar	2020-2030	8,133.6	230.84[(16)]	-0.70[(15)]	7.53	0.95	25.00	>99.97	90.0	0	4.82E-4	0.70 kWh KgH$_2^{-1}$ [(15)]

Blue hydrogen (CCS from syngas using MDEA and flue gas capture)													
Howarth & Jacobson [100](1)	1 MJ H_2	-	-	176.31	-	-	-	-	-	85 & 65 (17)	32.90	1.11	-

(1) Energy for WGS included in the NG consumption. Characteristics unspecified.
(2) Energy required for demineralization of water included in NG inputs. Only high temperature stage in the WGS reactor.
(3) Cooling water not included.
(4) 0.7 during year one and 0.95 onwards.
(5) Time lapse for electricity surplus is not specified.
(6) Not a proper LCA study, but a stoichiometric approach for determining emissions.
(7) Methane consumption is 14 gCH_4 MJ^{-1} for SMR process and 11.6 gCH_4 MJ^{-1} . The share of methane on natural gas was not specified.
(8) H2A model is able to work with different inputs. Values are shown for default conditions.
(9) 72 cases were evaluated in this study including NG and Biomethane used as input. WGS reaction includes high and low temperature stages. System boundaries include the electricity required for compression to 200 Bar from 26 Bar.
(10) At room temperature.
(11) Antonini et al [99] evaluated 72 cases via Aspen plus simulation. This specific case refers to SMR of NG, including high and low temperature WGS reaction + CCS (MDEA) with 90% carbon capture (average values).
(12) Both NG and $BioCH_4$ SMR cases for this study are based on Antonini et al[99]. Electricity required for compression from 26 bar to 200 bar was removed.
(13) Output electrolyzer pressure in Antonini et al[99] is 26 bar. is a common output pressure for PEM electrolyzers [40,75]
(14) NG Lower heating value: 46.5 MJ Kg^{-1}
(15) When the electricity associated with compression from 26 bar to 200 bar is withdrawn the result is a net electricity surplus.
(16) $BioCH_4$ Lower heating value: 45.4 MJ Kg^{-1}
(17) 85% in the syngas and 65% in the flue gas

3.2.1. Emissions sources

For grey hydrogen production (conventional SMR of NG without CCS), around 60% of the total CO_2 produced is associated to the SMR process while the remaining 40 % comes from the NG combustion to drive the process [99,102]. In a similar analysis Howarth & Jacobson [100] estimated that, from the direct emissions of grey hydrogen (70.3 $gCO_2\ MJ^{-1}$), 55% were associated to the SMR reaction while 45% were linked to the energy required to drive the process. A similar analysis was performed for BH_2, where the direct emissions reached 45.8 $gCO_2\ MJ^{-1}$, with 13% associated to the SMR reaction, 70% linked to the energy driving the process, and 18% were caused by the energy required for CO_2 capture. However, when indirect emissions, referring to the fugitive methane emissions were considered, the total emissions of BH_2 and grey hydrogen were nearly even, which emphasizes the significance of methane emissions in the environmental footprint of BH_2.

We found no registered attempts of flue gas capturing by commercial facilities[100]. These refer to the tailpipe gases created from burning fuel to supply the energy needed to drive the CO_2 capture process. Here, the capture would take place after the combustion and not before the combustion as in the case of capturing CO_2 from the syngas. Capturing CO_2 from the flue gas is harder than it is from the syngas, as the CO_2 is more diluted [101]. In addition, precombustion capture is a more economic option. Howarth & Jacobson[100] estimated that, for a capture efficiency of 65% in the flue gases, the overall emissions turned out to be similar to those of BH_2 production without capture CO2 from the flue gas, hypothesizing about the insignificant environmental advantages of this process.

3.2.2. The CO_2 path

The CCS system consists of three main steps: 1) the capture and separation of CO_2 from other compounds; 2) the compression and subsequent transportation of the captured CO_2 to the permanent sequestration site; and 3) the injection of CO_2 in geological reservoirs. Carbon capture has been identified as the costliest stage of the entire operation exhibiting significant capital investments while operation is energy-demandant [91]. Moreover, to run the CCS equipment, energy is required, which implies exporting less electricity to customers in the case of power plants [91] or producing extra electricity in the case of NG treatment or hydrogen production facilities.

Most of the commercially available systems for capturing CO_2 from syngas are based on absorption via methyl diethylamide (MDEA) [93]. Moreover, the novel vacuum pressure swing adsorption (VPSA) includes hydrogen purification and CO_2 capture in a single cycle, reaching higher CO_2 capture rates and a reduction in process complexity at the expense of having higher electricity requirements [99], however, we found no evidence suggesting that VPSA will be deployed at large scales before 2030, the time scope of this study. We opted for the MDEA technology case representing a state-of-the-art 90% capture rate as modelled by Antonini et al. [99]. Higher rates are possible, but separation devices need to be considerably larger, more energy intensive and costly [99]. In any case, the selection of the capture technology is not expected to have a significant environmental impact as MDEA and VPSA footprints were found to be similar for equivalent CO_2 capture rates [99].

The CO_2 capture process aims at producing a concentrated stream of CO_2 for permanent storage. Except when facilities are located right above the geological storage site, the captured CO_2 must be transported to a storage site [97]. This could be done by pipelines or by a series of trailers transporting the CO_2. Pipeline transport is known to be an attractive method, from a cost perspective, for high flow rates and short distances [111]. Usually, for pipeline transportation, this gaseous CO_2 is typically compressed to around 8 MPa to avoid two-phase flow regimes while also increasing the density of the CO_2, reducing transport costs [97].

Although CO_2 leakages could appear during pipeline transport, they are usually very small [97] and are considered negligible in this study. A bigger concern is related to the presence of moisture in the CO_2, as it is highly corrosive, and should be removed before entering the CO_2 pipeline to avoid the costs of constructing pipelines of corrosion resistant material. Instead, dry CO_2 is not corrosive to pipelines even if it contains hydrogen sulfide, oxygen, or nitrogen oxides [97].

Once the CO_2 has been captured and transported it is subsequently pumped into the ground to be permanently stored. This procedure is common, most of the captured carbon in history (around 80-90%) was used for EOR [92], a technique used to keep the reservoir pressure in conventional gas and oil fields [97]. For the geological

storage of CO_2 there are three formations which are deemed as feasible candidates: 1) depleted oil and gas reservoirs; 2) unrecoverable coal beds; and 3) deep saline formations [97]. However, the geological storage of CO_2 might not be permanent if gradual release or sudden disruptions happen in the reservoir [97].

The geologic storage reservoirs in Europe include hydrocarbon fields and also deep saline aquifers, both of which are suitable for CO_2 storage. [112]. The LCIs in Antonini et al.[99] include the transport (over 200 km via pipeline) and geological storage of CO_2 in a saline aquifer at a depth of 800 m. Fortunately from a Sweden's perspective, most of the geologic storage reservoirs suitable for CO_2 storage in Europe, exist in the northern part of the continent [112], with saline aquifer capacity not far from Gothenburg's shore. Thus, we deemed the modelling conditions in Antonini et al.[99] adequately represent the offshore injection in Swedish or Danish waters, in a radius of 200 km from Gothenburg.

3.2.3. The current landscape

Despite years of investment and research, the production capacity of BH_2 worldwide has not matched the expectations created around it. BH_2 still represents less than 1% of total global production [79], indicating that the scale of technology necessary to make a substantial impact on worldwide emissions is still far out of reach. Detractors claim the financial aid granted by governments would be better spent on renewable electricity generation, pointing at the unmet promises and project overcosts associated to CCS [113]. Others directly accuse the oil industry lobby of deviating funds vital for climate mitigation [114,115], while IEEFA [116] claims that methane leakages, capture rates, and hydrogen leakages have been misrepresented in U.S. public policy including BH_2.

The latest report by the Global CCS Institute found that, globally in 2022, only two BH_2 production facilities were operational, six are in an advanced development stage and twelve are in the early stages of development[117]. Furthermore, IEEFA[92] examined the performance of 13 large CCS projects worldwide, which add up to nearly 55% of the total nominal CCS capacity worldwide, and discovered that 10 out of 13 had either failed or largely underperformed, compared to their planned capacities.

One of the most prominent experiences, is the Quest project, in Alberta, Canada, an endeavor that has captured the most CO_2 among all the CCS projects linked to hydrogen production [92]. However, the hydrogen produced at Quest is utilized for the upgrading of bitumen into synthetic crude oil [93] and therefore is not representative of the facilities evaluated in this study. Reportedly, Quest nearly achieved its yearly targets by capturing 5.38 Mton CO_2 (compared to a target of 5.5 Mton) in a period of 5 years, between 2015 and 2020. Nevertheless, the Quest project reported emissions of 1.16 Mton of CO_2 associated to carbon capture and sequestration, equivalent to around 21% of the total CO_2 captured by the project. Moreover, Quest was the only facility injecting CO_2 in dedicated geologic storage formations instead of using it for EOR [70,117].

The other example of an operational plant is the H_2 production facility, the Valero refinery located in Port Arthur, operated by Air Products [92,117,118]. It was the first commercial scale SMR facility to integrate CCS and became operational in 2013, capturing nearly 1Mt CO_2 per year [93]. The captured CO_2 is moved via pipeline for EOR at a nearby oil field [93].

In contrast, the first fossil-fueled power plant in the U.S., the Petra Nova project in Texas, which aimed at generating electricity and capturing CO_2 for use in EOR, had its operations suspended in 2020 amid plunging oil prices [91,119]. But even before the shutdown, Petra Nova had missed its targets on CO_2 capture, due to technical glitches in the CCS facility and the annex NG plant [120] that caused lengthy shutdowns [113].

Similarly, Chevron's Gorgon LNG project in Australia has fallen short of its expected injection capacity [121]. The Gorgon project is not related to BH_2 production; instead, the captured CO_2 is removed from the NG since it is undesired for subsequent liquefaction or combustion processes. In order to maintain the integrity of the sequestration reservoir where the CO_2 is injected, some of the present underground water needs to be removed. Challenges have been reported regarding the treatment of this extracted water, which needs to be subsequently reinjected into shallower water reservoirs. Thus, the problem is not in the CCS process itself but in the reservoir water treatment [122].

In another setback for the CCS industry, Cleco, the electric utility company, revealed that its project Diamond Vault, consisting of the retrofitting of a Louisiana power plant, to make it compatible with CCS, would reduce

the electricity produced and distributed to consumers by about 30% [123]. Meaning that some of the electricity formerly intended for customers would need to be deviated for powering the CCS process. A review by the U.S. Congressional Research Service [91] has reported this penalty consumption to be nearly 20% of a power plant's capacity, a more optimistic estimate compared to Cleco´s announcements. Additionally, it could increase the plant's water use by 55 % [123].

3.2.4.Feedstocks

3.2.4.1. Natural gas

Methane leakages during production and transport of NG have been identified as a critical GWP hotspot which could jeopardize the entire potential of BH_2 as a low carbon energy carrier [93,100,103], since NG is mostly composed of Methane (around 90%). This study assumes that NG feedstock for hydrogen production comes entirely from Norway. Leakages during distribution were not included as we assumed the transmission pipelines would directly come to the centralized BH_2 plant.

Studies have determined that methane emissions happen across the entire NG supply chain, including extraction and transportation [103,124,125]. Howarth and Jacobson [100] consulted 20 studies containing satellite data from 10 major NG fields in the U.S., and found that, in average, methane upstream emissions reached 3.4% of the total gas produced, including 2.6% emitted at the field level and 0.8% emitted during transport and storage. Other studies based on satellite measurements in the Permian basin in the U.S. estimate leakages to reach at least 9% [126]. So far, there is no consensus around the magnitude of those leakages and the resolution capacity of the satellites might affect the results, in addition, there is a lack of robust bottom-up experimental data on methane emissions outside North America [103].

Leakages are site-specific and have shown a significant spatial and temporal variability depending on the operational equipment, environmental conditions and maintenance practices across the global oil and gas basins. One example of satellite-based measuring is the methane tracker of the IEA [107,124], which provides country-specific methane emissions for NG supply chains, it displays emissions stretching from near-zero, for countries like Norway and Qatar, to over 6% for countries like Libya and Iraq.

Including methane emissions for modelling BH_2 in a specific context requires dataset adjustments. There is large variability in the methane emissions from each NG site and transportation infrastructure, making country-level LCA studies prone to errors [103]. Furthermore, NG is usually extracted as associated gas during crude oil extraction, where other coproducts are also produced. Consequently, the measured emissions are associated with all products, requiring allocation for which data is not always available.

3.2.4.1.1. Leakages estimation

For this study we adjusted the Ecoinvent 3.8 [108] datasets representing the NG extraction in Norway and its subsequent transmission to Sweden via pipeline. Although these datasets reflect outdated extraction and treatment technologies – data from 2012- we deemed it adequate for modeling leakages. The reason is that datasets representing outdated technologies are only expected to increase CO_2 emissions, linked mostly to old combustion technologies, but not to methane emissions, which are the main reason for concern. Aiming to better deal with the uncertainties, we modelled two emission cases: low and high leaks.

The structure of Ecoinvent 3.8 datasets describes the different stages of the supply chain. For instance, the process *"Petroleum and gas production, off-shore – NO"* describes the inputs, outputs, and emissions associated to NG extraction in North Sea's fields. This multioutput process has "natural gas" and "crude oil" as co-products. Under the Unit Process scheme, the extracted NG is then used as input for the process *"Natural gas, high pressure, import from NO -SE"*, which models the pipeline transmission from Norway to Sweden via Denmark. The datasets' structure and estimated leakages are depicted in Figure 5.

The NG leakages were represented as a percentage of the NG produced. However, as the Ecoinvent v3.8 dataset *"Petroleum and gas production, off-shore – NO"* does not present this information we had to estimate the leakages based in the emissions included in the dataset. For this purpose, we appealed to equation 3. This equation included in the dataset's metadata, is originally intended to estimate the emission factor (kg m^3NG^{-1}) for each of the emitted species, including methane.

$$Em = CEm * \left(\frac{PetAll*PetFact+NGAll*NGFact*\rho NG}{PetAll*PetProd+NGAll*NGProd*\rho NG}\right)$$ Equation 3

Where:

Em (kg $m^{3\,-1}$)	¤ Emission to the atmosphere
CEm (kg)	¤ Total emitted of a given compound in the gas & oil field.
ρNG (kg $m^{3\,-1}$)	¤ Density of natural gas = 0.84
NGAll (Number between 0-1)	¤ Allocation factor for emissions linked to natural gas. 0.5 for this case
PetAll (Number between 0-1)	¤ Allocation factor for emissions linked to petroleum. 0.5 for this case
NGProd (m^3)	¤ Natural gas produced in Norway in 2012= 49,620,000,000
PetProd (kg^3)	¤ Petroleum produced in Norway in 2012= 159,590,000,000
PetFact (Either 0 or 1)	
NGFact (Either 0 or 1)	

The parameter *CEm* refers to the total emission of a given compound in the extraction field. *CEm* values were obtained from the environmental report of Norwegian oil and gas production in 2012 [109], which includes emissions from 49 producing fields and 178 exploration and production/injection wells. This report states that cold venting and fugitive emissions from flanges, valves and other equipment are the biggest sources of NG emissions. For the sake of simplicity, the methane emissions in *"Petroleum and gas production, off-shore – NO"* dataset is entirely classified as leakage. Notice that the emissions to the atmosphere (kg $m^{3\,-1}$), estimated by equation 3, match the values in the *"Petroleum and gas production, off-shore – NO"* dataset, as seen in Table 17. Afterwards, Equation 4 was used for estimating the leaks, as a percentage of produced NG. Results are also displayed in Table 17.

By following equation 4, we estimated that the methane emissions, as included in the *"Petroleum and gas production, off-shore – NO"* dataset, which describes NG extraction, are equivalent to 0.024% of the total produced gas (kg CH_4 kg NG^{-1}), indicating near-zero emissions for Norwegian fields, as anticipated by the IEA [124]. As natural gas was assumed to contain 85.6% of methane, we estimated the emissions of natural gas as 0.028% of the total gas produced.

$$\frac{(CEm(CH4)*NGAll(CH4))}{NGProd*\rho NG} = CH4\ leakages\ (\%\ of\ total\ gas\ extracted)$$ Equation 4

Thus, for the base-leaks scenario we considered the estimates in the Ecoinvent 3.8 cutoff datasets, estimated in this study as 0.038%, (see Figure 5), whereas for high leakages, we incorporated a 3.4% of leaks, in line with estimates from Howarth and Jacobson [100].

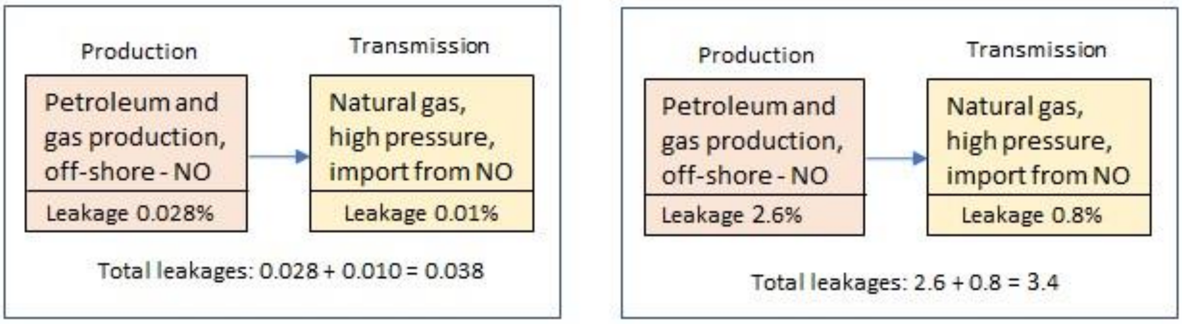

Figure 5. Low (left) and High (right) NG leakage estimations during production and transmission.

When equation 4 is used for CO_2 emissions estimation, results are significantly larger compared to emissions of other compounds. This happens despite the dataset *"Petroleum and gas production, off-shore – NO"* not including the emissions from fuel combustion in turbines, engines, etc., in contrast, it includes well testing (fuel requirements and emissions) and the partial drying of natural gas on the platforms. Since well testing emissions are expected to be small[109] the larger than expected CO_2 emissions could be linked to the partial drying of the natural gas or to flaring. Thus, equation 4 was only considered adequate to determine the approximate leakage of methane in *"Petroleum and gas production, off-shore – NO"* dataset.

In order to represent the case of high leakages for the sensitivity analysis we created a dummy process, based on natural gas composition, representing emissions equivalent to 2.6% of the produced natural gas, see Table 18. All other emissions were kept as in *“Petroleum and gas production, off-shore – NO”* as those substances are not expected to be relevant for the evaluated impact categories, GWP, CSI and PM-EF. The uncertainty in the origin of the CO_2 emissions hampers any attempt to execute sensitivity analyses for CO_2, nonetheless, we found no evidence of CO_2 leakages as a potential threat to the GHG mitigation potential of BH_2.

For the import from Norway (transmission) stage, we used the dataset *“Natural gas, high pressure, import from NO -SE”*. In this case, leakages are indicated as 0.01% by the dataset metadata[108] and were linked to storage and are a function of the gas composition. Furthermore, the ”*Market for natural gas, high pressure - SE“* dataset refers to the local distribution through the Swedish gas grid.

For the sensitivity analysis we linearly adjusted the process emissions in the aforementioned datasets to match the leakage values (as a percentage of total gas produced) indicated by Howarth & Jacobsen [100]; 2.6% for production and 0.8% for transmission respectively.

Table 17. Verification of leakages in the "Petroleum and gas production, off-shore – NO" dataset

	Content (kg Nm^{3-1})	Composition fraction (%)	CEm (kg)	PetAll	NGAll	Emission to the atmosphere[1] (kg m^{3-1})	Emission to the atmosphere[2] (kg m^{3-1})	Estimated Leakage % (kg kg gas^{-1}) [3]
CO_2, fossil	0.013	1.55	188,230,000	0.5	0.5	7.86E-04	7.86E-04	0.226
Mercury	-	-	3.1183	0.5	0.5	1.30E-11	1.30E-11	-
Halon 1211[4]	-	-	147	0.5	0.5	6.13E-10	6.13E-10	-
CH_4, fossil	0.72	85.60	34,684,000	0.7157	0.2843	6.57E-05	6.57E-05	0.024
NMVOC	-	-	209,060,000	0.9617	0.0383	4.34E-05	4.34E-05	-
Nitrogen oxides	0.007	0.83	250,000	0.5	0.5	1.04E-06	1.04E-06	-

[1] As calculated by equation 1.

[2] Emissions presented in Ecoinvent 3.8 process "Petroleum and gas production, off-shore – NO".

[3] As estimated by equation 2.

[4] Methane bromochlorodifluoro.

Table 18. Emissions Considered for the sensitivity analysis.

	Natural gas production, off-shore			Natural gas transmission		
	Base value [1]	High leakage [3]		Base value [2]	High leakage [3]	
Total leak (%)	0.028 %[4]	2.60 %	Comments	0.010 %	0.8 %	Comments
Compound	Emission (kg m^{3-1})	Emission (kg m^{3-1})		Emission (kg m^{3-1})	Emission (kg m^{3-1})	
CO2, fossil	7.86E-04	7.86E-04	High leakage values based on compounds presence in NG (kg Nm^{3-1}) adapted to 2.6% leaks	1.30E-06	1.04E-04	High leakage values based on compounds presence in NG (kg $Nm^{3\ -1}$) adapted to 0.8% leaks
CH4, fossil	6.57E-05	1.87E-02		7.19E-05	5.75E-03	
NMVOC	4.34E-05	4.45E-05		2.00E-07	1.60E-05	
NOx	1.04E-06	1.82E-04		7.00E-07	5.60E-05	Not found in dataset[2].Based on NG composition(kg $Nm^{3\ -1}$)
Halon 1211	6.13E-10	6.29E-10	Base emissions per 1m^3 adapted to 2.6% leakage	-	-	No data

[1] "petroleum and gas production, off-shore – NO" dataset

[2] "Natural gas, high pressure, import from NO" dataset

[3] Leakages as in Howarth & Jacobson (2021)

[4] 0.028% refers to the percentage of gas leaked over the produced gas

3.2.4.2. Biomethane

For Biomethane modelling we appealed to the LCIs in Antonini et al.[99]. These inventories include the biogas upgrading process via amine scrubbing, and the subsequent hydrogen production via SMR with CO_2 capture via MDEA technology, similar to the NG-based case. Finally, the LCIs include the transport and geological storage of CO_2, over a distance of 200 km via pipeline, and its injection in a saline aquifer at a depth of 800 m.

Biogas is produced by treating biowaste via anaerobic digestion (AD), which is the standard pathway for biogas production in Europe [127]. When obtained, biogas is saturated with water vapor- 6 to 12 % by weight- while methane and CO_2 presence in the gas composition range between 40 to 60% for both species [128]. This biogas is pretreated to remove H_2S and moisture before the gas upgrading unit.

Besides producing biogas, the AD of biowaste produces a co-product known as digestate which contains nutrients, organic carbon suitable for soils, and water, which enables its use as fertilizer in fields. Basically, the digestate is what was left of the original feedstock after the AD happens [129]. The digestate also exhibits potential for carbon sequestration in soils. Indeed, Antonini et al.[99] estimated that, when digestate is applied to the ground, and some carbon retention is observed, it could translate into negative life cycle GHG emissions, even if CCS is not integrated in the process.

In the Ecoinvent system model "allocation, cut-off by classification"[108], both biogas and digestate are categorized as co-products linked to the service of treating residual biowaste, and hence, both are deemed to be originally free of environmental burdens, which instead are allocated to the sector or activity producing the biowaste.

Thus, biomass is usually deemed as a carbon neutral energy resource as the emissions from biomass processing or combustion are compensated by the CO_2 uptake during plant growth; hence, a GWP of zero is often bestowed to biogenic CO_2 emissions in LCA studies [130]. Nonetheless, under this rationale, the impacts of biogenic carbon that is permanently removed from the atmosphere would not be correctly accounted. To correct this carbon mass violation, Antonini et al. [99] introduced the GWP_{100} characterization factors in the 5th IPCC [131] assessment report, which set a GWP_{100} value of 1 to biogenic CO_2 emissions and of -1 to "Carbon dioxide, to soil or biomass stock". This ensures that permanently storing biogenic CO_2 negative emissions in the calculations.

Furthermore, Antonini et al. [99] explored the digestate application on fields with the consequent transfer of carbon to the soil. Two cases were evaluated, representing lower and higher carbon sequestration. However, Antonini et al. [99] excluded the substitution of any fertilizers by the digestate, since it was considered a self-standing product due to the cut-off criteria applied to the AD process. Thus, any potential environmental advantages of digestate application should not be allocated to biogas. In contrast to AD, the burdens of biogas upgrading ought to be considered.

When the upgrading process is based in absorption, like in the case of amine scrubbing, the raw biogas meets the solution in a counter-flow scheme, always aiming to increase the contact area between the liquid and gaseous phases. The phenomenon enabling the absorption technique is that CO_2 is more soluble than methane in the amine. Thus, the liquid leaving the column contains an increased concentration of CO_2, whereas the gas mixture leaving the column contains larger concentrations of methane [132].

The main purpose of the upgrading process is to remove the CO_2 until it reaches a concentration of only 1 or 2% [128]. Selecting the proper technology for biogas upgrading is non-trivial as the specifics of the process could determine what technology is the most suitable. Amine scrubbing is one of the standard technologies for biomethane upgrading [133]. Other technologies are used to upgrade biogas: water scrubbing, membrane separation, cryogenic upgrading and PSA [128,129], which can be deployed individually or installed in series, depending on the project requirements. Although water scrubbing is currently presenting the largest market share [132,134], amine scrubbing presents cost and integration advantages [134]. Therefore, we judged that the amine-based upgrading is a solid candidate for future facilities integrating SMR and CCS.

Antonini et al. [99] included methane leakages during the upgrading process while no emissions were considered from the digestate as the process considered covered storage without any fugitive emissions. This is reasonable as AD and biogas upgrading facilities have incentives to prevent leaks throughout the entire

process. Due to the cutoff approach, the biogas leakages during AD should not be bestowed to biomethane. In any case IEA Bioenergy concluded that in biogas plants the majority of leaks are minor[135].

4. Transmission and distribution

As of 2021, only very limited amounts of hydrogen, mostly produced from fossil fuels, are transported in pure hydrogen form [89]. Nonetheless, the techno-economic model proposed by IRENA [89], anticipates that, by 2050, nearly a quarter of the worlds' hydrogen demand (18.4 EJ yr^{-1}) will be traded internationally. If such scenarios are to be met, it will require ramping up all the forms of hydrogen transportation, including pipelines, tanker ships, and trucks transporting CH_2, also known as tube trailers, and trucks transporting LH_2, labeled as tank trailers. However, it is uncertain how the transportation methods would integrate with each other to create Swedish hydrogen delivery chains, also labeled here as transportation pathways. In light of the strengths and weaknesses of each form of transport, the optimal choice of methods for delivery will depend on the specificities of each project [40,46,53,136–139].

Tanker ships are well suited for very large distances as in the case of transoceanic deliveries [40]. Pipelines are the most cost-effective option for very large capacities especially if it involves very short distances [40,140,141] since pipeline's levelized cost of transport (LCOT) decreases with transported capacity and increases with distance [137]. Likewise, Rödl et al.[46] estimated that transport via pipelines (t km) results in lower GHG emissions compared to CH_2 trucks and this trend accentuates as distances increase, however the environmental footprint of building the pipeline itself was not included. Wulf et al. [142] estimated that, for large capacities, pipelines present a lower LCOT, compared to CH_2 trucks, particularly for long distances. The European Commission [136] concluded that for pipeline lengths of 2,500 km, a distance compatible with the EU territory, transportation of hydrogen can be competitive with on-site production, if on-site production prices remain above 20 EUR MW^{-1}. Analogously, Blanco [111] suggested that hydrogen pipelines could be attractive for distances up to 3,000 km, when newly built, and 8,000 km if repurposed, emphasizing that costs increase directly with the length of the pipeline. Costs would diminish drastically if already existing NG pipelines could be repurposed for hydrogen [40,49,136]. However, as only one pipeline for NG transmission exists in western Sweden [143], the pipeline-based pathway in this study will necessarily be newly built.

For road transportation, tube trailers transporting CH_2 offer flexibility [15,40] and are considered as the most cost-effective way of delivering hydrogen to stations with capacities smaller than 500 kg per day [53]. Compared to pipelines, CH_2 trucks are competitive for distances less than 100 km, if the hydrogen refueling stations (HRS) to be supplied display small capacities [142]. In contrast, for large capacities CH_2 are estimated to result in the highest supply chain costs, due to low capacity of tube trailers of around 1,000 kg [137] which is also associated with higher GHG emissions [15]. In the case of a network of HRSs supplied with CH_2, the cost of storage and transportation is anticipated to increase significantly [137].

Moreover, transport of LH_2 via tanker trucks needs large capital investments and is less frequently employed. However, it allows for the delivery of substantially larger quantities, around 4 t [15], making it competitive for long distances and large amounts, compared to CH_2 [40,53,137,140,144], while also being advantageous where end-users require high purity. The competitiveness of LH_2 delivery over long distances improves to the point that it could overlap with pipeline performance [53,111]. Different distances are suggested in literature for the tradeoff between CH_2 and LH_2 truck transports (in the range of 130km-1,000km). Frank et al. [18] estimated the distance where LH_2 becomes more competitive than CH_2 to be 1,000 km but these results are sensitive to the trailer cost, payload capacity and CH_2 storage size. Furthermore, the GHG emissions of LH_2 delivery are higher than those of CH_2 delivery for distances of less than 800 km, when energy is obtained from California's grid, even if ultra-low energy requirements for liquefaction are included (6 kWh kg ${H_2}^{-1}$) [18]. Rödl et al.[46] estimated the breakeven point, where LH_2 transport starts to be cheaper than CH_2 transport, to be above 450 km, driven once more by the lower energy consumption per kg*km of LH_2, associated to the larger capacity of the LH_2 truck. Reuß et al. [137] estimated the breakeven point for CH_2 and LH_2, at a supply chain level, as low as 130 km.

The results of the studies attempting to minimize costs or GHG emissions heavily depend on the specific assumptions of each scenario, including infrastructure choices and energy consumption by each delivery chain. In a more general way, the choice of producing hydrogen on a central plant or at the HRS relies on the tradeoff between production and transportation costs. The improvements of economies of scale and process efficiencies will decide if the advantages of transporting hydrogen from low-cost production areas to high-demand areas offset the benefits of on-site production [89]. For a Swedish scenario, Lundblad et al. [145] estimated that, although renewable-based hydrogen, produced in a distributed way, presents higher costs compared to centralized production, the additional costs of transport, especially during distribution, could render centralized production the most expensive option.

Despite focusing on the environmental footprint, our study aimed at proposing pathways that could represent a credible economic alternative, at least for a period during the evolution of a hydrogen market. The literature review identified different forms of transport as being more advantageous than others depending on the project size and distances, thereby, some selected pathways are expected to represent initial low volumes, like in the case of CH_2 trucks, while the transmission via pipeline would represent a case where demand has been estimated to compensate the high investment required. Concerns of pipeline affecting hydrogen purity [49,76,143] motivated the inclusion of a purification stage.

Compression and liquefaction, also referred to as packing, have been identified as representing the lion's share of the energy needs, throughout the transportation chain [15,18,137,144]. In consequence, we only included liquefaction for pathways where the FCT is refueled with LH_2, thereby, keeping the hydrogen liquid over the entire delivery chain, under the assumption that liquefying twice in the same delivery chain would be economically unsustainable. Besides, due to its high costs and elevated energy consumption [40,146], we discarded LH_2 for storage when the hydrogen is produced at the HRS. Therefore, for distributed production, hydrogen is exclusively stored in gaseous form.

Although LH_2 could be brought into the HRS and then gasified for refueling of CH_2 trucks [15,40,45], the life cycle impact assessment (LCIA) results of such pathway are expected to fall somewhere in between the purely liquid and the purely gaseous options. However, future LCA cases could explore the environmental footprint of delivery chains that include phase changes. Moreover, compression requirements were reduced wherever possible. For instance, the gaseous storage facilities at the production plant were assumed to comprise of spheric vessels at 10 Bar. As a consequence, the required storage volume is larger than it would be required at higher pressures.

The adoption of large infrastructures for hydrogen transmission and storage will require demand from multiple economic sectors. Besides long-haul heavy-duty transport, hydrogen is a good candidate for decarbonizing other hard-to-abate sectors such as refineries, steel mills, chemical industries, aviation, and shipping [69,147]. In Sweden, it is expected to contribute to raw materials refining, granting large reductions in GHG emissions [148]. In front of tight budgets, IRENA [68] suggests that initial strategies should focus on applications where hydrogen requires less economic support.

Tables 19,20,21 and 22 present the energy requirements associated with each transportation pathway. For packing we included base and optimistic values which are used in the sensitivity analysis.

Table 19. Packing energy consumption and storage parameters at the production plant for each transportation pathway.

	Technology	Origin	Storage LH_2	Storage CH_2	Packing-transmission (kWh/kgH_2) Optimistic & base (1)	Transfer to transmission (kWh/kgH_2) (2)
A	Green (30 Bar & 20 C)	Sweden	-	Spherical vessel 10 Bar	500 Bar- tube trailer 2.19 2.63	Already Included
B			Cryogenic tank 20 K	-	20 K- tank trailer 10 13	Cryopump 0.08
C			-	Pipeline acts as storage	70 Bar- pipeline 0.47 0.57	Already Included
D		Chile (3)	Cryogenic tank 20 K	-	20 K- tank trailer 10 13	Cryopump 0.08
A	Blue (26 Bar & 20 C)	Sweden	-	Spherical vessel 10 Bar	500 Bar- tube trailer 1.65 1.99	Already Included
B			Cryogenic tank 20 K	-	20 K- tank trailer 10 13	Cryopump 0.08
C			-	Pipeline acts as storage	70 Bar- pipeline 0.55 0.67	Already Included
D		Norway	Cryogenic tank 20 K	-	20 K- tank trailer 10 13	Cryopump 0.08

(1) Packing required for transmission. For gaseous pathways, base case and optimistic case represent 50% and 60% compressor efficiency respectively. For Pathway A, compression starts at 10 Bar, the pressure for storage at the spherical vessels. For Pathway C, compression starts at the pressure at 26 Bar, the production output. Liquefaction energy consumption was retrieved from literature review.
(2) Refers to transfer from the storage at the production plant to the transmission method
(3) For hydrogen production in Chile electricity was assumed to be 100% wind. For liquefaction at the port, we considered the Chilean electricity mix. Liquefaction in the Norwegian port is also powered by the electricity grid.

Table 20. Energy consumption and transmission parameters for each transportation pathway.

	Transmission method & distance	Fuel use (1) (kWh/kgH_2)
A	Tube trailer at 500 bar - 150 km	1.01
B	Tank trailer at 20 K - 150 km	0.33
C	Pipeline at 70 bar - 150 km	0.02
D	Tanker ship at 20 K - 14,200 km	2.50
A	Tube trailer at 500 bar - 150 km	1.01
B	Tank trailer at 20 K - 150km	0.33
C	Pipeline at 70 bar - 150 km	0.02
D	Tanker ship at 20 K- 1,000 km	0.18

(1) For pathways A & B, it refers to the fuel use per traveled distance per kg H_2 as in Liu et al[15]. For Pipeline, it refers to energy for recompression every 100 km as in Rödl et al[46]. For tanker ship it refers to biofuel use as in European commission [19].

Table 21. Packing energy consumption and storage parameters at the GotHUB for each transportation pathway

	Packing - storage (kWh/kgH_2) Optimistic & base (1)	Purification (kWh/kgH_2) (2)	Packing-distribution (kWh/kgH_2) Optimistic & base (3)	Transfer to distribution (kWh/kgH_2) (4)
A	N/A	Not required	-	-
B	N/A	Not required	-	-
C	135 Bar- LRC<br>0.37 0.44	1.0 - PSA	500 Bar- tube trailer<br>0.73 0.88	Already Included
D	Cryopump<br>0.08	Not required	Active cooling<br>0.0407	Cryopump<br>0.08
A	N/A	Not required	-	-
B	N/A	Not required	-	-
C	135 Bar- LRC<br>0.37 0.44	1.0 - PSA	500 Bar- tube trailer<br>0.73 0.88	Already Included
D	Cryopump<br>0.08	Not required	Active cooling<br>0.0407	Cryopump<br>0.08

(1) Required for storage at the GotHUB. For pathway D, it refers to the pumping from the tank trailer to the LH_2 dome.
(2) Purification via PSA considered as 3.6 MJ Kg H_2 [149].
(3) In pathway C it refers to compression required to reach the tube pressure for distribution. In pathway D, it refers to active cooling intended for boil-off reliquefication. Estimated by assuming a 0.01% boiloff and an energy consumption of 4.07 kWh kgH_2^{-1} [19].
(4) Refers to transfer from storage at the GotHUB to the distribution truck.

Table 22. Energy consumption and distribution parameters for each transportation pathway.

	Distribution method & distance (1)	Fuel use (2) (kWh/kgH_2)
A	Already included	-
B	Already included	-
C	Tube trailer at 500 Bar – 50 km	0.33
D	Liquid trailer at 20 K – 50 km	0.11
A	Already included	-
B	Already included	-
C	Tube trailer at 500 Bar – 50 km	0.33
D	Tanker ship at 20 K- 1,000 km	0.11

(1) In pathways A & B, transmission and distribution are performed by the same truck.
(2) Estimated based on Liu et al. [15]

4.1. Packing

Packing is a general term that refers to the compression or liquefaction processes. Table 19 and Table 21 present the energy requirements for each delivery pathway.

4.1.1. Compression

As the lightest element, hydrogen occupies large volumes at ambient pressure and temperature [77]. Compressing the hydrogen increases its density. At 350 Bar, density is nearly 23 kg m^{-1}, while at 700 Bar, it is 38 kg m^{-1} [44]. Compared to LiBs, compressed hydrogen (CH_2) at 70 bar exhibits an energy density 300 times higher per unit weight, and six times higher per unit volume [53]. In contrast, hydrogen exhibits a lower energy density than other energy carriers. In fact, at the same pressure, a cubic meter of hydrogen contains one third of the energy in a cubic meter of NG [143].

Compression is a mature technology that enables transportation and storage of hydrogen by increasing its density [150] while also offering advantages in terms of rapid refueling. In addition, it only requires a third of the energy that liquefaction does [43], without dealing with the challenges of ultra-low temperatures.

Despite this, compression is still energy-intensive and significantly contributes to transportation costs [43–46,96,150]. Besides, the energy density increase associated with compression is not limitless. Even if compressed at 800 Bar, hydrogen requires three times more volume than gasoline for the same energy content [150].

To estimate the compression work required for each stage of the process we assumed isothermal compression. The formula provided by Hirscher [151] is the following:

$$W = n * R * T(\frac{P2}{P1})$$

Isothermal compression assumes that cooling is continuously applied to keep the temperatures constant by removing the heat generated during compression. This implies an additional energy expenditure, and a consequent efficiency decrease for the compression system. As cooling is not always available, many industrial compressors are indeed adiabatic. Adiabatic compression requires more energy input. Aiming to save energy, a multistage intercooled compression process is often found [96,98].

The energy consumption for compression depends on the specificities of each system and proposing a detailed layout of the compression system, including cooling and other auxiliaries, is beyond the scope of this study. However, since the energy demand strongly depends on the initial and final pressures of the hydrogen to be compressed, [46] we deemed that the isothermal compression described by Hirscher [151] was adequate to estimate the compression requirements in this study.

A more precise calculation of the work requirements for isothermal compressors would require performing a pressure-dependent correction specific to hydrogen [96]. Nonetheless, we considered the formula in Hirscher [151] to be a valid approximation, in consonance with the estimations performed by Bekel & Pauliuk [5]. Aiming to obtain a conservative estimation we included low efficiencies of 50% in contrast to efficiencies for adiabatic compressors which can reach up to 80% [19]. For a more optimistic case that is included in the sensitivity analysis, we included efficiencies of 60%.

Compression infrastructure, *i.e.* compressors, pipes, valves, gauges, etc., was not included in the analysis. However, its LCIA contribution per traveled km is expected to be very small. The energy consumption requirements for compression are summarized in Table 19 and Table 21.

4.1.2. Liquefaction

Liquefaction typically begins with a precooling step where liquid nitrogen is used to take hydrogen from ambient temperature to 80 K. This process is then followed by a series of compression / expansion stages to reach the liquefaction temperature of 20 K [15,96]. This process involves compressors, expansion devices, heat exchangers and throttling valves [152] and requires very pure hydrogen [96]. Liquefaction facilities come at a high capital cost [40,44,137,146].

Although liquefaction increases hydrogen's volumetric energy density, making it twice as high as that of compressed hydrogen at 700 bars [43], it still occupies three times more volume than gasoline, for the same energy content [98]. Furthermore, the energy required for liquefaction represents nearly a third of the chemical energy stored in the hydrogen, based on its lower heating value (LHV) of 120 MJ kg^{-1} [43,98,111].

The energy demand for liquefaction according to the literature review is presented in Table 23. Energy consumption is influenced mainly by the energy efficiency of the compression / expansion stages within the system and by the hydrogen supply pressure [15]. The liquefaction of hydrogen has been documented since the beginning of the twentieth century [40] and as technologies evolve, energy consumption is expected to decrease. The energy consumption requirements for liquefaction are exhibited in Table 19 and Table 21.

Table 23. Literature review results for the energy consumption of liquefaction

Study	Consumption (kWh kg H_2^{-1})	Notes
Di Profio et al.[153]	12-15	Based on data from 2000's
Idealhy.eu [154]	12	Reported to be state-of-the-art in 2013
Liu et al. [15]	11-15	
Reuß et al. [137]	12-15	
Reuß et al. [155]	6-7	Forecast for new liquefaction cycles. Including heat recovery and precooling cycles
Frank et al. [18]	8.2	Based on a 131 tpd liquefier from HDSAM model
Frank et al. [18]	9.4	Based on a 33 tpd liquefier from HDSAM model
Argonne [82]	11	Estimation for a 10 tpd liquefier. HDSAM model
Rödl [46]	10	Assumption based on literature review
Cardella et al. [156]	6.7-7.5	Best case using new liquefaction cycles
RISE [40]	10-15	Reported by their literature review
Berstad et al. [157]	7-10	Assuming improvement of techniques
Holladay et al [158]	6-7	Applying magnetic refrigeration
Aasadnia et al. [146]	13-15	Based on Ingolstad plant in Germany. 4.4 tpd.

Apart from the high energy requirements, LH_2 requires expensive double-hulled cryogenic flasks intended to keep hydrogen from reaching the boiling temperature of 20 K [40,96,98,111] and also as a safety measure to avoid flashing [137,140]. Despite this generous thermal insulation some heat will always transfer into the vessel, which causes the LH_2 to warm up and vaporize, this evaporation is known as boil-off [46,48,96,150]. When boil-off appears, some of the vaporized hydrogen must be released to avoid excessive pressures within the vessel [40,96,98,111] which means that cryogenic tanks must have provisions for venting, active cooling [44] or recirculation of the boil-off into a fuel cell to produce electricity [19].

Boil-off is a significant problem when hydrogen is stored in vessels for long periods since it is steadily produced by the heat transfer from outside the flask. Furthermore, boil-off can also be produced by the conversion from ortho hydrogen to para hydrogen, which are the two isomeric forms of molecular hydrogen. At ambient temperature and pressure, hydrogen comprises 75% ortho and 25 % para, but this changes as temperatures decrease; at 30 K para hydrogen makes up nearly 100% of the molecular hydrogen. This conversion does not take place instantly during the liquefaction but happens over time. Since ortho is at a higher energy state compared to para, the net result is an energy release which increases temperature, producing boiloff [40,96].

Small-scale liquefiers can be found in industrial applications [40]. Nonetheless, a future LH_2 supply chain is very likely to be centralized, with large liquefaction facilities integrated into the production hub, from which the hydrogen would be subsequently distributed [40,146]. This inclination towards centralized liquefiers is driven by lower efficiencies and higher costs of small-scale liquefiers. In addition, in large-scale liquefaction facilities, boil-off is easier to reliquefy [40]. See Table 33 and Table 34.

Hydrogen liquefaction increases costs and energy use at the initial stages of the supply chain [40]. However, in later stages, the energy requirements and costs diminish as LH_2 equipment is more efficient, compared to CH_2 equipment. Therefore, LH_2 supply chains could be more economic than CH_2 supply chains in the future [40]. For this, losses in the LH_2 chain must be addressed. Losses associated with LH_2 are due to either vented gas or the energy required for active cooling [40]. Venting has also been identified as a significant contributor to the environmental burden of heavy-duty onboard applications [22]. As of 2023 there were no commercially available HRS delivering LH_2 [40], however, from a purely technical perspective, there is no reason for why it should not be a viable option.

The liquefier was not included in the analysis. However, its LCIA contribution per traveled km is expected to be small.

4.2. Transport

CH_2 is delivered to the station via pipeline or by truck, more specifically, by tubes incorporated onto the truck trailer. On the other hand, LH_2 is transported via tanker ships or trucks, which are also known as tank trailers. In Pathways A & C hydrogen is transported in gaseous form while for pathways B & D hydrogen always remains a liquid. Moreover, pathways A & B consider direct transportation from the production plant to the HRS via trucks while pathways C & D include hydrogen storage, at the city gates, in the supply hub labeled as GotHUB. Pathway C includes an extra step of purification aiming to address concerns of pipeline affecting hydrogen purity.

The transportation distance in pathways A & B (150 km), where hydrogen is transported directly from the production plant to the HRS is shorter than the combination of pipeline for transmission and truck for distribution found in pathway C (200 km), as the trucks are able to take shorter paths to arrive to their destination. Pathway D includes importing hydrogen from other countries in liquid state. Energy consumption linked to transportation is depicted in Table 20 and Table 22.

4.2.1. Gaseous

Transportation parameters and infrastructure assumptions for pathways based in gaseous hydrogen can be found in Table 24.

Table 24. Transmission and distribution parameters for gaseous hydrogen-based pathways.

	Pathway A	Pathway C
	Transmission	
Source-destination (distance)	Central plant-HRS (150 km)	Central plant – GotHUB (150 km)
Carrier	Tube trailer	Pipeline
Capacity (Kg H_2)	1,000	
Fuel / energy source	Low-sulfur Diesel. LHV: 10.02kWh L^{-1}. Density: 0.847 kg L^{-1}	Electricity-grid (recompression)
Fuel/ energy dataset	diesel production, low-sulfur, petroleum refinery operation | diesel, low-sulfur	Swedish grid (Ecoinvent)
Energy consumption (kWh kgH_2^{-1})	1.01 [15]	0.02 linked to recompression every 100 km as in Rödl et al. [46]
Carrier dataset (Ecoinvent)	lorry, 16 metric t	Pipeline dataset as in Wulf et al. [142]
Life expectancy	3,333 t H_2 throughout the tanker lifetime. Assuming 1,000,000 km total lifetime and trips of 150 km with empty return.	Internal diameter of 20 cm as in Wulf et al.[142]. Assumed flow speed is 10 m s^{-1} and density at 70 bar is 5.6 kg $m^{3-1,}$ resulting in a capacity of 6,336 kg hr^{-1} . Under a load factor of 5,000 hr yr^{-1} [143] and a lifetime of 40 yr [46] the pipeline transports 1.267E9 kg H_2 during its lifetime.
Transport Emissions (g kWh^{-1})	Estimated based on Combination Short-Haul Truck CIDI - LS Diesel [27] In g kWh^{-1}: VOC: 9.29E-3 ; CO: 3.62E-1; NOx: 2.36E-1; PM10: 5.20E-4; PM2.5: 7.70E-4; ; CH_4: 2.12E-3; N_2O: 3.23E-4; CO_2: 587.2. Engine efficiency: 45%	N/A
Estimated speed & time	80 km hr^{-1}, 2 hours	10 m s^{-1}
Boiloff rate	0.1 Own assumption	1 % [19]
	Distribution	
	Pathway A	Pathway C
Source-destination (distance)	For pathway A, transmission and distribution take place in the same CH_2 truck	GotHUB - HRS (50 km)
Carrier		Tube trailer
Capacity (Kg H_2)		1,000
Fuel / energy source		Diesel
Fuel/ energy dataset		diesel production, low-sulfur, petroleum refinery operation | diesel, low-sulfur
Energy consumption (kWh kgH_2^{-1})		0.33 Extrapolated from [15]
Carrier dataset (Ecoinvent)		lorry, 16 metric t
Life expectancy		10,000 t H_2 throughout the tanker lifetime. Assuming 1,000,000 km total lifetime and trips of 50 km with empty return.
Transport Emissions (g kWh^{-1})		Estimated based on Combination Short-Haul Truck CIDI - LS Diesel [27] In g kWh^{-1}: VOC: 9.29E-3 ; CO: 3.62E-1; NOx: 2.36E-1; PM10: 5.20E-4; PM2.5: 7.70E-4; ; CH_4: 2.12E-3; N_2O: 3.23E-4; CO_2: 587.2. Engine efficiency: 45%
Estimated speed & time		80 km hr^{-1}, <1 hours
Boiloff rate		0.1 % [19]

4.2.1.1. *Tube trailer*

Tube trailers, towed by heavy trucks, are the established technology for transport of GH_2 [40], whereas pipelines are still restricted to a few locations linked to industrial hubs [143]. These tube-trailer systems are configured to carry between 300 and 1,100 kg at a pressure between 200 and 500 bar [15,46] and are made of CrMo steel alloys, same material used in valves and safety devices [140].

Higher pressures, and therefore capacities, are possible. Especially when type IV tanks, based on carbon fiber, are used [46,140]. Nonetheless, we did not include type IV tanks for CH_2 transportation. This choice is primarily driven by the high costs and GHG footprint of carbon fiber tanks [6,22] which could render hydrogen demand insufficient to compensate for the high capital investment. Besides, transportation via tube trailer is a mature technology. In addition, carbon fiber tanks are highly carbon intensive [22].

Theses tubes used for transportation can also be employed as stationary storages in HRSs where other storage methods are not available [140] simply by swapping them with empty tubes that return to the production facility [45], but this study considers the hydrogen is transferred from the tube to the storage at the HRS. The tube trailers in this study were assumed to transport 1,000 kg of hydrogen at 500 bar as in Liu et al.[15].

4.2.1.2. *Pipeline*

An alternative to transporting CH_2 in tube trailers is to convey it via pipeline, which eliminates the driving time required for the tube trailer to go back to the production plant while also being well suited for large flows [46,49,111,143,159] and have a large potential for energy transmission: a 48-inch pipeline, the largest in the intra-EU gas network, can transport approximately 17 GW while a 36-inch pipeline could achieve around 9 GW, based on LHV [143]

Hydrogen pipelines are economically attractive for short distances or high flows [111,159,160] and have been considered as part of the hydrogen strategies in Europe [49,143,161]. However, compared to NG, hydrogen pipelines are still scarce, located between industrial clusters and used to transport fossil-based hydrogen. As of 2021, there were approximately 1,600 km of pipelines in the EU [143] while around 2500 km had been installed in the U.S. primarily along the Gulf Coast [141].

Nevertheless, as hydrogen's volumetric energy density is only a third of that of NG (at the same pressure), transporting equivalent amounts of energy requires a volumetric flow three times higher for the hydrogen pipeline [140,162]. In contrast, hydrogen's density is nine times lower than that of NG and has a lower viscosity implying that hydrogen can reach higher speeds, and therefore, higher volumetric flow rates (nearly three times that of NG) if same pipeline diameter and operating pressure are used. These opposite characteristics result in the energy transported by a hydrogen pipeline, per unit of volume, being approximately only 80% of that of a NG pipeline [143,162].

Hydrogen production centers are unlikely to be located too far away from demand hubs like the city of Gothenburg; however, the precise location and dimensions of the pipeline are unclear. We consider 150 km as the pipeline length between the production plant and the GotHUB, while the distribution leg is 50 km in pathway C. Pipeline pressure was assumed to be 70 bar, a value supported by several studies [45,46,143,163].

4.2.1.2.1. Pipeline repurposing

The hydrogen pipelines built in the past decades have been based in NG pipeline technology [140], and are manufactured in similar ways, using steel or cast iron, but having different requirements for the welded seams and internal coatings [46,52,164]. Joints and special parts are manufactured using low carbon steel or tempered steels, while no superficial defects must be detected [140].

With some alterations, NG pipelines could be able to transport the smaller hydrogen molecules [96,143]. One of main differences between NG and hydrogen pipelines is the need for a special coating to prevent diffusion [96,140,143,164] which might result in the process known as hydrogen embrittlement [52,76,143,164]. Moreover, pipeline use is expected to affect hydrogen purity, especially for cases where end-use is based on fuel cells [49,76,143].

Therefore, for end-users requiring high purity levels, like the case of FCTs, extra purification systems will be required as included in pathway C in this study. Further elaboration is included in the purification section.

Repurposing gas pipelines for hydrogen transport is recognized as an option to limit the otherwise gargantuan capital costs of acquiring the land and building the pipeline. IRENA [139] has estimated that repurposed pipelines are 65-94% cheaper than newly built ones, while also reducing the environmental footprint. The European hydrogen backbone is a project articulated by big players in NG production and distribution in the EU [143], aiming to create a hydrogen pipeline network extending for 6,800 km in Europe by 2030. The network is intended to connect the so-called hydrogen valleys, which are regions of high demand, to suppliers. By 2040 the aim is to reach 23,000 km. The projected pipeline network will be predominantly (75%) based on repurposed pipelines which would work under their nominal capacity, at least initially, resulting in savings in compressors acquisition and energy consumption [143].

In 2018, Gasunie inaugurated a 12-km pipeline in The Netherlands that was repurposed to hydrogen and has been in operation since then, with no public reports of issues [165,166]. Furthermore, Gasunie have stated that the compressors were not going to be replaced during the first phases of the project, as pressure was delivered at the entry points, and hydrogen volumes were still low. In 2022 the Dutch government announced plans to construct the first hydrogen grid in the world, but this kind of projects are expected to be harder to replicate elsewhere as the NG grid in The Netherlands in probably unique in terms of redundancy and robustness [166].

The Swedish NG transmission system in 2021 comprised of a single pipeline that runs from Dragör, Denmark to Stenungsund, Sweden, on the country's west coast. This transmission system is 601 km long while a distribution system's length is around 3,500 km [167]. However, if the pipeline were to be repurposed for hydrogen it would imply stopping the NG flow. That is unfeasible, and thereby, any Swedish hydrogen pipelines will have to be newly built [148]. This conclusion is shared by the EHB initiative which considers that the hydrogen pipelines reaching Gothenburg region would be newly built while a connection with the European grid would only happen in 2040 [143]. Other studies have analyzed the opportunities and obstacles for developing hydrogen infrastructures in northern Scandinavia and Finland, including hydrogen pipelines [148]

4.2.1.2.2. Hydrogen embrittlement

When hydrogen is in direct contact with the pipeline walls, or any other metallic surfaces, it can accelerate pipeline degradation in a process known as hydrogen embrittlement, where hydrogen is absorbed or permeated through the metal, inducing cracks on it [52,141]. The physical phenomena leading to hydrogen embrittlement is complex and is dependent on several factors such as, the pipeline operating pressure [52,140], the alloy elements in the steel, the specific microstructural features of the metal [48], and the presence of impurities in the hydrogen [52].

Regarding the specific steel choice, some austenitic steels families have been identified as having small susceptibility to hydrogen embrittlement [52]. For instance, in ambient conditions, hydrogen has been proven to negatively affect the mechanical properties of austenitic stainless steels, nonetheless, since their fracture toughness is high, the structural integrity is rarely compromised [48]. However, Campari et al. [52] stated that hydrogen embrittlement associated with fatigue is a cause for concern considering pressure changes. Furthermore, some impurities in the hydrogen can be corrosive to pipelines [140], namely, moisture and H_2S [96,143]. In contrast, some impurities like oxygen and carbon monoxide actually can diminish the growth rate of fatigue cracks [52].

There are a range of options to tackle hydrogen embrittlement, including: a) the use of ductile steels ; b) regularly checking the crack growth via pigs; c) avoiding pressure variations to prevent crack formation; and d) applying a coating layer on the inner surface of the pipeline to avoid direct contact with the hydrogen [52,143]. Research on coating layers for hydrogen pipelines has identified Ti and Al_2O_3 based coatings as promising compounds [164] whereas commercial solutions are already available in the market [168]. In contrast, Wang et al. [143] reported hydrogen repurposing projects in the Netherlands and Germany that would prove that existing pipelines in those regions do not necessitate internal coating, however, the source was not disclosed [143].

4.2.1.2.3. Recompression

While travelling through the pipeline the hydrogen pressure diminishes and needs to be recompressed regularly. Due to its lower molar mass, and larger volume flows, higher energy requirements for hydrogen are expected compared to NG transport [140,143] The potential of current compressors to work on repurposed pipelines is uncertain and should be evaluated on a project-basis [143]. In addition, compressor lubricants should be checked for hydrogen tolerance, keeping in mind that exposing the pure hydrogen to such hydrocarbons could affect purity. In such cases, diaphragm compressors are a suitable solution as a membrane avoids the hydrogen of being in touch with lubricants.

For our pipeline transportation case we assumed that a recompression station is necessary every 100 km as in Rödl et al. [46] while for maintenance we will consider a 2% of the emissions linked to pipeline manufacturing, per year as in Rödl et al. [46] as well. The pipeline represented in this study has an outer diameter of 86.4 cm while the thickness is 3.2 cm [19].

4.2.2. Liquid

Transportation parameters and infrastructure assumptions for pathways based in liquid hydrogen are presented in Table 25.

Table 25. Transmission and distribution parameters for liquid hydrogen-based pathways.

	Pathway B (GH_2 & BH_2)	Pathway D (GH_2)	Pathway D (BH_2)
Stage	Transmission		
Source-destination (distance)	Central plant-HRS (150 km)	Antofagasta, Chile-GotHUB (14,200 km)	Molde, Norway- GotHUB (1,000 km)
Carrier	Tank trailer	Tanker ship	Tanker ship
Capacity (Kg H_2)	4,000	9,800 E3	9,800 E3
Fuel	Diesel	Biodiesel	Biodiesel
Fuel dataset (Ecoinvent)	diesel production, low-sulfur, petroleum refinery operation | diesel, low-sulfur	esterification of soybean oil | fatty acid methyl ester | BR-RS. The state of Rio Grande do Sul (RS) is the largest producer of biodiesel. [169] Emissions are estimated as 38.8 gCO2 MJ^{-1}	esterification of soybean oil | fatty acid methyl ester | BR-RS. The state of Rio Grande do Sul (RS) is the largest producer of biodiesel. [169] Emissions are estimated as 38.8 gCO2 MJ^{-1}
Energy consumption (kWh kgH_2^{-1})	0.33 [15]	19 g per kg of H_2 per km [1919], equivalent to 2.84 kWh.	19 g per kg of H_2 per km [19], equivalent to 0.20 kWh.
Carrier dataset (Ecoinvent)	lorry, 16 metric t	tanker, for liquefied natural gas	tanker, for liquefied natural gas
Life expectancy	13,332 t H_2 throughout the tanker lifetime. Assuming 1,000,000 km total lifetime and trips of 150 km with empty return.	1.53 Mt H_2 throughout the tanker lifetime. Assuming 80,000 nautical miles yr^{-1} for 30 years and 14,200 km trips with empty return.	21.78 MtH_2 throughout the tanker lifetime. Assuming 80,000 nautical miles yr^{-1} for 30 years and 1,000 km trips with empty return.
Transport Emissions (g kWh^{-1})	Low-sulfur diesel for HDT [27] In g kWh^{-1}: VOC: 0.02 ; CO: 0.64; NOx: 0.38; PM10: 0.02; PM2.5: 3.96E-3; SOx: 1.85E-3; CH_4: 3.1E-3; N_2O: 4.64E-4; CO_2: 265.7.	Emissions considered as if using MGO in 2 stroke engines [170]. Emissions other than CO_2 are expected to be similar to Biodiesel. In g kWh^{-1}. VOC: 0.3 ; CO: 0.7; NOx: 3.4; PM10: 0.215; SOx: 0.33; CH_4: 0.01; N_2O: 0.03; NH_3: 0.05; CO_2: 549. Diesel density: 0.88 kg L^{-1}. LHV: 9.26 kWh L^{-1}.	Emissions considered as if using MGO in 2 stroke engines [170]. Emissions other than CO_2 are expected to be similar to Biodiesel. In g kWh^{-1}. VOC: 0.3 ; CO: 0.7; NOx: 3.4; PM10: 0.215; SOx: 0.33; CH_4: 0.01; N_2O: 0.03; NH_3: 0.05; CO_2: 549. Diesel density: 0.88 kg L^{-1}. LHV: 9.26 kWh L^{-1}.
Trip speed & time	80 km hr^{-1}, 2 hours	17 knots, 20 days	17 knots, 1.4 days

Boiloff rate	0.3% [19]	0.2 % day^{-1} [19]	0.2 % day^{-1} [19]
Stage	Distribution		
Source-destination (distance)	For pathway B, transmission and distribution happen in the same CH_2 truck.	GotHUB-HRS (50 km)	GotHUB-HRS (50 km)
Carrier		Tank trailer	Tank trailer
Capacity (Kg H_2)		4,000	4,000
Fuel		Diesel	Diesel
Transport vehicle dataset		diesel production, low-sulfur, petroleum refinery operation | diesel, low-sulfur | Cutoff, U	diesel production, low-sulfur, petroleum refinery operation | diesel, low-sulfur | Cutoff, U
Energy consumption (kWh kgH_2^{-1})		0.11, extrapolated from [15]	0.11, extrapolated from [15]
Life expectancy		40,000 t H_2 throughout the tanker lifetime. Assuming 1,000,000 km total lifetime and trips of 50 km with empty return.	40,000 t H_2 throughout the tanker lifetime. Assuming 1,000,000 km total lifetime and trips of 50 km with empty return.
Emissions		Estimated based on Combination Short-Haul Truck CIDI - LS Diesel [27]. In g kWh^{-1}: VOC: 9.29E-3 ; CO: 3.62E-1; NOx: 2.36E-1; PM10: 5.20E-4; PM2.5: 7.70E-4; ; CH_4: 2.12E-3; N_2O: 3.23E-4; CO_2: 587.2. Engine efficiency: 45%	Estimated based on Combination Short-Haul Truck CIDI - LS Diesel[27] In g kWh^{-1}: VOC: 9.29E-3 ; CO: 3.62E-1; NOx: 2.36E-1; PM10: 5.20E-4; PM2.5: 7.70E-4; ; CH_4: 2.12E-3; N_2O: 3.23E-4; CO_2: 587.2. Engine efficiency: 45%
Trip speed & time		80 km hr^{-1}, 80 km hr^{-1}, less than 1 hour	80 km hr^{-1}, less than 1 hour
Boiloff rate		0.1 %. Own assumption	0.1 %. Own assumption

4.2.2.1. *Tank trailer*

Tank trailers transporting LH_2, via road to the HRS, were assumed to have a transport capacity of 4,000 kg near atmospheric pressure. This approximation is in line with currently available tank trailers [15,46,137] which are estimated to have a curb weight of 24,400 kg [46]. Therefore, compared to tube trailers transporting 1,000 kg of CH_2, tank trailers would transport around four times more hydrogen [137,171]. Similarly to tube trailers, tank trailers can also be unloaded or swapped at the HRS [40]; this study assumes the tankers are unloaded at the destination.

4.2.2.2. *Tanker ship*

Overseas transport of LH_2 is carried out by tanker ships with a similar design to those transporting LNG [19,136]. As of 2024 we found no confirmation of any commercially available LH_2 ship tanker. Nevertheless, Kawasaki has been developing a hydrogen liquid tanker prototype for around a decade [172]. In 2023 the company announced that the technological development for the LH_2 tank had been completed [173]. The ship will be equipped with four 40,000 m^3 tanks for a total cargo of 160,000 m^3, in addition, the ship is intended to exhibit dual-fuel technology, allowing it to work on both low-sulfur fuel oil and hydrogen which opens the possibility of using boil-off for ship propulsion [174]. Further data, such as energy consumption or whether the LH_2 is pressurized were not available.

The tanker data was obtained from European Commission [19], which describes a ship with double-hulled cryogenic tanks able to carry 9,800 t of liquid hydrogen with a fuel consumption of 19 g of Biodiesel per t of H_2 per km. Biodiesel was assumed to be 100% produced from soybean with a lower heating value (LHV) of 9.3 kWh Kg^{-1}. Although most tanker ships run on fuel oil, the biodiesel-based ship was considered an acceptable alternative. Since the LH_2 tankers are still in development, and boil-off could be used for the ship energy needs, determining the fuel consumption is not straightforward and the specificities are out of the scope of this study. We considered that 0.2% of the hydrogen would need to be vented per day [19].

The ship data was originally based on LNG tankers and then adapted to hydrogen [163].The region of Antofagasta, Chile was selected as location for GH_2 production in Chile, which results in 14,200 km. For BH_2 production in Norway the assumed distance is 1,000 km. Due to this short distance, choosing a tanker for transportation seems controversial at first, but we discarded other pathways, like pipelines, due to high capital costs, while 1,000 km is too long a distance for trucks to be economically feasible. For all cases, we considered empty return of the truck. Table 25 presents displays data assumptions for transportation.

4.3. Storage

Hydrogen supply chains require storage capacity in order to cope with surges in demand or to guarantee a minimum supply if production pace diminishes. This is necessary independently of production being centralized or distributed. However, choosing the most adequate storage method depends on the nature of each delivery chain, the amount stored, the available capital, the operating costs and the storage period [44]. Thereby, distributed production facilities will only require enough storage capacity to guarantee the truck refueling in case supply is interrupted. In contrast, large-scale storage facilities would need to keep sufficient reserves to act as a buffer to manage supply and demand between production plants and end users, something necessary for a hydrogen economy [44,139]. Table 26 presents the storage parameters at the production plant and the GotHUB. For the HRS the storage infrastructure was not included.

Table 26. Storage parameters at the production plant and at the GotHUB for each production pathway.

	Pathway A	Pathway B	Pathway C	Pathway D
	Production plant			
Storage method	Spherical vessel	Cryogenic dome	None. The pipeline itself works as storage	Cryogenic dome
Capacity (Kg H_2)	For grid powered cases: one 25 t usable capacity tank and 27 t total storage as defined by Papadias [83].<br><br>For wind-powered cases: four 25 t usable capacity tank and 27 t total storage as defined by Papadias [83].	One 230 t tank as described by Papadias [83] after NASA [84]	-	One 230 t tank as described by Papadias [83] after NASA [84].
Storage pressure	10.4 Bar	20 K, 20 Bar	-	20 K, 20 Bar
Material inputs	Total steel per tank is 1,306 t. Diameter: 39.5 m. Water volume: 32,000 m^3.Wall thickness: 34 mm. Steel density: 7,850 kg/m³	Total steel per tank is estimated as 367.167 t. Assumptions for a double hull tank with 2 cm wall thickness each shell. The outer diameter of the external shell is 20 m and the inner diameter of the internal shell is 18.56 m, representing a volume of water of 3400 m^3.	-	Total steel per tank is estimated as 367.168 t. Assumptions for a double hull tank with 2 cm wall thickness each shell. The outer diameter of external shell is 20 m and inner diameter of internal shell is 18.56 m, representing a volume of water of 3400 m^3.
Carrier dataset (Ecoinvent)	steel, unalloyed	steel, unalloyed	-	steel, unalloyed

Life expectancy	1.09E5 t H_2 stored throughout the lifetime. Lifetime of 30 yr was assumed. The amount of H_2transitting was assumed as 10 t day^{-1}.	2.92E5 t H_2 stored throughout the lifetime. 40 yrs [146]. A conservative stored amount per day is assumed as 50 t day^{-1}.	-	2.92E5 t H_2 stored throughout the lifetime. 40 yrs [146]. A conservative stored amount per day is assumed as 50 t day^{-1}.
Boiloff rate	0.3% [19]	0.04 % day-^{1} [40]. 6 days	-	0.04 % day-^{1} [40]. 6 days
	GotHUB			
Storage method	-	-	LRC	Cryogenic dome
Capacity (t H_2)	-	-	640 t (working capacity), 670 (total storage).	2 tanks of 230 t as described by Papadias [83] and NASA [84].
Storage pressure	-	-	170 Bar, average pressure	20 K, 20 Bar
Material inputs	-	-	The water volume is 40,000 m^3. Cylindrical shape is assumed as in Papadias [83] which results in a length of 588.45 m. A 15 mm liner as in Papadias[83] results in a liner of 448 m^3. Total steel use is 3,516.8 t for a steel density of 7,850 kg m^3. Assumed LRC lifespan is 40 yr and 300 t H_2 stored per day.	Total steel per tank is estimated as 367.168 t. Assumptions for a double hull tank with 2 cm wall thickness each shell. The outer diameter of the external shell is 20 m and inner diameter of internal shell is 18.56 m, representing a volume of water of 3400 m^3.
Carrier dataset (Ecoinvent)	-	-	steel, unalloyed	steel, unalloyed
	Hydrogen refueling station			
Storage method	CH_2 Cascade flasks	LH_2 Cryogenic dome	CH_2 Cascade flasks	LH_2 Cryogenic dome
Capacity	2 t	2 t	2 t	2 t
Storage pressure/ temperature	900 Bar	20 K	900 Bar	20 K

4.3.1. Gaseous state

Large-scale storage is the only element in the gaseous supply chain that is still not commercialized on large scales as of 2022, despite already being a proven concept [40]. There are alternatives for storing CH_2 already available on the market, but most commercial solutions are for smaller volumes, below 1,000 kg. For large-scale applications, including geologic storage, much of the technology today is still in development [40,83].

The technologies for large-scale CH_2 storage have low energy density as pressures are low, rarely exceeding 200 bar [40]. Spherical vessels display large capacities of up to 25 t [40,83] but are costly and bulky [18] and therefore, inadequate for HRS. Instead, spheres are a suitable option for storage at the city gates and the production facilities [83] and therefore, they are the selected method for storage at the centralized production plant. For the GotHUB we proposed lined rock caverns (LRC).

The spherical vessel in the production plant is based on the specifications of Papadias & Ahluwalia [83]. Storage capacity is 27 t of hydrogen while cushion capacity is 2 t. The sphere has a water volume of 38,000 m^3 which implies an energy density of around 26 kWh m^{3-1}, when the cushion gas is not included. Storage pressure is 10 Bar and wall thickness is 0.034 m while steel is the construction material. Table 26 exhibits the estimated material inputs for the spherical vessel while the energy consumption for compression was displayed in Table 19 and Table 21.

4.3.1.1. Lined rock caverns

Underground systems for storage of hydrogen include porous underground storage (referring to depleted oil and gas fields and aquifers), salt caverns and lined rock caverns (LRC) [83,175–177]. These geologic storage methods are expected to be the most financially competitive option for storage of large amounts of hydrogen, while also being orders of magnitude larger than pressurized vessels or cryogenic tanks, exhibiting capacities in the order of thousands of tons, for salt caverns, and of hundreds of tons, for LRCs, depending on the working pressure [83].

Salt caverns are carved in underground salt deposits by dissolving a part of the salt dome with water and subsequently extracting the brine, leaving behind an artificial chamber in the salt deposit [40,178]. Hydrogen storage has been demonstrated in salt caverns [179,180] but not in depleted fields or aquifers, which require a caprock to seal the hydrogen. The high diffusivity of hydrogen and the potential interactions with dissolved hydrocarbons and microbes are a source of concern for porous underground storage options [83]. Moreover, salt caverns are expected to affect hydrogen purity and hence would require an extra purification stage when intended for fuel cell use [40].

The largest storage capacity is theoretically exhibited by porous storage methods [83,176]; however, feasibility is still to be proven. Salt caverns show lower capacities, but as a proven technology, they appear to be a competitive option to be used in supply hubs like the GotHUB. Nonetheless, the underground salt deposits, in which the chamber is carved, are not found in Sweden [181], and as a result, salt cavern storage is not explored in this study. In contrast, due to the presence of metamorphic rocks, LRCs are a feasible option for the country [182].

In fact, one of the first LRCs intended for hydrogen is being built as part of the HYBRIT initiative in northern Sweden, where a section of an old mine is utilized [183]. Another case is found in Skallen but it is intended for NG, instead of hydrogen [184]. The energy density of LRC's is larger than that of salt caverns but it is also more expensive to construct [40] since the caverns are constructed through usual mining operations [176]. Once the cave is completed, a tight storage medium is built by cladding the cavern with a liner, that can be made of a polymer membrane or thin steel sheets. A theoretical unlined rock cavern would require a rock with low permeability, with no fractures [83].

LRCs operating pressure is expected to range between 20 bar and 250 bar [83,148,176]. For storage, hydrogen needs to be compressed first to reach the pressure inside the LRC, which in turn varies depending on the current mass of H_2 stored. In this study, and for the sake of simplicity, we assume the hydrogen is to be compressed to an average pressure of 135 bar, in a similar approach to Vendt & Wallmark [148]. A literature gap was identified as no examples of LCA for LRCs were found in our review and we found no data for the construction requirements of LRCs.

Despite not being able to include the construction of infrastructures, which comprise the excavation of the LRC itself, the surface facilities, the access tunnels and access shaft [176], we include an estimation for the liner and the energy requirements for compressing the hydrogen, see Table 26. Aiming to properly perform an LCA for the LRCs, it would be necessary to define the cavern dimensions, the amount of material for the lining and the excavation technique need to be defined. The conclusions are not extrapolable, but for salt caverns, Hystories[185] suggested that construction represent the largest share of GWP impacts with drilling of the cavern and building of surface facilities as the main contributors.

Excavation methods could be drilling or blasting [83,176]. To determine the optimal depth and location for an LRC associates site investigations and observing several factors, including safety, intended storage capacity and gas pressure and rock properties [176].

4.3.2. Liquid form

As of 2022, the storage of hydrogen in liquid state was the only mature technology available for large scales [40]. The world's largest LH_2 storage belongs to NASA, and it stores approximately 460 t in two spheres [84]. Cryogenic spherical tanks are employed as this is the optimal shape for minimizing surface area in relation to its volume, minimizing the heat transfer through the walls [40]. LH_2 tanks use multi-layered walls, one of which is a vacuum layer, that reaches more than one meter thickness in large tanks, as this is an effective way to limit heat transfer [40]. Wall thickness is lower for smaller vessels which leads to larger boil-off rates which were reported to reach an astonishing 15% in the past [186].

However, there is a trend of diminishing boil-off. Linde [187] reports about small-scale storage units with a capacity between 400 – 4,600 kg with boil-off rates of 0.5-1% per day. Furthermore, Linde has announced cylindrical tanks able to store up to 19 t of LH_2 whereas a spheric model can store around 70 t with 0.1 % boil-off. Similar products are being offered by gas management companies [40].

Although advantageous from an energy density perspective, the energy required for liquefaction will likely make LH_2 storage unfeasible for distributed production of hydrogen since small-scale liquefaction equipment will face great difficulties for cost reduction, and energy-efficiency enhancements. Furthermore, proximity between the LH_2 storage and the liquefaction plant makes liquefaction of boil-off easier [40].

Storage in liquid form was included for pathways B & D. For storage of hydrogen at the production plant and at the GotHUB we included a cryogenic tank as described by Papadias & Ahluwalia [83]. The infrastructure assumptions are included in Table 26.

During storage at the GotHUB it was assumed that active cooling was required to reliquefy the produced boil-off. A consumption of 4.07 kWh kgH_2^{-1} was considered [163] with a 0.1% boiloff.

4.4. Purification

The purification process mentioned in this section should not be confused with the process taking place at the end of the SMR plant, where BH_2 is produced. It is true that the technology for both processes is pressure swing adsorption (PSA) but the supply chain stages are different. The purification within the BH_2 production plant was already included in the system boundaries defined by Antonini et al.[99] and obtains its energy from the combustion of fuel within the SMR plant.

Instead, this process refers to the purification required to guarantee that the hydrogen complies with fuel cell purity requirements after it has travelled through the pipeline, considering that pipelines are not favorable to the ultra-high purity requirements of fuel cells, and reportedly, it is inevitable for the purity to be affected [40,49,76,143]. A similar problem would have happened if salt caverns had been considered for underground storage in this study [40].

PSA works by circulating a mix of gases through an adsorbent which selectively captures a given species from the blend while letting the others pass through; the adsorbent is usually a zeolite or activated carbon [78]. Inside the adsorption column, the high operating pressure forces the selective adsorption of the targeted species, and therefore, the main flow of gas leaving the system is enriched in the desired component, while the secondary stream contains all the species that were not adsorbed [76–78].

PSA is the standard technology for purification in SMR plants since it is reliable and can separate the hydrogen from the CO_2, CO and CH [82], which are common in the process and come from the NG used as feedstock. In contrast to SMR, the contaminants found in electrolytic production are mostly moisture, which is removed in a drying stage, while small traces of N_2 appear if the fuel cell obtained the oxygen from the air [49]. Electrolytic hydrogen in this study was assumed to be fuel cell grade, as in common in modern systems [76].

PEM fuel cells demand a 99.97% purity [46,77,78,96,98] to avoid poisoning of the platinum catalyst driving the reaction [76]. Carbon monoxide is especially harmful as it can deactivate the catalyst, degrading the entire fuel cell. Other contaminants are not directly detrimental to the fuel cell but can compromise the integrity of the tank [76,77]. In contrast, purification is not required for hydrogen used in ICETs as engines do not necessitate high purity.

Life cycle cost studies have estimated that, although the capital costs of the purifier could be high, the operating costs of PSA are not significant when the pressure for the process is obtained from the SMR pressure [76]. As our proposed pathway purifies the hydrogen right after the LRC, the hydrogen would be already pressurized beyond the working requirements, thereby any needs for compression would be provided by the LRC's compression system. Commercially available PSA systems are able to purify volumes of around 1,000 Nm^3hr^{-1} working at a pressure of around 7 bar [188189] but working pressures vary depending on the purifier's maximum flow.

Furthermore, hydrogen losses in PSA might be a reason for concern since the purging in the PSA process associated to SMR has been reported to be as high as 10% [76]. However, whether this value is extrapolable is unclear, experts consulted by Frazer-Nash [49] consultancy indicated that this value would be lower in other scenarios as consequence of the much lower range and concentration of impurities achieved by technologies different to SMR. More details on hydrogen leaks are found in the leakages section in the sensitivity analysis.

For this study we assumed the PSA energy consumption of 3.6 MJ Kg H_2^{-1}as in Ligen et al [149]. Moreover, there is a lack of data for the PSA system itself. However, neither the adsorber nor the pipes and valves are expected to be significant environmental footprint contributors. Purification was selected to happen at the GotHUB. This is advantageous since it allows larger efficiencies associated to economies of scale. Furthermore, the hydrogen in the distribution tube trailers will be pure, avoiding the risk of hydrogen embrittlement, potentially caused by impurities [52].

4.5. Refueling station

Refueling stations are characterized by the physical state of hydrogen, gaseous or liquid, when it is supplied to the station and when it is transferred into the vehicle. Depending on the physical state of storage the HRS configuration varies [45]. Both CH_2 and LH_2 technology exhibit advantages and disadvantages while the technology readiness levels are very different [40]. Compared to HRS with LH_2 or cryo-compressed refills, CH_2-based stations are well established and understood.

As of 2022, there were about 540 operational HRS globally [190] while only one offered refiling in liquid state [40]. An expansion in the number of HRS in Sweden is expected as around 100 HRSs are planned to be built in the coming years [191] while EU and Klimatklivet funding will allow the construction of 15 HRSs in northern Sweden [148].

Irrespective of the production location or the physical state in which hydrogen is supplied, both gaseous and liquid HRSs present common components necessary for the refueling process, namely: Compressors or liquefiers for packing, CH_2 or LH_2 storage tanks, safety valves, sensors and fire extinguishing equipment, and dispensers. [45]. In contrast, the CH_2 HRS requires a cooling unit to ensure the tank temperature does not surpass 85 C during refill by keeping the CH_2 at -40 C [22,45,53,73].

While HRSs supplied by centralized plants can be configured to use CH_2 or LH_2, HRSs producing hydrogen on-site are very likely to exclusively offer hydrogen in gaseous state as the production units supply it in this physical state and small-scale liquefaction is costly [40,146]. Moreover, it is technically feasible to supply a HRS with LH_2 for subsequent vaporization and refill of vehicles with CH_2 or cryo-compressed hydrogen [15]. Cryo-compressed refueling is not included in this study.

4.5.1. Gaseous refilling

Almost all currently operational HRSs provide CH_2 to their customer vehicles at pressures of either 350 or 700 bar. Hydrogen storage at 700 Bar offers quicker refills and larger amounts of hydrogen stored in the tanks, in contrast, refills at 350 Bar require less energy for compression, less robust tanks and precooling at -40 C is not necessary. [45]

There are two layouts for HRS supplying CH_2: cascade refueling and direct refueling with booster compressor [45], henceforth labeled as booster compressor. The HRS layout include CH_2 storage (where storage tanks pressure varies depending on the layout), compressors, and high-pressure storage (if cascade storage is implemented), and a pre-cooling phase prior to transferring the hydrogen into the vehicle's tank [45]. The cascade refueling layout is beneficial due to its simplicity and HRS reduced cost [45]. Conversely, the booster compressor

configuration offers flexibility, and it is recommended for HRSs that need to deliver hydrogen at both 350 and 700 Bar[45].

Cascade refueling contains a series of high-pressure tanks which operate at different pressure levels, with one tank at the highest pressure, while others are at high-medium and high-low pressures. The cascade layout allows for rapid refills [45,96], even for vehicles with large fuel tanks, however, it comes at the expense of requiring large cascade configurations. As the number of tanks increases, the pressure after each refill falls more slowly, enabling the refueling of more trucks while saving compression energy [45]. Thereby, as storage capacity is expected to be large, cascade HRS for heavy-duty vehicles are anticipated to have higher costs when compared to light-duty refueling [40]. Still, the optimization of the storage pressure levels reduces the overall energy consumption [45].

After the refill is finished, the amount of H_2, and the pressure inside the cascade storage, will have diminished. If the hydrogen level is too low, refilling other vehicles will not be possible [40]. Thereby, the cascade storage needs to be constantly refilled by compressors to maintain the pressure and the amount of hydrogen inside. These multistage compressors, usually diaphragm compressors or mechanical boosters, take hydrogen from the supply pressure up to the cascade storage pressure of more than 900 Bar [15,45]. Subsequently, when the customer truck connects to the HRS, the gas flows from the high-pressure cascade storage, passing through a refrigeration unit, to the lower pressure in the vehicle tank. A dispenser is required for transferring the hydrogen into the vehicle. The SAE J2601 standard dictates the refueling procedure and describes the equipment required performance. For instance, it states that during refilling the hydrogen pressure should not exceed 875 bar while temperature must oscillate in the range of -40 C to 85 C [40].

In contrast to cascade refueling, the booster compressor layout stores hydrogen at a medium pressure of approximately 400-500 Bar, after an initial compression stage. Subsequently, hydrogen is taken from the medium pressure storage and compressed and delivered directly to the truck tank by using booster compressors. Aiming to prevent the issues associated with pressure pulsation phenomena, this process requires high-pressure buffer tanks [45].

For this study, the HRS is based on a cascade layout; the energy required for compression to 900 Bar is shown in Table 27. The HRS infrastructure requirements for the HRS depend on the specific HRS setup and are not included in this study.

Table 27. Energy consumption and storage parameters at the refueling station for each transportation pathway.

	Packing-storage (kWh/kgH_2) Low and high	Transfer to storage (kWh/kgH_2)	Storage LH_2	Storage CH_2	Pre-cooling (-40C) (kWh/H_2)	Pumping to truck (kWh/kgH_2)
A	950 Bar<br>0.36 0.43	-	-	Cascade flasks 900 Bar	0.63	-
B	Active cooling 0.0407	0.08	Cryogenic tank 20 K	-	-	0.55
C	Till 950 Bar<br>1.46 1.75	-	-	Cascade flasks 900 Bar	0.63	-
D	Active cooling 0.0407	0.08	Cryogenic tank 20 K	-	-	0.55
A	Till 950 Bar<br>0.36 0.43	-	-	Cascade flasks 900 Bar	0.63	-
B	Active cooling 0.0407	0.08	Cryogenic tank 20 K	-	-	0.55
C	Till 950 Bar<br>1.46 1.75	-	-	Cascade flasks 900 Bar	0.63	-
D	Active cooling 0.0407	0.08	Cryogenic tank 20 K	-	-	0.55

4.5.2. Liquid refilling

Refilling vehicles using LH_2 does not require high pressures as in the case of CH_2. The main component is the liquid hydrogen pump which transfers the LH_2 from the storage tank, via the dispenser, into the truck at pressures of around 10 Bar [40], while the storage tank displays a pressure between 2 and 8 Bar [15,45]. Compared to the energy needed for liquefaction, the pumping of LH_2 uses little energy by comparison [15,96]. During the refill process the hydrogen is pumped but not vaporized.

The LH_2 supply is expected to display a higher efficiency compared to CH_2 supply [15,40]. However, the large temperature differences between the storage tank and the transferred LH_2 might cause large amounts of boil-off and the need for venting. A mitigation strategy would be to fill the tank partially when it is warm [40].

The energy consumption for the LH_2 in the HRS is presented in Table 27. The HRS infrastructure requirements for the HRS depend on the specific HRS setup and are not included in this study.

5. Use phase & end of life

5.1. Hydrogen consumption

To define an energy consumption per km adequate for representing average driving conditions in Sweden and the EU, for FCTs and ICETs, we appealed to the literature, to our own estimations, and to discussions with automotive industry experts[35,192]. Table 28 displays the hydrogen consumption estimations by all methods and the values selected for this study. We identified a lack of research exploring how fuel consumption in hydrogen-powered long-haul trucks is affected by real world driving conditions including peak power requirements and environmental conditions, *e.g.* temperature and humidity. This literature gap could be explained by how scarce these vehicles still are. Therefore, our consumption data relies on estimations and not on experimental results.

Table 28.Hydrogen consumption in FCTs and ICET according to literature review, discussions with experts and own assumptions

Reference	FCT (kWh 100km^{-1})	ICET (kWh 100km^{-1})	Notes
ICCT [13]	238.3 - (scenario 2021)<br>~ 7.15 kg H_2 100 km^{-1}<br>222.2 - (scenario 2030)<br>~ 6.66 kg H_2 100 km^{-1}	-	Load factor undisclosed
Marcinkowski et al. [193]	220.4 - (conservative scenario)<br>~ 6.61 kg H_2 100 km^{-1}<br>193.6 - (aggressive scenario)<br>~ 5.80 kg H_2 100 km^{-1}	-	Based on EPA 55 Cycle Trucks loaded at: 36,000 lb or 19,329 kg
VolvoTrucks [194]	333.3 - (Press release)<br>~ 10 kg H_2 100 km^{-1}	-	Estimation based on 80 kg H_2 onboard and 800 km range.<br>Load factor undisclosed.
ICCT [195]	262.6 - (scenario 2022)<br>~ 7.87 kg H_2 100 km^{-1}<br>184.6 - (scenario 2030)<br>~ 5.54 kg H_2 100 km^{-1}<br>173.6 - (scenario 2040)<br>~ 5.21 kg H_2 100km^{-1}	-	Representing group 5 – Vecto for long-haul vehicles.<br>Load factor: 63% equivalent to payload: 19,300 kg.
Daimler [196]	280.1 - (Press release)	-	LH_2 truck. Estimation based on 88 kg H_2 onboard and 1,047 km range.<br>Load factor undisclosed.
Discussions with experts. [35]	200 -<br>~ 6 kg H_2 100 km^{-1}	233.3 -<br>~ 7 kg H_2 100 km^{-1}	Load factor undisclosed
Man [197]	-	311.1 - (Press release)	Estimation is based on 56 kg H_2 and 600 km range.<br>Load factor undisclosed.
Own estimation	246.6 - (plain terrain)	304 - (plain terrain)	Estimation based on Equations 5 to 9. (Table 29)
This study	266.6 - (base)<br>~ 8.5 kg H_2 100 km^{-1}<br>200 - (improved)<br>~ 6.0 kg H_2 100 km^{-1}	311.1 - (base)<br>~ 9.3 kg H_2 100 km^{-1}<br>233.3 - (improved)<br>~ 7.0 kg H_2 100 km^{-1}	

Our own estimation of the hydrogen consumption, intended for comparison and validation purposes, is based in the power requirements per km as defined in Löfving et al. [198]; see Equation 5. This estimation covers the power requirements associated with rolling resistance (Equation 6), air resistance (Equation 7) and inclination (Equation 8). Finally, we estimated the energy requirements per km for both powertrain technologies (Equation 9) considering powertrain efficiencies presented in Table 29.

$$Pkm = Proll + Pair + Pincl \quad \text{Equation 5}$$

$$Proll = Croll * (mtruck + mcargo) * g * v * \cos\theta \quad \text{Equation 6}$$

$$Pair = \left(\frac{1}{2}\right) * \rho * A * Cd * v^3$$ Equation 7

$$Pincl = (mtruck + mcargo) * g * v * sin\,\theta$$ Equation 8

$$Ekm = \frac{Pkm}{\eta FC * \eta tdriveline * \eta BOPFC} * \frac{d}{v}$$ Equation 9

Aiming to show the variability of power requirements and energy consumption on demanding conditions Table 29 includes an estimation for a steep road, in addition to the plain highway section. For the plain road a driving speed of 80 km hr^{-1} was assumed, which is representative of a highway environment in the EU. Highway driving has been determined to represent most of the truck's lifetime [199], comparatively spending very few time in urban environments. For the sake of simplicity, we assume that every FCT model displays the same hydrogen consumption and the same applies for all ICET models. For FCTs, regenerative braking was assumed to recover 10% of total energy requirements. A 45% combustion engine efficiency was assumed for ICETs, in line with Wei et al. [200].

FCTs hydrogen consumption is influenced by the energy management strategies, truck weight, driving speed, and road inclination [201]. Energy management strategies are crucial for FCTs as fuel cell efficiency decreases at high loads, requiring LiBs to provide additional power when demanding conditions are found. In fact, energy management is not only relevant for the hydrogen consumption but also for the fuel cell life expectancy [193,201].

Table 29. Parameters used and results obtained from the hydrogen consumption estimation.

	FCT		ICET	
	Low inclination	High inclination	Low inclination	High inclination
$Croll$	0.0055			
g (m s^{2-1})	9.8			
A (m^2)	10			
Cd	0.6			
$\rho\ air$ (kg m^{3-1})	1.2			
$mtruck$ (kg)	17,500		18,000	
$mcargo$ (kg)	24,500		24,000	
v (km hr^{-1})	80	40	80	40
θ (degrees of inclination)	0	4	0	4
ηFC / ηICE	0.6	0.5	0.45	0.48
$\eta driveline/transmission$	0.93		0.9	
$\eta BOPFC$	0.9	0.9	-	-
d (km)	1			
v (km hr^{-1})	80	40	80	40
$Power$ (kW km^{-1})	99	410	99	410
$Energy$ (kWh km^{-1})	246	2452	304	571

The peak power demands linked to steep road sections cause truck speed to significantly decrease compared to the plain highway sections. Despite this reduction, energy requirements spike abruptly on such road sections as shown in Table 29. In real world driving conditions, the batteries in FCTs and the gearbox in ICETs will cope with peak demand. A more precise estimation of hydrogen consumption under high power demand conditions is beyond the scope of this study. Moreover, we did not consider the amount of hydrogen that remains within the tank. Such cushion hydrogen is kept in the tank to guarantee structural integrity and keep minimum pressures [22].

5.2. AdBlue consumption

The SCR systems for hydrogen engines are not expected to include particle filters or oxidation catalysts. However, SCR systems for hydrogen engines will also use AdBlue as working fluid.

For AdBlue consumption we adopted the Joint Research Centre[16] estimation for dual fuel (LNG+B7) compression ignition engines (0.012 L kWh^{-1}), due to the lack of data for hydrogen-propelled engines. A more

precise estimate would require data about the NO_x engine out levels which would depend on the specific configuration of the engine. For AdBlue manufacturing we assumed a composition of 1/3rd of urea and 2/3rds of deionized water.

5.3. Tailpipe emissions

N_2O tailpipe emissions are of particular interest from a GWP point of view. Despite the incorporation of after-exhaust systems based on SCR, some formation of NO_x and N_2O is expected, mainly during engine start-up when the SCR system has not reached steady working conditions yet. Regardless of how those emissions are treated the vehicle must comply with the restrictions enacted by EURO 7 standards [202,203].

Due to the lack of primary data representing the tailpipe emissions of a hydrogen 350 kW HDPI engine we conservatively adopted the N_2O emissions estimated for hot operation conditions by the Joint Research Centre [8]. The tailpipe emissions for hydrogen ICETs are presented in Table 30.

The presence of carbon-related emissions in the combustion of hydrogen should be negligible due to the absence of any carbon in the hydrogen. However, as HDPI engines require pilot fuel for initial combustion, a 2% of biodiesel is blended with the hydrogen, in the combustion chamber. The carbon contained in the biodiesel was assumed to be present complete stoichiometric combustion. CO_2 emissions from biodiesel combustion were considered as 76.2 g MJ^{-1} [16]. The upstream production of biofuel was modelled according to the Ecoinvent 3.8 dataset "*esterification of soybean oil | fatty acid methyl ester | US-RS*".

Likewise, the presence of engine lubricants is expected to result in small emissions of hydrocarbons and CO. Thus, the lubricant is the source of CO and NMVOC, assumed to reach the EURO7 limits and depicted in Table 30. In contrast, FCTs emissions basically consist of condensed water. Hydrogen venting could be required for both FCTs and ICETs and is included in the hydrogen leakages analysis.

Table 30. Tailpipe emissions for the ICET. High and low values.

		NO_x	CO	NMVOC	N_2O
mg kWh^{-1}	EURO 7 limits	260	1,950	105	260
	Hot operation				90

Finally, both ICETs and FCTs create on-road particulate matter emissions from tire and brake wear. Since regenerative braking reduces the burden of friction in FCT brakes, emissions are expected to be smaller compared to ICET cases [14]. A recent study estimated the national tire wear emissions [204] based on emission factors [205] derived from older studies [206]. However, due to the lack of precise data presenting non-tailpipe emissions specific for long-haul FCTs and ICETs we did not include them in the study.

5.4. Maintenance & truck lifetime

The maintenance and repair activities necessary for the refueling infrastructures at the HRS were excluded from this study and only truck maintenance was included. Omitting the HRS equipment maintenance is not expected to cause a significant distortion in the results as such impacts are expected to be negligible, as stated by Weiszflog & Abbas [22], after discussions with Volvo experts.

In the past, the environmental footprint of vehicle maintenance has been evaluated. However, it has not been estimated to be a significant GWP contributor to total impacts per km or per tkm. Scania has estimated the GWP impact of maintenance [207] to be as low as 0.3% (excluding tires) for BETs with a payload of 6.1 t. The authors also highlighted the difficulty of defining average values for maintenance-related emissions due to the variability of vehicle operation. Furthermore, a low GWP contribution caused by maintenance, to total results per tkm, was also estimated by Ricardo [10].

For modelling the maintenance in 40 t FCTs we assumed one LiB replacement throughout the vehicle lifetime, a conservative assumption based on discussions with automotive experts[35] while for lead-acid batteries a single replacement was included, as in GREET 2022 [27]. In this study, we consider the replacement LiB to be the same as originally included in the vehicle. Tires replacement along the truck lifetime was also included as in GREET 2022 [27]. FCTs and ICETs were assumed to have the same number of tires and replacement rates

are identical. Table 31 exhibits the items included in the maintenance plan. No fluids were included in the analysis.

Table 31. Maintenance stage parameters

Item	Replacement	Notes
Li-Ion battery	1 battery, 1 replacement	As defined in the vehicle section
Lead-acid battery	1 battery set, 1 replacement	GREET 2022
Steer tires (tractor)	2 tires, 7 replacements	120 lb (90% rubber, 10 steel). GREET 2022
Drive tires (tractor)	8 tires, 3 replacements	
Trailer tires (trailer)	8 tires, 10 replacements	

Vehicle life expectancy for all truck models was considered as 1,000,000 km. This is a conservative assumption compared to other studies: 1,050,000 km [17], 1,243,000 km [13] and 1,000,000 mi (1,609,000 km) [193]. Fuel cell lifetime was assumed to expand throughout the entire vehicle lifetime.

5.5. End of life

Vehicle end of life includes the manual dismantling and transport of subcomponents to a recycling facility 25 km away. We appealed to *Used bus* | *Row* process at Ecoinvent 3.8. Results are small and added to truck manufacturing.

6. Sensitivity analysis

Given the rapid technological advancement that has been anticipated for all stages of the hydrogen supply chain we performed a sensitivity analysis to determine how variations in key input variables affect the LCA outcomes. The parameters incorporated in the sensitivity analysis aim to model the current performance of the processes up to their expected state in 2030 and are depicted in Figure 4 of the manuscript.

The first set of variables incorporated in the sensitivity analysis refer to improvements in the modelled supply chains. This increase in efficiency translates into reductions in energy use over the different stages of the supply chain.

For the hydrogen production stage we evaluated how improvements in process efficiency, represented by electricity consumption for electrolysis and feedstock consumption for NG SMR and Biomethane SMR, influence the LCA outputs. Furthermore, we performed similar analyses for the transportation stage by assessing the variations created by improvements in compression and liquefaction (also known as packing) efficiencies, which translate into electricity consumption decreases. In addition, for the use phase we varied the fuel consumption per km, associated to driving the fully loaded truck, for both FCTs and ICETs. Also, for the ICET we evaluated how the N_2O content in the tailpipe emissions vary.

Furthermore, we explored the impact of hydrogen leaks over the whole system boundaries. We incorporated the lower and upper estimated hydrogen leakages throughout based on a literature review as shown in Table 32. See section-6.1.

Moreover, the second set of variables intends to explore substantial changes in the technologies used for hydrogen production or design changes onboard the trucks. This is represented by the following cases: 1) One scenario where the type IV tanks, intended for storage of gaseous hydrogen at 350 Bar and 700 Bar, are manufactured using recycled carbon fiber; 2) One scenario where the steel used for truck manufacturing is obtained via direct reduction of iron (DRI) using GH_2.So far in the study, all steel and inputs are modelled by datasets representing European or global markets instead of representing a Swedish manufacturing.

6.1. Hydrogen leaks

Despite being economically detrimental, some fraction of hydrogen inevitably leaks during production, transportation, storage, and use [18,40,49,96,208–210]. Recent evidence suggests that hydrogen behaves as an indirect GHG due to its impact on atmospheric chemistry [50,51].

While hydrogen itself does not absorb infrared radiation, and thus, it does not act as a direct GHG, it reacts with hydroxyl radicals in the atmosphere [50,51]. Such alteration is associated to four climate impacts, namely: 1) an extended methane lifetime, 2) an enhanced production of tropospheric ozone and changes in stratospheric ozone; 3) an increase in stratospheric water vapor, and; 4) changes in aerosol production [50,51]. Warwick et al. [50] estimates hydrogen's GWP_{100} at 11 ± 5 whereas Sand et al [51] estimates it at 11.6 ± 2.8. This suggests that hydrogen leaks might offset some of the anticipated climate benefits of a hydrogen-based economy. For this study we adopted the values in Sand et al [51] for GWP_{100} meaning that lower and upper values are 8.8 and 14.4 respectively.

Nonetheless, there is limited data on the precise magnitude of the leakages in developing hydrogen supply chains [51] and current estimations vary roughly [49,208,209]. A recent study by Esquivel Elizondo [210] synthesized the hydrogen emissions reported by past studies and served as a basis for our study. Furthermore, Esquivel Elizondo [210] also classified the estimations based on how they were obtained: Lab experiments, assumptions, calculations and simulations. For this study we prioritized laboratory results, followed by simulations, then calculations and finally assumptions, in that order. Table 32 presents the lower and upper end of the hydrogen leaks included in this study.

In addition to the potential GWP linked to the hydrogen released into the atmosphere, leakages also represent a net hydrogen loss, meaning that, for every kg of hydrogen being refueled into the truck, a larger amount of hydrogen would need to be produced to make up for the losses. In other words, at each stage, a certain fraction of the gas is lost, and the remaining fraction moves to the next stage. If the loss fraction at stage r is Lr the fraction retained is $1 - Lr$.

The total amount of leaks is the difference between the initial amount of gas and the remaining gas as described in Equation 10 while the total estimated leakages for are presented in Table 33. No variations between the 350 Bar and 700 Bar cases were included due to the lack of data on how leakages can vary as a function of pressures. In any case, such variations would exclusively arise at compression in the HRS and inside the vehicles.

$$Total\ leaks = 1 - (1 - Lr_1) \cdot (1 - Lr_2) \cdot \ldots \cdot (1 - Lr_n) \qquad \text{Equation 10}$$

Table 32. Hydrogen leakage rates included in this study for value chain components

Stage	Lower (%)	Higher (%)	Reference- (Estimation method)	Included in pathway	Notes
Green H_2 production (central)	0.52	4	Lower leaks as in Frazer-Nash [49] - (Modeling).<br><br>Higher leaks as in Fan et al.[211] - (Laboratory test)	A,B,C,D(Chile).	Lower leaks assume that full recombination of H_2 from purging and crossover venting has been implemented as estimated by Frazer-Nash [49] with a 99% confidence level. For higher leakages we assumed the value in Fan et al. [211] despite the study more closely representing an on-site production than a central plant. Leakages of electrolysis with no recombination could be as high as 9.2% Frazer-Nash [49]; however, these estimates are based on SMR plants and their subsequent PSA stage, which is not necessary for electrolysis. Indeed, estimates for leakages during electrolysis from other studies are much lower than in Frazer-Nash [49] as shown by Esquivel Elizondo [210].
Green H_2 production (on-site)	2	5	Lower leaks as in Fan et al.[211] - (Laboratory test)<br><br>Own assumption for Higher leaks	On-site	Individual leaks at distributed production facilities are expected to be more difficult to address than in centralized plants, and therefore, the leakage rates are expected to be higher. The adopted lower leaks correspond to lab experiments performed by Fan et al. [211] which represent small electrolyzers. For higher leaks we proposed a 5% value which aims to represent leakages that are more difficult to address than in the central plant case (4%).
Blue H_2 production	0.1	1	Cooper et al.[209] – (Calculation)	A,B,C,D(Norway).	Blue H_2 may have higher leakages than gray H_2 since it requires additional separation processes. PSA is a source of concern but in centralized plants we assumed there will be recirculation of the discarded stream of gas coming out of the PSA process.
Compression	0.14	0.27	Cooper et al.[209] – (Calculation)	A-Production plant & HRS<br><br>C-Production plant, GotHUB & HRS	We assumed the same H_2 leakage rates for all compressor sizes due to lack of data. Large scale compressors are expected to present lower leakages but accurately determining the magnitude of these emissions is beyond the scope of the study. Different types of compressors might have different leakage rates.
Liquefaction	0.15	2.21	Cooper et al.[209] – (Calculation)	B-Production plant & HRS<br><br>C-Production plant, & HRS. Active cooling leaks at GotHUB not-included.	Higher leaks have been estimated in older studies to be as high as 5% Frank et al. [18]. However, we adopted the estimates of Cooper et al.[209] for both lower and higher leaks as data is more updated. Potential leakages during active cooling were not included due to lack of data.
Storage-gaseous	2.77	6.52	Frazer-Nash [49] – (Modeling)	A-Production plant & HRS<br><br>C-GotHUB & HRS	It refers to above ground storage. We assumed the same leakage rate for storage at the production plant, the GotHUB and the HRS. This is a rough estimation as the size of the storage device and the storage time are critical for an accurate estimation of slips.

					There is little knowledge on leakages for these systems, especially when referring to large scale facilities [212].
Storage - liquid	0.3	5	Lower leaks as in Van Ruijven et al.[213]. Assumption.<br><br>Higher leaks assume 1% leakage per day according to Frazer-Nash (2022).	B-Production plant & HRS<br><br>D-Production plant (Chile & Norway), GotHUB & HRS	Above ground storage. Van Ruijven et al.[213] assumed leaks of maximum 1% whereas Cooper et al.[209] estimated around half of that. Aiming to evaluate a more critical case we included the boil-off in Frazer-Nash[49] representing industrial facilities commercialized by Linde (max 1% per day). For storage time we assumed 5 days. We assumed the same leakage rate for storage at the production plant, the GotHUB and the HRS. This is a rough estimation as the size of the storage device and the storage time are critical for an accurate estimation of slips.
Storage in cavern	0.02	0.06	Frazer-Nash[49] – (Modeling)	C-GotHUB	Value estimated for salt caverns but included here to represent lined rock caverns due to the lack of data.
Transmission-road-gaseous	0.3	2.3	Lower leaks as in Frazer-Nash [49].<br><br>Higher leaks as in Fan et al.[211]	A-Transmission<br><br>C-Distribution	Lower and higher values from different studies aiming to cover a larger uncertainty range. Both Frazer-Nash [49] and Fan et al. [211] are based on modelling/calculations. Leakages were assumed to happen only during charge and discharge, and hence, the distance would be irrelevant for leakages estimation. This is a rough assumption
Transmission-road-liquid	3.76	5.5	Lower leaks as in Frazer-Nash [49].<br><br>Higher leaks as in Van Ruijven et al.[213] and Frank et al.[18]	B-Transmission<br><br>D-Distribution	Higher value was not taken as in Frazer-Nash [49] since it is based on a 5% leakage per day, which compared to the other studies in Esquivel Elizondo [210] is rather high. Instead, we chose the 5.5 reported by Van Ruijven [213] based on discussions with experts. For estimating the leakages in liquid H_2 transportation distance is crucial. As this is a rough approximation, estimating a precise value is beyond the scope of the study.
Transmission pipeline	0.04	0.48	Frazer-Nash[49] – (Modeling)	C-Transmission	Extrapolated from data representing the UK natural gas national transmission system (NTS).
Tanker ship transportation	0	2.3	Cooper et al.[209] – (Calculation)	D-Chile & Norway	The lower end assumes total recirculation of boiloff and, if required, flaring. It has been anticipated that boil-off in large tanks could be as low as 0.04% per day. Since transportation from Chile takes 20 days a 2.3 % leakage rate is expected to represent a safe approximation.
Purification	1	10	Lower leaks based on own assumption.<br><br>Higher leaks taken from Hy4Heat [76]	C-GotHUB	For lower leaks we assumed recirculation of discarded gas stream which contains H_2, CO_2 and others. Higher leaks based on PSA typically cause a ~10% loss, which could be combusted along with the feedstock for obtaining energy in large production plants. As this purification stage is located in the GotHUB we assumed there would be recirculation of the discarded stream of gas but not combustion for energy purposes.

Refueling-gaseous	2	3	Arrigoni & Diaz [208] – Assumption	A,C, On-site	Exact system boundaries were not disclosed by Arrigoni & Diaz [208].
Refueling-Liquid	2	8.5	Arrigoni & Diaz [208] – Assumption	B,D, On-site	Frank et al mentions high losses in the cryopump. Specific data is missing from the literature.
Fuel cell truck-gaseous	0.56	2.64	Frazer-Nash[49]	A,C, On-site	Duration of H_2 within the tank is crucial. 0.56% refers to full recombination of hydrogen from purging and crossover venting.
Fuel cell truck- liquid	1.12	5.28	Own assumption	B,D	Due to the lack of data, we considered the leakages would be twice as high as in the gaseous case. This is a rough assumption. An accurate estimation would be strongly dependent on how long the hydrogen remains in the tank which relies on the truck capacity factor and idling times.
Combustion engine truck	0.3	0.66	Frazer-Nash[49]	A,B,C,D	Overall, the hydrogen emissions from combustion of hydrogen in internal combustion engines are likely to be negligible and have not been included in this model. Leaks refer to storage and BoP.

Table 33. Total leaks per transportation pathway and type of vehicle. Values are expressed as a percentage of the total hydrogen produced.

Electrolytic pathways	Lo-A	Hi-A	SMR pathways	Lo-A	Hi-A
A-FCT	8.9%	21.8%	A-FCT	8.5%	19.4%
A-ICET	8.6%	22.3%	A-ICET	8.3%	19.9%
B-FCT-LH2	7.9%	30.6%	B-FCT-LH2	7.5%	28.4%
C-FCT	7.4%	26.5%	C-FCT	7.0%	24.2%
C-ICET	7.2%	25.0%	C-ICET	6.8%	22.6%
D-FCT-LH2	9.1%	35.6%	D-FCT-LH2	8.7%	33.6%
On-site-FCT	7.3%	16.4%			
On-site-ICET	7.0%	14.3%			

6.2. Direct reduction of iron

Direct reduced iron (DRI), colloquially known as sponge iron, refers to the product obtained after removing the oxygen found in the iron-containing feedstock by means of a reducing agent. Afterwards, DRI is loaded into an electric arc furnace (EAF) for steel production. EAFs can be used for both production of primary steel, as happens in this case, but also for recycling of steel from scrap. The production of DRI takes place exclusively within the solid phase, and thereby, no melting occurs [214,215]. In Sweden, one third of steel production was scrap-based in 2022 [216].

In contrast, in the conventional process for production of primary steel, pig iron is obtained in liquid state in a blast furnace (BF) and is then fed (melted or solid) into a basic oxygen furnace (BOF), where steel is produced. Although CO, coal gas, and other gas mixes could be used as reducing agents [215,217] we exclusively consider hydrogen as reducing agent in this case.

Depending on how hydrogen is produced and how the energy for DRI production is obtained, the entire process has potential for GWP mitigation in steel production. Studies have estimated that steel manufacturing integrated with hydrogen-based DRI could achieve approximately a 78% GWP_{100} reduction compared to a BF-BOF case [215]. Similarly, Rechberger et al.[214] suggested that up to 91% of the emitted CO_2 can be mitigated, when the process is fully run on renewable electricity, compared with a NG-DRI reference process.

For modelling the DRI production process we considered the data in Yilmaz & Turek [218] and Rechberger et al.[214], which includes NG inputs resulting on a carbon content of 5 kg C t_{DRI}^{-1}. The inclusion of NG is driven by the need to introduce carbon into the EAF process either to create a layer of slag to improve the energy efficiency of the melting process or to carburize the steel [214,219]. Since the melting process at the EAFs happens with no direct addition of carbon, its presence in the sponge iron or in the scrap may be too low for steel requirements.

Material and energy inputs for the DRI process are presented in Table 34. Afterwards, the DRI dataset replaces the scrap inputs in the EAF process. To represent this, we substituted the scrap inputs in Ecoinvent V 3.7 process *steel production, electric, low-alloyed* | *steel, low-alloyed* by DRI inputs. Additionally, we changed all electricity inputs representing other European countries by Swedish grid electricity. Then, this new dataset representing DRI-EAF steel production replaced the low-alloyed steel inputs described in the Vehicles section. More specifically, it replaced the primary steel in the trailer, combustion engine, transmission, FCT cooling system, body and chassis.

This decision was made as we deemed the complete substitution of steel used in the truck by DRI-EAF steel unlikely within the time scope of this study. Besides, we assumed that the substitution would happen initially in subcomponents that are manufactured directly by the OEMs and not outsourced such as fuel cell modules, battery modules, and tank systems. In addition, we assumed such substitution would likely begin with non-specialized steels like low-alloyed steel.

Table 34. Inputs for the DRI process.

Material	Value	Unit	Reference	Notes
Input				
Iron pellet	1.39	t	Rechberger et al.[214]	*iron pellet production* | *iron pellet* | *RoW*
Hydrogen	68.9	kg	Yilmaz & Turek [218]	*As in central production-grid*
Natural gas	8.3	kg	Yilmaz & Turek [218]	*Natural gas, high pressure, import from NO -SE*
Output				
DRI	1	t		
CO_2 (emission)	1.5	kg	Yilmaz & Turek [218]	

NG density: 0.7 kg Sm^{3-1}

As a fully hydrogen-based DRI process implied changes in the physical and metallurgical phenomena [220], a shaft furnace with pure hydrogen had never been operated before 2018 and required the complete redesign and testing of the plant. Hybrit, a joint venture between SSAB and Vattenfall achieved the feat of being the

world's first pilot plant producing fossil-free steel with a total yield of 5,000 t by the end of 2024 [221]. The full-scale process is expected to be ready only in 2035 [222].

6.3. Recycling of tanks

The manufacturing of carbon fiber (CF) is complex, and the specifics of the process are heavily defined by the features of the method applied [223]. For instance, the type of carbon fiber precursor used (cellulose, pitch, or polyacrylonitrile), the polymerization method employed (emulsion or dispersion), the temperatures and tensions applied to the fiber and even the spinning techniques [6].

Studies have estimated a carbon footprint reduction when CF is obtained from used fibers via recycling, compared to primary CF [6,22,223]. However, the environmental benefits of obtaining CF via recycling can only materialize if the recycled CF is adequate for use in applications where exigent mechanical properties are required, like in the case of type IV tanks [223].

The substitution of virgin CF by recycled CF should not be taken for granted as the mechanical properties of the recycled fibers are degraded, compared to the virgin version [6,22,223–225]. Technologies for recycling carbon fiber include mechanical, thermal (pyrolysis and fluidized bed), and chemical pathways [223–225].

Among the aforementioned methods, only pyrolysis and mechanical recycling exhibit advanced TRLs; 8 and 9 respectively [224]. Zhu et al.[225] argues that CFs recovered via pyrolysis lose between 15-50% of their tensile strength, however, those fibers are longer than those obtained by mechanical recycling, which is advantageous for the mechanical properties. More recently, Ren et al [226] investigated a pyrolysis-based method that maintains 99.4 % of the maximum tensile strength while recovering 96.5 % of the fibers. Moreover, Zhu et al.[225] claimed that fibers recovered via fluidized bed method lose between 25-90% of their tensile strength. In addition, the CFs recovered via mechanical recovery methods are not adequate for substituting virgin carbon fibers [223] and their tensile strength is 35-50% lower compared to virgin fibers. Due to the high TRL we chose pyrolysis as the most likely method to be used within the time scope of this study. We modelled the stages of recycling via pyrolysis as depicted by Weiszflog & Abbas [22].

Solvolysis stands out within the chemical recycling pathways. It employs solvents and alcohol to decompose the epoxy resin through a depolymerization reaction, separating the carbon fibers from the epoxy resin [223]. This method is efficient in retaining the tensile strength of the recycled fibers with losses of only 2-15%. However, this process involves using toxic substances (Zhu et al., 2019) and the technology used for this method is immature; Zhang et al [224] estimated a TLR 4.

In addition to exploring different pathways for recycling, reducing the amount of carbon in the tanks while keeping the mechanical properties is another line of research. Such methods include 1) changing the ply angle, which refers to altering the fiber angles for more efficient material use and even stress distribution; 2) patch reinforcement, which is the placement of patches to reduce the overall material needed and 3) the search for new materials with comparable tensile strength [227].

7. Biomethane comparison

This case represents an HPDI truck running on liquified biomethane (LBM), meaning that biomethane is used directly as a fuel instead of serving as feedstock for steam reforming. See Figure 6 in the manuscript.

The biomethane obtained via upgrading of biogas is expected to present a composition nearly identical to that of NG [99]. The LBM HPDI engine was assumed to be very similar to the hydrogen HPDI engine depicted in the hydrogen ICET cases in this study. In fact, fuel energy performance (kWh km^{-1}) is expected to be rather similar for both trucks ±2%[192]. For this study we assumed the fuel energy performance to be identical.

We incorporated the same biomethane production datasets already included in the steam reforming cases, described in the hydrogen production section, which describe the anaerobic digestion of residues and subsequent biogas upgrading with digestate application to the soil, for improved carbon sequestration [99]. To represent the absence of the CCS stage we adjusted the LCIs by linearly reducing the carbon sequestration proportional to the estimations for lower bound C sequestration, a rough approximation. Once the biomethane exits the upgrader it undergoes a liquefaction process for which temperature must reach −162 °C, at ambient pressure, as in the case of NG [228]. For LNG, liquefaction is the most energy-demanding stage in the supply chain [228]. Here we assumed the energy is obtained from the Swedish grid.

According to Pospíšil [228] the energy demand for NG liquefaction ranges between 5% and 15% of the total fuel energy, depending on the specific technology used for the process. When considering a biomethane LHV of around 13.8 kWh kg^{-1} [16] the energy requirements range between 0.69 kWh kg^{-1} and 2.08 kWh kg^{-1}. Indeed, some commercially available models claim an energy consumption as low as 0.7 kWh kg^{-1}[229]. We assumed an energy consumption of 1.0 kWh kg^{-1} as a more precise demand estimation depends on the specifics of the installation and is beyond the scope of this case. For the liquefaction facilities we employed the Ecoinvent 3.7. dataset *market for natural gas processing plant* | *natural gas processing plant* | *GLO* which is expected to process 1.2E12 m^3 of biomethane throughout its lifetime.

Truck modelling was similar to the hydrogen ICETs defined in the vehicles section only replacing the gaseous storage system with the liquid hydrogen storage system and the balance of plant included for LH_2 models. Maintenance was included as in the hydrogen ICET cases described in the maintenance section and a lifetime of 1,000,000 km was adopted. Liquefied biomethane density was considered as 0.750 kg m^{3-1} while gaseous biomethane density at the upgrader exit was considered as 0.72 kg m^{3-1}, both at standard conditions. The payload was assumed as 27 t.

Analogously to the H_2 ICET, we included base and improved energy consumption scenarios as described in the use phase. Tailpipe emissions for the truck running on biomethane are presented in Table 35. All species: CO, NMVOC, CH_4 and NOx comply with EURO 7 standards[203].

Table 35. Emissions and fuel consumption for the base case and the improved case of LBM.

Fuel use	kg km^{-1}	Notes
LBM-base	0.224	Base case. Equivalent to 3.11 kWh km^{-1}. See section 5.1
Emissions	g km^{-1}	
CO_2	614	Emission factor: 2.74 $kgCO_2$ kg LBM^{-1}. LHV: 13.88 kWh kg [16]
N_2O	0.28	Similar to hydrogen HPDI. Based on estimation of 90 mg kwh^{-1} during hot engine conditions [8]
CH_4	0.1	An estimated 0.032 g kwh^{-1} was obtained from on-road tests for spark ignited 40t trucks with 50% load factor [230].
CO	1	Estimated for short-Haul Truck CIDI - LNG on GREET [27].
NMVOC	5.14E-2	Estimated for short-Haul Truck CIDI - LNG on GREET [27].
PM	-	Considered negligible. [228]
CH_4	0.15	Non-tailpipe slips of methane. [230]. Although slips in HPDI engines could be lower, differences are not expected to be significant, based on discussions with experts.
Fuel use	kg km^{-1}	Notes

8. Results

8.1. Climate change

Table 36. GWP results, in kg CO_2eq per kg of produced hydrogen, for the production methods included in this study.

	GH_2		SgH_2		BH_2		BmH_2
	Central	On-site	Central	On-site	Low CH_4 leaks	High CH_4 leaks	Low C seq
Base	1,01	1,13	2,56	2,79	4,69	7,48	-7,81
Improved	0,93	1,03	2,36	2,56	4,56	7,10	-6,87

The cases labelled as "improved" represent the GWP variations expected from the efficiency improvements evaluated in the sensitivity analysis. GH_2 and SgH_2 depict cases of production in centralized and distributed. BH_2 cases are presented for low and high NG leakages while hydrogen from steam reforming of biomethane (BmH_2) includes a single case which depicts CCS + soil application of biodigestate.

Table 37. GWP results, in kg CO_2eq, for the entire maintenance stage over the entire lifecycle.

	FCT_{300}	FCT_{200}	ICET
Truck tires	14971,3	14971,3	14971,3
Battery pack	4636,2	15557,8	-
Lead-acid batteries	34,3	34,3	143,9
Total	19641,9	30563,4	15115,2

Table 38. GWP results, in kg CO_2eq, present in the ICET tailpipe emissions.

	Best	Base	Worst
Tailpipe N_2O	0,056	0,075	0,216
Tailpipe CO_2 -Biodiesel	0,008	0,011	0,011
Biodiesel (upstream)	0,003	0,005	0,005
Urea	0,011	0,015	0,015
Total	0,076	0,101	0,243

Contribution to GWP results by the ICET tailpipe emissions. The best case includes an improved hydrogen consumption (233.3 kWh $100km^{-1}$) and low N_2O emissions (90 mg N_2O kWh^{-1}). The base case represents a base hydrogen consumption (311.1 kWh $100km^{-1}$) along with low N_2O emissions. The worst case includes the base consumption with high N_2O emissions (260 mg N_2O kWh^{-1})

Table 39. GWP results in kg CO_2eq per kg of transported hydrogen. It refers to transport from the production equipment to the refueling nozzle, for the different T&D pathways and on-site production cases.

	A-grid	A-wind	A-grid 350 bar	A-wind 350 bar	B-grid	B-wind	C-grid-	C-wind-	C grid 350 bar	C wind 350 bar	C-grid-BH_2	C-grid-BH_2-350 bar	D-Chile	D-Norway	Onsite-700 bar-grid	Onsite-350 bar-grid	Onsite-700 bar-wind	Onsite-350 bar-wind
	Base case																	
Packing at plant	0,12	0,05	0,12	0,05	0,58	0,21	0,03	0,01	0,03	0,01	0,03	0,03	0,21	0,29	-	-	-	-
Transmission	1,29	1,29	1,29	1,29	0,43	0,43	9,4E-04	9,4E-04	9,4E-04	9,4E-04	9,4E-04	9,40E-04	3,99	0,28	-	-	-	-
Packing at GotHUB	-	-	-	-	-	-	0,06	0,06	0,06	0,06	0,06	0,06	0,01	0,01	-	-	-	-
Purification	-	-	-	-	-	-	0,04	0,04	0,04	0,04	0,04	0,04	-	-	-	-	-	-
Distribution	-	-	-	-	-	-	0,42	0,42	0,42	0,42	0,42	0,42	0,14	0,14	-	-	-	-
Packing at HRS	0,05	0,05	-	-	0,03	0,03	0,10	0,10	-	-	0,10	-	0,03	0,03	0,13	0,08	0,05	0,03
Total	1,46	1,39	1,41	1,34	1,04	0,67	0,66	0,64	0,55	0,54	0,66	0,56	4,37	0,75	0,13	0,08	0,05	0,03
	Improved case																	
Packing at plant	0,10	0,04	0,12	0,05	0,45	0,16	0,02	0,01	0,03	0,01	0,02	0,03	0,16	0,23	-	-	-	-
Transmission	1,29	1,29	1,29	1,29	0,43	0,43	0,00	0,00	9,4E-04	9,4E-04	0,00	9,40E-04	3,99	0,28	-	-	-	-
Packing at GotHUB	-	-	-	-	0,00	0,00	0,05	0,05	0,06	0,06	0,05	0,06	0,01	0,01	-	-	-	-
Purification	-	-	-	-	0,00	0,00	0,04	0,04	0,04	0,04	0,04	0,04	-	-	-	-	-	-
Distribution	-	-	-	-	0,00	0,00	0,42	0,42	0,42	0,42	0,42	0,42	0,07	0,07	-	-	-	-
Packing at HRS	0,04	0,04	-	-	0,03	0,03	0,09	0,09	-	-	0,09	-	0,03	0,03	0,13	0,08	0,05	0,03
Total	0,10	0,04	1,41	1,34	0,91	0,62	0,63	0,62	0,55	0,54	0,63	0,56	4,26	0,62	0,13	0,08	0,05	0,03

For centralized production, T&D refers to transporting hydrogen from the centralized plant till the HRS, including required packing and storage. For distributed production, it includes packing and storage at the HRS. In pathways A and B transmission and distribution is performed by tube-trailers and tank trailers respectively. Pathway C includes transmission by pipeline and distribution via tube trailer while, in pathway D, transmission takes place via tankers ship, from Chile and Norway, and tanker trailers are in charge of distribution.

Table 40. GWP results, in t CO_2eq per truck, for the different truck configurations and for the second set of variables included in the sensitivity analysis.

Component	FCT_{200} kW-700 bar	FCT_{300} kW-700 bar	FCT_{200} kW-350 bar	FCT_{300} kW-350 bar	FCT_{200} kW-LH_2	FCT_{300}kW-LH_2	ICET-700bar	ICET- 350bar
Adblue tank	0,0	0,0	0,0	0,0	0,0	0,0	0,2	0,2
Assembly energy	0,4	0,4	0,4	0,4	0,4	0,4	0,4	0,4
Battery pack	15,6	4,6	15,6	4,6	15,6	4,6	0,0	0,0
Body	4,6	4,6	4,6	4,6	4,6	4,6	4,6	4,6
Exhaust-Aftertreatment	0,0	0,0	0,0	0,0	0,0	0,0	1,9	1,9
Chassis	9,4	9,4	9,4	9,4	9,4	9,4	9,4	9,4
Combustion engine	0,0	0,0	0,0	0,0	0,0	0,0	2,8	2,8
Cooling system	1,3	1,6	1,3	1,6	0,8	1,1	0,5	0,5
Electric motor	1,2	1,2	1,2	1,2	1,2	1,2	0,0	0,0
Fuel cell system	9,4	14,2	9,4	14,2	9,4	14,2	0,0	0,0
H2 tank	46,0	46,0	31,8	31,8	4,4	4,4	55,2	36,3
H2 tank frame	1,9	1,9	1,3	1,3	0,8	0,8	2,3	1,5
Inverter	1,1	1,1	1,1	1,1	1,1	1,1	0,0	0,0
Lead-acid batteries	0,0	0,0	0,0	0,0	0,0	0,0	0,1	0,1
Tank BoP	0,8	0,8	0,8	0,8	0,8	0,8	0,8	0,8
Trailer	38,1	38,1	38,1	38,1	38,1	38,1	38,1	38,1
Transmission	0,4	0,4	0,4	0,4	0,4	0,4	0,9	0,9
Total	130,3	124,4	115,6	109,6	87,1	81,1	117,2	97,6
Second set of variables (sensitivity analysis)								
DRI-steel	117,1	111,2	102,3	96,4	73,9	68,3	102,0	82,4
Recycled CF	93,8	87,8	90,4	84,5	87,1	81,1	73,0	68,6

Table 41. GWP results, in kg CO_2eq, per tkm.

			H_2 production	Truck	T & D	Use	Maintenance	Sum (No leaks)	Leaks GWP-lower	Leaks GWP-upper	Leaks Extra H_2-lower	Leaks Extra H_2-upper
**A**	SgH_2	FCT-700Bar	9,20	5,52	5,24	0	1,29	21,26	1,986	11,326	0,82	2,01
		FCT-350Bar	9,42	5,01	5,20	0	1,33	20,95	2,034	11,597	0,84	2,06
		ICET-700Bar	9,54	4,69	5,44	4,23	0,60	24,50	2,130	11,999	0,82	2,13
		ICET-350Bar	9,40	3,85	5,19	4,17	0,60	23,20	2,099	11,823	0,81	2,10
	GH_2	FCT-700Bar	3,63	5,52	4,99	0	1,29	15,43	1,986	11,326	0,32	0,79
		FCT-350Bar	3,72	5,01	5,11	0	1,33	15,16	2,034	11,597	0,33	0,81
		ICET-700Bar	3,77	4,69	5,17	4,23	0,60	18,46	2,130	11,999	3,76	3,58
		ICET-350Bar	3,71	3,85	5,10	4,17	0,60	17,42	2,099	11,823	3,57	3,51
	BH_2	FCT-700Bar	16,88	5,52	5,24	0	1,29	28,94	1,900	11,326	1,43	3,28
		FCT-350Bar	17,29	5,01	5,20	0	1,33	28,82	1,946	11,597	1,47	3,35
		ICET-700Bar	17,51	4,69	5,44	4,23	0,60	32,47	2,035	11,999	1,45	3,48
		ICET-350Bar	17,25	3,85	5,19	4,17	0,60	31,05	2,005	11,823	1,42	3,43
	BmH_2	FCT-700Bar	-28,11	5,52	5,24	0	1,29	-16,05	1,900	10,060	-2,39	-5,45
		FCT-350Bar	-28,78	5,01	5,20	0	1,33	-17,25	1,946	10,301	-2,45	-5,59
		ICET-700Bar	-29,15	4,69	5,44	4,23	0,60	-14,19	2,035	10,693	-2,41	-5,80
		ICET-350Bar	-28,72	3,85	5,19	4,17	0,60	-14,93	2,005	10,537	-2,37	-5,71
**B**	SgH_2	FCT-LH2	8,78	3,52	3,56	0	1,23	17,08	1,690	15,136	0,70	2,69
	GH_2	FCT-LH2	3,47	3,52	2,28	0	1,23	10,50	1,690	15,136	0,27	1,06
	BH_2	FCT-LH2	16,11	3,52	3,56	0	1,23	24,41	1,607	14,064	1,21	4,58
	BmH_2	FCT-LH2	-26,81	3,52	3,56	0	1,23	-18,51	1,607	14,064	-2,02	-7,63
**C**	SgH_2	FCT-700Bar	9,20	5,52	2,36	0	1,29	18,38	1,657	13,727	0,68	2,44
		FCT-350Bar	9,42	5,01	2,03	0	1,33	17,79	1,696	14,054	0,70	2,49
		ICET-700Bar	9,54	4,69	2,45	4,23	0,60	21,51	1,766	12,233	0,68	2,38
		ICET-350Bar	9,40	3,85	2,03	4,17	0,60	20,04	1,740	12,054	0,67	2,35
	GH_2	FCT-700Bar	3,63	5,52	2,30	0	1,29	12,75	1,657	13,727	0,27	0,96
		FCT-350Bar	3,72	5,01	1,97	0	1,33	12,03	1,696	14,054	0,28	0,99
		ICET-700Bar	3,77	4,69	2,39	4,23	0,60	15,68	1,766	13,432	0,27	0,94
		ICET-350Bar	3,71	3,85	1,97	4,17	0,60	14,29	1,740	13,235	0,27	0,93
	BH_2	FCT-700Bar	16,88	5,52	2,38	0	1,29	26,08	1,569	11,736	1,18	4,08

		FCT-350Bar	17,29	5,01	2,05	0	1,33	25,67	1,607	12,017	1,21	4,18
		ICET-700Bar	17,51	4,69	2,47	4,23	0,60	29,50	1,669	12,172	1,19	3,96
		ICET-350Bar	17,25	3,85	2,04	4,17	0,60	27,91	1,645	11,994	1,17	3,91
	BmH_2	FCT-700Bar	-28,11	5,52	2,38	0	1,29	-18,91	1,569	11,736	-1,97	-6,80
		FCT-350Bar	-28,78	5,01	2,05	0	1,33	-20,39	1,607	12,017	-2,02	-6,96
		ICET-700Bar	-29,15	4,69	2,47	4,23	0,60	-17,16	1,669	12,172	-1,97	-6,60
		ICET-350Bar	-28,72	3,85	2,04	4,17	0,60	-18,07	1,645	11,994	-1,95	-6,50
**D**	GH_2	FCT-LH2	3,47	3,52	15,02	0	1,23	23,24	1,944	17,602	0,32	1,23
	BH_2	FCT-LH2	16,11	3,52	2,58	0	1,23	23,44	1,862	16,606	1,41	5,41
**On-site**	SgH_2	FCT-700Bar	9,46	5,52	0,44	0	1,29	16,72	1,986	10,660	0,69	1,55
		FCT-350Bar	9,69	5,01	0,28	0	1,33	16,30	2,034	10,915	0,70	1,59
		ICET-700Bar	10,43	4,69	0,49	4,23	0,60	20,43	2,130	11,999	0,73	1,50
		ICET-350Bar	10,28	3,85	0,30	4,17	0,60	19,18	2,099	11,823	0,72	1,47
	GH_2	FCT-700Bar	4,06	5,52	0,17	0	1,29	11,04	1,986	11,326	0,30	0,66
		FCT-350Bar	4,16	5,01	0,11	0	1,33	10,60	2,034	11,597	0,30	0,68
		ICET-700Bar	4,21	4,69	0,17	4,23	0,60	13,91	2,130	11,999	0,30	0,60
		ICET-350Bar	4,15	3,85	0,11	4,17	0,60	12,87	2,099	11,823	0,29	0,60

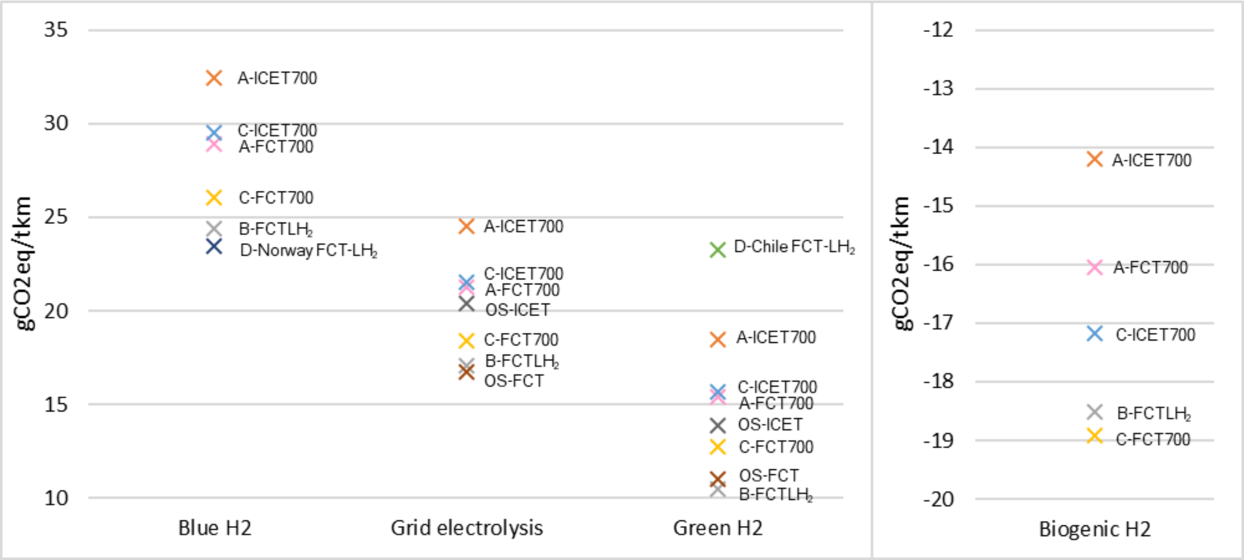

Figure 6. GWP results for FCT and ICET 700 bar. All FCT include a 200 kW fuel cell and 140 kWh LiB.

8.2. Crustal scarcity indicator

Table 42. CSI results, in kg Sieq per kg of produced hydrogen, for the production methods included in this study.

	GH_2		SgH_2		BH_2	BmH_2
	Central	On-site	Central	On-site		Low C seq
Base	3042,5	4252,1	3152,7	4397,8	96,4	433,9
Improved	3022,0	4230,4	3123,0	4362,5	95,5	403,3

Table 43. CSI results, in kg Sieq, for the entire maintenance stage over the entire lifecycle.

	FCT_{300}	FCT_{200}	ICET
Truck tires	3,31E+06	3,31E+06	3,31E+06
Battery pack	4,60E+06	1,51E+07	-
Lead-acid batteries	1,39E+05	1,39E+05	5,82E+05
Total	8,05E+06	1,86E+07	3,89E+06

Table 44. CSI results, in kg Sieq per kg of transported hydrogen. It refers to transport from the production equipment to the refueling nozzle, for the different T&D pathways and on-site production cases.

	A-grid	A-wind	A-grid 350 bar	A-wind 350 bar	B-grid	B-wind	C-grid-GH_2	C-wind-GH_2	C-grid-GH_2-350 bar	C wind GH_2-350 bar	C-grid-BH_2	C-grid-BH_2-350 bar	D-Chile	D-Norway	Onsite-700 bar-grid	Onsite-350 bar-grid	Onsite-700 bar-wind	Onsite-350 bar-wind
	Base case																	
Packing at plant	19,12	14,05	19,12	14,05	92,30	64,16	4,02	2,79	4,02	2,79	0,03	0,03	64,16	46,00	-	-	-	-
Transmission	47,30	47,30	47,30	47,30	17,95	17,95	1,6E-01	1,6E-01	1,6E-01	1,6E-01	1,6E-01	1,62E-01	3,99	8,69	-	-	-	-
Packing at GotHUB	-	-	-	-	-	-	9,56	9,56	9,56	9,56	9,56	9,56	1,57	1,57	-	-	-	-
Purification	-	-	-	-	-	-	7,04	7,04	7,04	7,04	7,04	7,04	-	-	-	-	-	-
Distribution	-	-	-	-	-	-	15,48	15,48	15,48	15,48	15,48	15,48	5,06	5,06	-	-	-	-
Packing at HRS	7,47	7,47	0,00	0,00	4,47	4,47	16,79	16,79	-	-	16,79	-	4,47	4,47	20,85	12,86	14,43	8,91
Total	73,89	68,82	66,42	61,35	114,72	86,58	53,06	51,83	36,27	35,04	49,07	32,28	79,25	65,79	20,85	12,86	14,43	8,91
	Improved case																	
Packing at plant	16,02	11,90	19,12	14,05	78,55	54,71	3,31	2,30	4,02	2,79	0,02	0,03	49,49	35,49	-	-	-	-
Transmission	47,30	47,30	47,30	47,30	17,95	17,95	0,16	0,16	1,6E-01	1,6E-01	0,16	1,62E-01	3,99	8,69	-	-	-	-
Packing at GotHUB	-	-	-	-	-	-	8,02	8,02	9,56	9,56	8,02	9,56	1,57	1,57	-	-	-	-
Purification	-	-	-	-	-	-	7,04	7,04	7,04	7,04	7,04	7,04	-	-	-	-	-	-
Distribution	-	-	-	-	-	-	15,48	15,48	15,48	15,48	15,48	15,48	2,53	2,53	-	-	-	-
Packing at HRS	6,97	6,97	0,00	0,00	4,47	4,47	14,75	14,75	-	-	14,75	-	4,47	4,47	20,85	12,86	12,86	8,91
Total	70,29	66,17	66,42	61,35	100,96	77,13	48,76	47,75	36,27	35,04	45,47	32,28	62,04	52,75	20,85	12,86	12,86	8,91

For centralized production, T&D refers to transporting hydrogen from the centralized plant till the HRS, including required packing and storage. For distributed production, it includes packing and storage at the HRS. In pathways A and B transmission and distribution is performed by tube-trailers and tank trailers respectively. Pathway C includes transmission by pipeline and distribution via tube trailer while, in pathway D, transmission takes place via tankers ship, from Chile and Norway, and tanker trailers are in charge of distribution.

Table 45. CSI results, in kg Sieq per truck, for the different truck configurations.

Component	FCT_{200} kW-700 bar	FCT_{300} kW-700 bar	FCT_{200} kW-350 bar	FCT_{300} kW-350 bar	FCT_{200} kW-LH_2	FCT_{300}kW-LH_2	ICET-700bar	ICET- 350bar
Adblue tank	0,0	0,0	0,0	0,0	0,0	0,0	19,9	19,9
Assembly energy	7,9	7,9	7,9	7,9	7,9	7,9	7,9	7,9
Battery pack	15112,4	4603,3	15112,4	4603,3	15110,6	4602,8	0,0	0,0
Body	1054,1	1054,1	1054,1	1054,1	908,3	908,3	1054,0	1054,0
Exhaust-Aftertreatment	0,0	0,0	0,0	0,0	0,0	0,0	417,9	417,9
Chassis	1592,3	1592,3	1592,3	1592,3	451,0	451,0	1592,1	1592,1
Combustion engine	0,0	0,0	0,0	0,0	0,0	0,0	457,3	457,3
Cooling system	866,4	1039,7	866,4	1039,7	514,9	686,5	346,6	346,6
Electric motor	356,5	356,5	356,5	356,5	355,3	355,3	0,0	0,0
Fuel cell system	59507,8	89261,8	59507,8	89261,8	59507,8	89261,7	0,0	0,0
H2 tank	5041,4	5041,4	3519,6	3519,6	5275,2	5275,2	6020,9	4001,5
H2 tank frame	200,7	200,7	140,5	140,5	86,0	86,0	240,8	162,0
Inverter	1778,2	1778,2	1778,2	1778,2	1778,1	1778,1	0,0	0,0
Lead-acid batteries	138,7	138,7	138,7	138,7	138,7	138,7	581,7	581,7
Tank BoP	1021,7	1021,7	1021,7	1021,7	1021,6	1021,6	1021,6	1021,6
Trailer	4555,1	4555,1	4555,1	4555,1	3673,1	3673,1	4554,7	4554,7
Transmission	70,8	70,8	70,8	70,8	17,2	17,2	162,9	162,9
Total	91304,0	110722,1	89722,0	109140,1	88845,7	108263,4	16478,2	14379,9

Table 46. Total CSI results, in kg Sieq per tkm.

			H_2 production	Truck	T & D	Use	Maintenance	Sum (No leaks)	Leaks Extra H_2-lower	Leaks Extra H_2-upper
**A**	SgH_2	FCT-700Bar	11,35	3,87	0,27	0	0,79	16,27	1,008	2,480
		FCT-350Bar	11,62	3,89	0,24	0	0,80	16,56	1,032	2,539
		ICET-700Bar	11,77	0,66	0,28	2,92E-11	0,16	12,86	1,017	2,627
		ICET-350Bar	11,60	0,57	0,24	2,88E-11	0,15	12,56	1,003	2,589
	GH_2	FCT-700Bar	10,95	3,87	0,25	0	0,79	15,85	0,973	2,393
		FCT-350Bar	11,21	3,89	0,25	0	0,80	16,16	0,996	2,450
		ICET-700Bar	11,36	0,66	0,26	2,92E-11	0,16	12,43	0,982	2,535
		ICET-350Bar	11,19	0,57	0,25	2,88E-11	0,15	12,17	0,968	2,498
	BH_2	FCT-700Bar	0,35	3,87	0,27	0	0,79	5,27	0,030	0,067
		FCT-350Bar	0,36	3,89	0,24	0	0,80	5,30	0,030	0,069
		ICET-700Bar	0,36	0,66	0,28	2,92E-11	0,16	1,45	0,030	0,072
		ICET-350Bar	0,35	0,57	0,24	2,88E-11	0,15	1,32	0,029	0,071
	BmH_2	FCT-700Bar	1,56	3,87	0,27	0	0,79	6,48	0,133	0,303
		FCT-350Bar	1,60	3,89	0,24	0	0,80	6,54	0,136	0,310
		ICET-700Bar	1,62	0,66	0,28	2,92E-11	0,16	2,71	0,134	0,322
		ICET-350Bar	1,60	0,57	0,24	2,88E-11	0,15	2,56	0,132	0,318
**B**	SgH_2	FCT-LH2	10,83	3,59	0,39	0	0,75	15,56	0,857	3,314
	GH_2	FCT-LH2	10,45	3,59	0,30	0	0,75	15,08	0,828	3,198
	BH_2	FCT-LH2	0,33	3,59	0,39	0	0,75	5,06	0,025	0,094
	BmH_2	FCT-LH2	1,49	3,59	0,39	0	0,75	6,22	0,112	0,424
**C**	SgH_2	FCT-700Bar	11,35	3,87	0,19	0	0,79	16,19	0,841	3,005
		FCT-350Bar	11,62	3,89	0,13	0	0,80	16,45	0,861	3,077
		ICET-700Bar	11,77	0,66	0,20	2,92E-11	0,16	12,78	0,844	2,941
		ICET-350Bar	11,60	0,57	0,13	2,88E-11	0,15	12,45	0,831	2,898
	GH_2	FCT-700Bar	10,95	3,87	0,19	0	0,79	15,79	0,812	2,900
		FCT-350Bar	11,21	3,89	0,13	0	0,80	16,04	0,831	2,970
		ICET-700Bar	11,36	0,66	0,19	2,92E-11	0,16	12,37	0,814	2,838
		ICET-350Bar	11,19	0,57	0,13	2,88E-11	0,15	12,04	0,802	2,797
	BH_2	FCT-700Bar	0,35	3,87	0,18	0	0,79	5,18	0,024	0,084

		FCT-350Bar	0,36	3,89	0,12	0	0,80	5,17	0,025	0,086
		ICET-700Bar	0,36	0,66	0,18	2,92E-11	0,16	1,36	0,024	0,082
		ICET-350Bar	0,35	0,57	0,12	2,88E-11	0,15	1,19	0,024	0,080
	BmH_2	FCT-700Bar	1,56	3,87	0,18	0	0,79	6,39	0,110	0,378
		FCT-350Bar	1,60	3,89	0,12	0	0,80	6,41	0,112	0,387
		ICET-700Bar	1,62	0,66	0,18	2,92E-11	0,16	2,62	0,110	0,367
		ICET-350Bar	1,60	0,57	0,12	2,88E-11	0,15	2,44	0,108	0,361
**D**	GH_2	FCT-LH2	10,45	3,59	0,27	0	0,75	15,06	0,952	3,719
	BH_2	FCT-LH2	0,33	3,59	0,23	0	0,75	4,90	0,029	0,111
**On-site**	SgH_2	FCT-700Bar	14,90	3,87	0,07	0	0,79	19,62	1,084	2,438
		FCT-350Bar	15,26	3,89	0,04	0	0,80	20,00	1,110	2,496
		ICET-700Bar	16,42	0,66	0,08	2,92E-11	0,16	17,31	1,154	2,356
		ICET-350Bar	16,18	0,57	0,05	2,88E-11	0,15	16,95	1,137	2,321
	GH_2	FCT-700Bar	15,31	3,87	0,05	0	0,79	20,01	1,113	2,504
		FCT-350Bar	15,67	3,89	0,03	0	0,80	20,40	1,140	2,564
		ICET-700Bar	15,87	0,66	0,05	2,92E-11	0,16	16,74	1,116	2,278
		ICET-350Bar	15,64	0,57	0,03	2,88E-11	0,15	16,40	1,100	2,244

8.3. Particulate matter-Environmental footprint

Table 47. PM-EF results, in incidences per kg PM2.5 emitted, per kg of produced hydrogen, for the production methods included in this study.

	GH_2		SgH_2		BH_2	BmH_2
	Central	On-site	Central	On-site		Low C seq
Base	8,01E-08	1,05E-07	1,78E-07	1,94E-07	1,56E-08	1,05E-07
Improved	7,48E-08	9,74E-08	1,65E-07	1,79E-07	1,51E-08	9,61E-08

Table 48. PM-EF results, in kg Sieq per kg, for the entire maintenance stage over the entire lifecycle.

	FCT300	FCT200	ICET
Truck tires	1,11E-03	1,11E-03	1,11E-03
Battery pack	3,94E-04	1,33E-03	-
Lead-acid batteries	3,89E-06	3,89E-06	1,63E-05
Total	1,51E-03	2,45E-03	1,13E-03

Table 49. PM-EF results, in incidences per kg PM2.5 emitted per kg of transported hydrogen. It refers to transport from the production equipment to the refueling nozzle, for the different T&D pathways and on-site production cases.

	A-grid	A-wind	A-grid 350 bar	A-wind 350 bar	B-grid	B-wind	C-grid-GH_2	C-wind-GH_2	C-grid-GH_2-350 bar	C-wind-GH_2-350 bar	C-grid-BH_2	C-grid-BH_2-350 bar	D-Chile	D-Norway	Onsite-700 bar-grid	Onsite-350 bar-grid	Onsite-700 bar-wind	Onsite-350 bar-wind
	Base case																	
Packing at plant	8,35E-09	3,94E-09	8,35E-09	3,94E-09	4,01E-08	1,67E-08	1,75E-09	7,25E-10	1,75E-09	7,25E-10	2,05E-09	2,05E-09	1,67E-08	1,34E-08	-	-	-	-
Transmission	7,45E-09	7,45E-09	7,45E-09	7,45E-09	3,07E-09	3,07E-09	6,69E-11	6,69E-11	6,69E-11	6,69E-11	6,69E-11	6,69E-11	1,54E-07	1,09E-08	-	-	-	-
Packing at GotHUB	-	-	-	-	-	-	4,18E-09	4,18E-09	4,18E-09	4,18E-09	4,18E-09	4,18E-09	7,15E-10	7,15E-10	-	-	-	-
Purification	-	-	-	-	-	-	3,06E-09	3,06E-09	3,06E-09	3,06E-09	3,06E-09	3,06E-09	-	-	-	-	-	-
Distribution	-	-	-	-	-	-	2,44E-09	2,44E-09	2,44E-09	2,44E-09	2,44E-09	2,44E-09	7,80E-10	7,80E-10	-	-	-	-
Packing at HRS	3,25E-09	3,25E-09	0,00E+00	0,00E+00	1,94E-09	1,94E-09	7,29E-09	7,29E-09	-	-	7,29E-09	-	1,94E-09	1,94E-09	9,04E-09	5,58E-09	3,75E-09	2,31E-09
Total	1,90E-08	1,46E-08	1,58E-08	1,14E-08	4,52E-08	2,17E-08	1,88E-08	1,78E-08	1,15E-08	1,05E-08	1,91E-08	1,18E-08	1,74E-07	2,77E-08	9,04E-09	5,58E-09	3,75E-09	2,31E-09
	Improved case																	
Packing at plant	7,01E-09	3,38E-09	8,35E-09	3,94E-09	3,42E-08	1,43E-08	1,44E-09	5,97E-10	1,75E-09	7,25E-10	1,68E-09	2,05E-09	1,29E-08	1,03E-08	-	-	-	-
Transmission	7,45E-09	7,45E-09	7,45E-09	7,45E-09	3,07E-09	3,07E-09	6,69E-11	6,69E-11	6,69E-11	6,69E-11	6,69E-11	6,69E-11	1,54E-07	1,09E-08	-	-	-	-
Packing at GotHUB	-	-	-	-	-	-	3,50E-09	3,50E-09	4,18E-09	4,18E-09	3,50E-09	4,18E-09	7,15E-10	7,15E-10	-	-	-	-
Purification	-	-	-	-	-	-	3,06E-09	3,06E-09	3,06E-09	3,06E-09	3,06E-09	3,06E-09	-	-	-	-	-	-
Distribution	-	-	-	-	-	-	2,44E-09	2,44E-09	2,44E-09	2,44E-09	2,44E-09	2,44E-09	3,90E-10	3,90E-10	-	-	-	-
Packing at HRS	3,03E-09	3,03E-09	0,00E+00	0,00E+00	1,94E-09	1,94E-09	6,40E-09	6,40E-09	-	-	6,40E-09	-	1,94E-09	1,94E-09	9,04E-09	5,58E-09	3,75E-09	2,31E-09
Total	1,75E-08	1,39E-08	1,58E-08	1,14E-08	3,92E-08	1,94E-08	1,69E-08	1,61E-08	1,15E-08	1,05E-08	1,72E-08	1,18E-08	1,70E-07	2,43E-08	9,04E-09	5,58E-09	3,75E-09	2,31E-09

For the centralized production, it refers to transporting hydrogen from the centralized plants till the HRS, and the subsequent packing and storage. For distributed production, it includes packing and storage at the HRS. In pathways A and B transmission and distribution is performed by tube-trailers and tank trailers respectively. Pathway C includes transmission by pipeline and distribution via tube trailer while, in pathway D, transmission takes place via tankers ship, from Chile and Norway, and tanker trailers are in charge of distribution.

Table 50. PM-EF results, in incidences per kg PM2.5 emitted, for the different truck configurations

Component	FCT200kW-700 bar	FCT300kW-700 bar	FCT200kW-350 bar	FCT300kW-350 bar	FCT200kW-LH_2	FCT300kW-LH_2	ICET-700bar	ICET- 350bar
Adblue tank	0,00E+00	0,00E+00	0,00E+00	0,00E+00	0,00E+00	0,00E+00	1,75E-08	1,75E-08
Assembly energy	4,03E-09	4,03E-09	4,03E-09	4,03E-09	4,03E-09	4,03E-09	4,03E-09	4,03E-09
Battery pack	1,33E-06	3,94E-07	1,33E-06	3,94E-07	1,33E-06	3,94E-07	0,00E+00	0,00E+00
Body	3,51E-07	3,51E-07	3,51E-07	3,51E-07	3,51E-07	3,51E-07	3,51E-07	3,51E-07
Exhaust-Aftertreatment	0,00E+00	0,00E+00	0,00E+00	0,00E+00	0,00E+00	0,00E+00	1,03E-07	1,03E-07
Chassis	8,10E-07	8,10E-07	8,10E-07	8,10E-07	8,10E-07	8,10E-07	8,10E-07	8,10E-07
Combustion engine	0,00E+00	0,00E+00	0,00E+00	0,00E+00	0,00E+00	0,00E+00	2,95E-07	2,95E-07
Cooling system	9,76E-08	1,17E-07	9,76E-08	1,17E-07	5,86E-08	7,81E-08	3,90E-08	3,90E-08
Electric motor	1,06E-07	1,06E-07	1,06E-07	1,06E-07	1,05E-07	1,05E-07	0,00E+00	0,00E+00
Fuel cell system	1,64E-06	2,46E-06	1,64E-06	2,46E-06	1,64E-06	2,46E-06	0,00E+00	0,00E+00
H2 tank	1,01E-06	1,01E-06	7,03E-07	7,03E-07	3,85E-07	3,85E-07	1,21E-06	8,03E-07
H2 tank frame	1,37E-07	1,37E-07	9,61E-08	9,61E-08	5,89E-08	5,89E-08	1,65E-07	1,11E-07
Inverter	7,28E-08	7,28E-08	7,28E-08	7,28E-08	7,28E-08	7,28E-08	0,00E+00	0,00E+00
Lead-acid batteries	3,89E-09	3,89E-09	3,89E-09	3,89E-09	3,89E-09	3,89E-09	1,63E-08	1,63E-08
Tank BoP	7,68E-08	7,68E-08	7,68E-08	7,68E-08	7,68E-08	7,68E-08	7,68E-08	7,68E-08
Trailer	3,34E-06	3,34E-06	3,34E-06	3,34E-06	3,34E-06	3,34E-06	3,34E-06	3,34E-06
Transmission	3,20E-08	3,20E-08	3,20E-08	3,20E-08	3,20E-08	3,20E-08	7,37E-08	7,37E-08
Total	9,01E-06	8,91E-06	8,66E-06	8,57E-06	8,27E-06	8,17E-06	6,50E-06	6,04E-06

Table 51. Total EF-PM results, in incidences per kg PM2.5 emitted per tkm.

			H_2 production	Truck	T & D	Use	Maintenance	Sum (No leaks)	Leaks Extra H_2-lower	Leaks Extra H_2-upper
**A**	SgH_2	FCT-700Bar	6,41E-10	3,82E-10	6,86E-11	0,00E+00	1,04E-10	1,19E-09	5,69E-11	1,40E-10
		FCT-350Bar	6,56E-10	3,76E-10	5,82E-11	0,00E+00	1,06E-10	1,20E-09	5,83E-11	1,43E-10
		ICET-700Bar	6,64E-10	2,60E-10	7,11E-11	3,46E-11	4,50E-11	1,08E-09	5,74E-11	1,48E-10
		ICET-350Bar	6,55E-10	2,38E-10	5,81E-11	3,41E-11	4,44E-11	1,03E-09	5,66E-11	1,46E-10
	GH_2	FCT-700Bar	2,88E-10	3,82E-10	5,27E-11	0,00E+00	1,04E-10	8,26E-10	2,56E-11	6,30E-11
		FCT-350Bar	2,95E-10	3,76E-10	5,39E-11	0,00E+00	1,06E-10	8,31E-10	2,62E-11	6,45E-11
		ICET-700Bar	2,99E-10	2,60E-10	5,46E-11	3,46E-11	4,50E-11	6,94E-10	2,59E-11	6,68E-11
		ICET-350Bar	2,95E-10	2,38E-10	5,38E-11	3,41E-11	4,44E-11	6,65E-10	2,55E-11	6,58E-11
	BH_2	FCT-700Bar	5,63E-11	3,82E-10	6,86E-11	0,00E+00	1,04E-10	6,10E-10	4,78E-12	1,09E-11
		FCT-350Bar	5,76E-11	3,76E-10	5,82E-11	0,00E+00	1,06E-10	5,98E-10	4,90E-12	1,12E-11
		ICET-700Bar	5,84E-11	2,60E-10	7,11E-11	3,46E-11	4,50E-11	4,69E-10	4,82E-12	1,16E-11
		ICET-350Bar	5,75E-11	2,38E-10	5,81E-11	3,41E-11	4,44E-11	4,32E-10	4,75E-12	1,14E-11
	BmH_2	FCT-700Bar	3,76E-10	3,82E-10	6,86E-11	0,00E+00	1,04E-10	9,30E-10	3,20E-11	7,31E-11
		FCT-350Bar	3,85E-10	3,76E-10	5,82E-11	0,00E+00	1,06E-10	9,25E-10	3,28E-11	7,48E-11
		ICET-700Bar	3,90E-10	2,60E-10	7,11E-11	3,46E-11	4,50E-11	8,01E-10	3,22E-11	7,77E-11
		ICET-350Bar	3,85E-10	2,38E-10	5,81E-11	3,41E-11	4,44E-11	7,59E-10	3,18E-11	7,65E-11
**B**	SgH_2	FCT-LH2	6,11E-10	3,34E-10	1,55E-10	0,00E+00	9,88E-11	1,20E-09	4,84E-11	1,87E-10
	GH_2	FCT-LH2	2,75E-10	3,34E-10	7,46E-11	0,00E+00	9,88E-11	7,83E-10	2,18E-11	8,42E-11
	BH_2	FCT-LH2	5,37E-11	3,34E-10	1,55E-10	0,00E+00	9,88E-11	6,42E-10	4,04E-12	1,53E-11
	BmH_2	FCT-LH2	3,59E-10	3,34E-10	1,55E-10	0,00E+00	9,88E-11	9,47E-10	2,70E-11	1,02E-10
**C**	SgH_2	FCT-700Bar	6,41E-10	3,82E-10	6,76E-11	0,00E+00	1,04E-10	1,19E-09	4,75E-11	1,70E-10
		FCT-350Bar	6,56E-10	3,76E-10	4,24E-11	0,00E+00	1,06E-10	1,18E-09	4,86E-11	1,74E-10
		ICET-700Bar	6,64E-10	2,60E-10	7,01E-11	3,46E-11	4,50E-11	1,07E-09	4,76E-11	1,66E-10
		ICET-350Bar	6,55E-10	2,38E-10	4,23E-11	3,41E-11	4,44E-11	1,01E-09	4,69E-11	1,64E-10
	GH_2	FCT-700Bar	2,88E-10	3,82E-10	6,39E-11	0,00E+00	1,04E-10	8,38E-10	2,14E-11	7,64E-11
		FCT-350Bar	2,95E-10	3,76E-10	3,86E-11	0,00E+00	1,06E-10	8,16E-10	2,19E-11	7,82E-11
		ICET-700Bar	2,99E-10	2,60E-10	6,63E-11	3,46E-11	4,50E-11	7,05E-10	2,14E-11	7,47E-11
		ICET-350Bar	2,95E-10	2,38E-10	3,85E-11	3,41E-11	4,44E-11	6,50E-10	2,11E-11	7,36E-11
	BH_2	FCT-700Bar	5,63E-11	3,82E-10	6,87E-11	0,00E+00	1,04E-10	6,10E-10	3,95E-12	1,36E-11

		FCT-350Bar	5,76E-11	3,76E-10	4,35E-11	0,00E+00	1,06E-10	5,83E-10	4,05E-12	1,39E-11
		ICET-700Bar	5,84E-11	2,60E-10	7,13E-11	3,46E-11	4,50E-11	4,69E-10	3,96E-12	1,32E-11
		ICET-350Bar	5,75E-11	2,38E-10	4,34E-11	3,41E-11	4,44E-11	4,18E-10	3,90E-12	1,30E-11
	BmH_2	FCT-700Bar	3,76E-10	3,82E-10	6,87E-11	0,00E+00	1,04E-10	9,30E-10	2,64E-11	9,10E-11
		FCT-350Bar	3,85E-10	3,76E-10	4,35E-11	0,00E+00	1,06E-10	9,11E-10	2,71E-11	9,32E-11
		ICET-700Bar	3,90E-10	2,60E-10	7,13E-11	3,46E-11	4,50E-11	8,01E-10	2,65E-11	8,84E-11
		ICET-350Bar	3,85E-10	2,38E-10	4,34E-11	3,41E-11	4,44E-11	7,45E-10	2,61E-11	8,71E-11
**D**	GH_2	FCT-LH2	2,75E-10	3,34E-10	5,99E-10	0,00E+00	9,88E-11	1,31E-09	2,51E-11	9,79E-11
	BH_2	FCT-LH2	5,37E-11	3,34E-10	9,51E-11	0,00E+00	9,88E-11	5,82E-10	4,69E-12	1,80E-11
**On-site**	SgH_2	FCT-700Bar	6,59E-10	3,82E-10	3,06E-11	0,00E+00	1,04E-10	1,17E-09	4,79E-11	1,08E-10
		FCT-350Bar	6,75E-10	3,76E-10	1,93E-11	0,00E+00	1,06E-10	1,18E-09	4,91E-11	1,10E-10
		ICET-700Bar	7,26E-10	2,60E-10	3,37E-11	3,46E-11	4,50E-11	1,10E-09	5,10E-11	1,04E-10
		ICET-350Bar	7,15E-10	2,38E-10	2,05E-11	3,41E-11	4,44E-11	1,05E-09	5,03E-11	1,03E-10
	GH_2	FCT-700Bar	3,78E-10	3,82E-10	1,35E-11	0,00E+00	1,04E-10	8,77E-10	2,75E-11	6,19E-11
		FCT-350Bar	3,87E-10	3,76E-10	8,53E-12	0,00E+00	1,06E-10	8,78E-10	2,82E-11	6,34E-11
		ICET-700Bar	3,92E-10	2,60E-10	1,40E-11	3,46E-11	4,50E-11	7,46E-10	2,76E-11	5,63E-11
		ICET-350Bar	3,87E-10	2,38E-10	8,51E-12	3,41E-11	4,44E-11	7,12E-10	2,72E-11	5,55E-11

References

1. Miotti, M., Hofer, J., and Bauer, C. (2017). Integrated environmental and economic assessment of current and future fuel cell vehicles. Int. J. Life Cycle Assess. *22*, 94–110. https://doi.org/10.1007/s11367-015-0986-4.

2. Velandia Vargas, J.E., and Seabra, J.E.A. (2021). Fuel-cell technologies for private vehicles in Brazil: Environmental mirage or prospective romance? A comparative life cycle assessment of PEMFC and SOFC light-duty vehicles. Sci. Total Environ. *798*, 149265. https://doi.org/10.1016/j.scitotenv.2021.149265.

3. Evangelisti, S., Tagliaferri, C., Brett, D.J.L., and Lettieri, P. (2017). Life cycle assessment of a polymer electrolyte membrane fuel cell system for passenger vehicles. J. Clean. Prod. *142*, 4339–4355. https://doi.org/10.1016/j.jclepro.2016.11.159.

4. Simons, A., and Bauer, C. (2015). A life-cycle perspective on automotive fuel cells. Appl. Energy *157*, 884–896. https://doi.org/10.1016/j.apenergy.2015.02.049.

5. Bekel, K., and Pauliuk, S. (2019). Prospective cost and environmental impact assessment of battery and fuel cell electric vehicles in Germany. Int. J. Life Cycle Assess. *24*, 2220–2237. https://doi.org/10.1007/s11367-019-01640-8.

6. Benitez, A., Wulf, C., Palmenaer, A. De, Lengersdorf, M., and Kuckshinrichs, W. (2021). Ecological assessment of fuel cell electric vehicles with special focus on type IV carbon fiber hydrogen tank. J. Clean. Prod. *278*. https://doi.org/https://doi.org/10.1016/j.jclepro.2020.123277.

7. Valente, A., Iribarren, D., and Dufour, J. (2018). Harmonising the cumulative energy demand of renewable hydrogen for robust comparative life-cycle studies. J. Clean. Prod. *175*, 384–393. https://doi.org/10.1016/j.jclepro.2017.12.069.

8. Joint Research Centre (2020). JEC Tank-To-Wheels report v5 : Heavy duty vehicles. https://doi.org/10.2760/541016.

9. Valente, A., Iribarren, D., and Dufour, J. (2017). Life cycle assessment of hydrogen energy systems: a review of methodological choices. Int. J. Life Cycle Assess. *22*, 346–363. https://doi.org/10.1007/s11367-016-1156-z.

10. Ricardo Energy & Environment (2020). Determining the environmental impacts of conventional and alternatively fuelled vehicles through LCA. Eur. Comm., 456. https://op.europa.eu/sv/publication-detail/-/publication/1f494180-bc0e-11ea-811c-01aa75ed71a1.

11. Drawer, C., Rödl, A., and Kaltschmitt, M. (2024). Life cycle assessment of construction and driving operation of a hydrogen-powered truck built from a used diesel truck. Transp. Res. Interdiscip. Perspect. *24*. https://doi.org/10.1016/j.trip.2024.101020.

12. Booto, G.K., Aamodt Espegren, K., and Hancke, R. (2021). Comparative life cycle assessment of heavy-duty drivetrains: A Norwegian study case. Transp. Res. Part D Transp. Environ. *95*, 102836. https://doi.org/10.1016/j.trd.2021.102836.

13. ICCT (2023). A comparison of the life-cycle greenhouse gas emissions of European heavy-duty vehicles and fuels. 36. https://doi.org/https://theicct.org/publication/lca-ghg-emissions-hdv-fuels-europe-feb23/.

14. Lee, D.-Y., Elgowainy, A., Kotz, A., Vijayagopal, R., and Marcinkoski, J. (2018). Life-cycle implications of hydrogen fuel cell electric vehicle technology for medium- and heavy-duty trucks. J. Power Sources *393*, 217–229. https://doi.org/10.1016/j.jpowsour.2018.05.012.

15. Liu, X., Reddi, K., Elgowainy, A., Lohse-Busch, H., Wang, M., and Rustagi, N. (2020). Comparison of well-to-wheels energy use and emissions of a hydrogen fuel cell electric vehicle relative to a conventional gasoline-powered internal combustion engine vehicle. Int. J. Hydrogen Energy *45*, 972–983. https://doi.org/10.1016/j.ijhydene.2019.10.192.

16. Joint Research Centre (2020). JEC Well-To-Wheels report v5. Rep. JCR, EUCAR Concawe. https://doi.org/10.2760/100379.

17. Sacchi, R., Bauer, C., and Cox, B.L. (2021). Does Size Matter? The Influence of Size, Load Factor,

Range Autonomy, and Application Type on the Life Cycle Assessment of Current and Future Medium- and Heavy-Duty Vehicles. Environ. Sci. Technol. *55*, 5224–5235. https://doi.org/10.1021/acs.est.0c07773.

18. Frank, E.D., Elgowainy, A., Reddi, K., and Bafana, A. (2021). Life-cycle analysis of greenhouse gas emissions from hydrogen delivery: A cost-guided analysis. Int. J. Hydrogen Energy *46*, 22670–22683. https://doi.org/10.1016/j.ijhydene.2021.04.078.

19. European Commission (2024). Environmental life cycle assessment (LCA) comparison of hydrogen delivery options within Europe. https://doi.org/10.2760/5459.

20. Tayarani, H., and Ramji, A. (2022). Life Cycle Assessment of Hydrogen Transportation Pathways via Pipelines and Truck Trailers: Implications as a Low Carbon Fuel. Sustainability *14*, 12510. https://doi.org/10.3390/su141912510.

21. Lotrič, A., Sekavčnik, M., Kuštrin, I., and Mori, M. (2021). Life-cycle assessment of hydrogen technologies with the focus on EU critical raw materials and end-of-life strategies. Int. J. Hydrogen Energy *46*, 10143–10160. https://doi.org/10.1016/j.ijhydene.2020.06.190.

22. Weiszflog, E., and Abbas, M. (2022). Life Cycle Assessment of Hydrogen Storage Systems for Trucks.

23. Franz, S., and Liljenroth, A. (2020). Life cycle assessment of a fuel cell electric vehicle with an MS-100 system A comparison between a fuel cell electric vehicle and a battery electric vehicle. Dep. Technol. Manag. Econ. Div. Environ. Syst. Anal. Chalmers Univ. Technol., 8–9.

24. Powercell (2021). PowerCellution Power Generation System 100. Powercell Gr. https://powercellgroup.com/product/power-generation-system-100/.

25. Wolff, S., Seidenfus, M., Gordon, K., Álvarez, S., Kalt, S., and Lienkamp, M. (2020). Scalable Life-Cycle Inventory for Heavy-Duty Vehicle Production. Sustainability *12*, 5396. https://doi.org/10.3390/su12135396.

26. Iyer, R., Kelly, J., and Elgowainy, A. (2021). Vehicle-cycle Inventory for Medium and Heavy-duty vehicles. https://publications.anl.gov/anlpubs/2021/10/171707.pdf.

27. Argonne National Laboratory (2023). GREET 2022 at Argonne National Laboratory.

28. Usai, L., Hung, C.R., Vásquez, F., Windsheimer, M., Burheim, O.S., and Strømman, A.H. (2021). Life cycle assessment of fuel cell systems for light duty vehicles, current state-of-the-art and future impacts. J. Clean. Prod. *280*, 125086. https://doi.org/10.1016/j.jclepro.2020.125086.

29. Simons, S., and Azimov, U. (2021). Comparative Life Cycle Assessment of Propulsion Systems for Heavy-Duty Transport Applications. Energies *14*, 3079. https://doi.org/10.3390/en14113079.

30. European Commission (2020). Life cycle assessment of hydrogen and fuel cell technologies: Inventory of work performed by projects funded under FCH JU. https://doi.org/10.2760/434747.

31. ITF (2021). Permissible Maximum Dimensions of Lorries in Europe- Sweden. https://www.itf-oecd.org/road-transport-group/weights-and-dimensions/sweden.

32. VBG (2022). What is the Maximum Permitted Weight and Length for Freight Transport in the Nordics? https://blog.vbg.eu/en/nordic-regulations.

33. European Union (2019). Regulation (EU) 2019/1242 setting CO2 emission performance standards for new heavy-duty vehicles. https://eur-lex.europa.eu/eli/reg/2019/1242/oj.

34. Hydrogen Europe (2023). Hydrogen Europe Position Paper- Weights and Dimensions Directive. 1–24. https://hydrogeneurope.eu/wp-content/uploads/2023/10/Hydrogen-Europe-Weights-and-Dimensions-position-paper.pdf?utm_source=chatgpt.com.

35. Johansson, M., and Hanarp, P. (2023). Personal communication with Volvo experts.

36. Nordelöf, A., Alatalo, M., and Söderman, M.L. (2019). A scalable life cycle inventory of an automotive power electronic inverter unit—part I: design and composition. Int. J. Life Cycle Assess. *24*, 78–92. https://doi.org/10.1007/s11367-018-1503-3.

37. Nordelöf, A., Grunditz, E., Tillman, A.-M., Thiringer, T., and Alatalo, M. (2018). A scalable life cycle inventory of an electrical automotive traction machine—Part I: design and composition. Int. J. Life

Cycle Assess. *23*, 55–69. https://doi.org/10.1007/s11367-017-1308-9.

38. Ellingsen, L., Thorne, R.J., Wind, J., Figenbaum, E., Romare, M., and Nordelöf, A. (2022). Life cycle assessment of battery electric buses. Transp. Res. Part D Transp. Environ. *112*, 103498. https://doi.org/10.1016/j.trd.2022.103498.

39. AVK (2024). The European market for Fiber-Reinforced Plastics/Composites 2023. Ind. Verstärkte Kunststoffe, 42. https://eucia.eu/wp-content/uploads/2024/05/avk_marketreport_2024_final_eng.pdf.

40. RISE (2022). Liquid Hydrogen As A Logistic Fuel – A Pre-study. *1*. https://www.diva-portal.org/smash/get/diva2:1690616/FULLTEXT01.pdf.

41. ACEA (2015). Heavy-Duty Vehicle Weight Restrictions in the EU. https://www.acea.auto/files/SAG_23_Heavy-Duty_Vehicle_Weight_Restrictions_in_the_EU.pdf.

42. Kies, A. (2023). Personal communication with Scania expert.

43. Sheffield, J.W., Martin, K.B., and Folkson, R. (2014). Electricity and hydrogen as energy vectors for transportation vehicles. Altern. Fuels Adv. Veh. Technol. Improv. Environ. Perform., 117–137. https://doi.org/10.1533/9780857097422.1.117.

44. Folkson, R. (2022). Hydrogen as an energy vector for transportation vehicles. Altern. Fuels Adv. Veh. Technol. Improv. Environ. Perform., 151–171. https://doi.org/10.1016/B978-0-323-90979-2.00013-5.

45. Genovese, M., and Fragiacomo, P. (2023). Hydrogen refueling station: Overview of the technological status and research enhancement. J. Energy Storage *61*, 106758. https://doi.org/10.1016/j.est.2023.106758.

46. Rödl, A., Wulf, C., and Kaltschmitt, M. (2018). Assessment of Selected Hydrogen Supply Chains—Factors Determining the Overall GHG Emissions. Hydrog. Supply Chain., 81–109. https://doi.org/10.1016/B978-0-12-811197-0.00003-8.

47. Dagdougui, H., Sacile, R., Bersani, C., and Ouammi, A. (2018). Hydrogen Storage and Distribution: Implementation Scenarios. Hydrog. Infrastruct. Energy Appl., 37–52. https://doi.org/10.1016/B978-0-12-812036-1.00004-4.

48. Ahluwalia, R.K., Roh, H.-S., Peng, J.-K., Papadias, D., Baird, A.R., Hecht, E.S., Ehrhart, B.D., Muna, A., Ronevich, J.A., Houchins, C., et al. (2023). Liquid hydrogen storage system for heavy duty trucks: Configuration, performance, cost, and safety. Int. J. Hydrogen Energy *48*, 13308–13323. https://doi.org/10.1016/j.ijhydene.2022.12.152.

49. Frazer-Nash Consultancy (2022). Fugitive Hydrogen Emissions in a Future Hydrogen Economy. 1–52. https://www.gov.uk/government/publications/fugitive-hydrogen-emissions-in-a-future-hydrogen-economy.

50. Warwick, N., Griffiths, P., Keeble, J., Archibald, A., Pyle, J., and Shine, K. (2022). Atmospheric implications of increased hydrogen use. Dep. Business, Energy Ind. Strateg., 75. https://assets.publishing.service.gov.uk/government/uploads/system/uploads/attachment_data/file/1067144/atmospheric-implications-of-increased-hydrogen-use.pdf#:~:text=%60Atmospheric implications of increased hydrogen use An increase,stratospheric %28%3E40.

51. Sand, M., Skeie, R.B., Sandstad, M., Krishnan, S., Myhre, G., Bryant, H., Derwent, R., Hauglustaine, D., Paulot, F., Prather, M., et al. (2023). A multi-model assessment of the Global Warming Potential of hydrogen. Commun. Earth Environ. *4*, 203. https://doi.org/10.1038/s43247-023-00857-8.

52. Campari, A., Ustolin, F., Alvaro, A., and Paltrinieri, N. (2023). A review on hydrogen embrittlement and risk-based inspection of hydrogen technologies. Int. J. Hydrogen Energy. https://doi.org/10.1016/j.ijhydene.2023.05.293.

53. IEA (2017). The Future of Trucks. Futur. Truck. https://doi.org/10.1787/9789264279452-en.

54. Cullen, D.A., Neyerlin, K.C., Ahluwalia, R.K., Mukundan, R., More, K.L., Borup, R.L., Weber, A.Z., Myers, D.J., and Kusoglu, A. (2021). New roads and challenges for fuel cells in heavy-duty transportation. Nat. Energy *6*, 462–474. https://doi.org/10.1038/s41560-021-00775-z.

55. WPIC (2022). Fuel cell electric vehicles and platinum demand. World Platin. Invest. Counc. https://www.cmegroup.com/articles/2022/wpic-fuel-cell-electric-vehicles-and-platinum-demand.html.

56. Office of Energy Efficiency & Renewable Energy (2016). DOE Technical Targets for Polymer

Electrolyte Membrane Fuel Cell Components. https://www.energy.gov/eere/fuelcells/doe-technical-targets-polymer-electrolyte-membrane-fuel-cell-components.

57. Burton, M., and Biesheuvel, T. (2019). Miners Find Out the Hard Way Why Cobalt Is Called the Goblin. Bloomberg. https://www.bloomberg.com/news/articles/2019-08-07/miners-find-out-the-hard-way-why-cobalt-is-called-the-goblin.

58. Berckmans, G., Messagie, M., Smekens, J., Omar, N., Vanhaverbeke, L., and Mierlo, J. Van (2017). Cost projection of state of the art lithium-ion batteries for electric vehicles up to 2030. Energies *10*. https://doi.org/10.3390/en10091314.

59. Blomgren, G.E. (2017). The Development and Future of Lithium Ion Batteries. J. Electrochem. Soc. *164*, A5019–A5025. https://doi.org/10.1149/2.0251701jes.

60. Frith, J.T., Lacey, M.J., and Ulissi, U. (2023). A non-academic perspective on the future of lithium-based batteries. Nat. Commun. *14*, 420. https://doi.org/10.1038/s41467-023-35933-2.

61. Farchy, J., and Warren, H. (2018). China Has a Secret Weapon in the Race to Dominate Electric Cars. Bloomberg. https://www.bloomberg.com/graphics/2018-china-cobalt/.

62. The Guardian (2023). Europe is ‘miles behind’ in race for raw materials used in electric car batteries. https://www.theguardian.com/business/2023/dec/04/europe-miles-behind-race-raw-materials-electric-car-batteries-lithium-cobalt-nickel.

63. Chordia, M., Nordelöf, A., and Ellingsen, L.A.-W. (2021). Environmental life cycle implications of upscaling lithium-ion battery production. Int. J. Life Cycle Assess. *26*, 2024–2039. https://doi.org/10.1007/s11367-021-01976-0.

64. Volvotrucks (2022). Volvo Trucks opens battery plant in Belgium. https://www.volvotrucks.com/en-en/news-stories/press-releases/2022/may/volvo-trucks-opens-battery-plant-in-belgium.html.

65. Cummins (2024). How do aftertreatment systems differ between hydrogen and diesel engines? https://www.cummins.com/news/2024/08/29/how-do-aftertreatment-systems-differ-between-hydrogen-and-diesel-engines.

66. Zambelli, C. (2023). Degree Project in Sustainable Energy Engineering Second cycle 30 credits Exhaust system for hydrogen fuelled combustion engines: effect of high-water content on the SCR vanadium catalyst.

67. Özyalcin, C., Sterlepper, S., Roiser, S., Eichlseder, H., and Pischinger, S. (2024). Exhaust gas aftertreatment to minimize NOX emissions from hydrogen-fueled internal combustion engines. Appl. Energy *353*, 122045. https://doi.org/10.1016/j.apenergy.2023.122045.

68. IRENA (2020). Green Hydrogen Cost Reduction. Scaling up Electrolysers to Meet the 1.5^{0}C Climate Goal. 105. https://irena.org/-/media/Files/IRENA/Agency/Publication/2020/Dec/IRENA_Green_hydrogen_cost_2020.pdf.

69. IEA (2019). The Future of Hydrogen for G20. Seizing today’s opportunities. Int. Energy Agency *6*, 246–256.

70. IEA (2022). Global Hydrogen Review 2022. https://iea.blob.core.windows.net/assets/c5bc75b1-9e4d-460d-9056-6e8e626a11c4/GlobalHydrogenReview2022.pdf.

71. HyBalance (2020). Hybalance. https://hybalance.eu/.

72. Delpierre, M., Quist, J., Mertens, J., Prieur-Vernat, A., and Cucurachi, S. (2021). Assessing the environmental impacts of wind-based hydrogen production in the Netherlands using ex-ante LCA and scenarios analysis. J. Clean. Prod. *299*, 126866. https://doi.org/10.1016/j.jclepro.2021.126866.

73. Wulf, C., and Kaltschmitt, M. (2018). Hydrogen Supply Chains for Mobility—Environmental and Economic Assessment. Sustainability *10*, 1699. https://doi.org/10.3390/su10061699.

74. U.S. Department of energy (2018). Fuel cell technologies office. https://www.energy.gov/sites/prod/files/2015/11/f27/fcto_fuel_cells_fact_sheet.pdf.

75. Hydrogenics (2019). Electrolyzer datasheet. 1–37. https://etipwind.eu/wp-content/uploads/A2-Hydrogenics_v2.pdf.

76. Department for business energy & industrial strategy (2019). Hy4Heat. Hydrogen Purity.

77. Dawood, F., Anda, M., and Shafiullah, G.M. (2020). Hydrogen production for energy: An overview. Int. J. Hydrogen Energy *45*, 3847–3869. https://doi.org/10.1016/j.ijhydene.2019.12.059.

78. Basile, A., Dalena, F., Tong, J., and Verziroglu, T. (2016). Hydrogen production, separation and purification for energy. https://medium.com/@arifwicaksanaa/pengertian-use-case-a7e576e1b6bf.

79. IEA (2023). Global Hydrogen Review 2023. Glob. Hydrog. Rev. 2023. https://doi.org/10.1787/cb2635f6-en.

80. Gulotta, T.M., Salomone, R., Mondello, G., Saija, G., Lanuzza, F., and Briguglio, N. (2023). Life Cycle Assessment and Environmental Life Cycle costing of a unitised regenerative fuel cell stack. Sci. Total Environ. *901*, 166007. https://doi.org/10.1016/j.scitotenv.2023.166007.

81. Arvidsson, R., Söderman, M.L., Sandén, B.A., Nordelöf, A., André, H., and Tillman, A.-M. (2020). A crustal scarcity indicator for long-term global elemental resource assessment in LCA. Int. J. Life Cycle Assess. *25*, 1805–1817. https://doi.org/10.1007/s11367-020-01781-1.

82. National Renewable Energy Laboratory (2018). H2A: Hydrogen Analysis Production Case Studies. https://www.nrel.gov/hydrogen/h2a-production-models.html.

83. Papadias, D.D., and Ahluwalia, R.K. (2021). Bulk storage of hydrogen. Int. J. Hydrogen Energy *46*, 34527–34541. https://doi.org/10.1016/j.ijhydene.2021.08.028.

84. NASA (2022). World’s Largest Liquid Hydrogen Tank Nearing Completion. https://ntrs.nasa.gov/api/citations/20220004276/downloads/Cold Facts_LH2 Sphere Update.pdf.

85. Siemens energy (2020). Silyzer 300: The next paradigm of PEM electrolysis. https://assets.siemens-energy.com/siemens/assets/api/uuid:a193b68f-7ab4-4536-abe2-c23e01d0b526/datasheet-silyzer300.pdf.

86. Siemens energy (2021). At the Dawn of the Hydrogen Economy. https://www.powermag.com/siemens-dawn-hydrogen-economy/.

87. International Energy Agency (2019). The Future of Hydrogen. https://www.iea.org/reports/the-future-of-hydrogen.

88. European parliament (2021). EU Hydrogen policy: Hydrogen as an energy carrier for a climate-neutral economy. Eur. Parliam. Res. Serv., 8.

89. IRENA (2022). Global hydrogen trade to meet the 1.5 °C climate goal: Part I Trade outlook for 2050 and way forward. https://www.irena.org/-/media/Files/IRENA/Agency/Publication/2022/Apr/IRENA_Global_Trade_Hydrogen_2022.pdf.

90. IPCC (2023). IPCC, 2023: Climate Change 2023: Synthesis Report. Contribution of Working Groups I, II and III to the Sixth Assessment Report of the Intergovernmental Panel on Climate Change [Core Writing Team, H. Lee and J. Romero (eds.)]. IPCC, Geneva, Switzerland. https://doi.org/10.59327/IPCC/AR6-9789291691647.

91. Congressional Research Service (2022). Carbon Capture and Sequestration (CCS) in the United States. 22. https://fas.org/sgp/crs/misc/R44902.pdf.

92. IEEFA (2022). The carbon capture crux: Lessons learned. https://ieefa.org/resources/carbon-capture-crux-lessons-learned.

93. IEAGHG (2022). Low-Carbon Hydrogen from Natural Gas : Global Roadmap. 144.

94. Energy brief (2022). No green without blue: Europe could fall short of its energy goals without energy imports. https://energy-europe.eu/renewables-en/no-green-without-blue-europe-could-fall-short-of-its-energy-goals-without-hydrogen-imports/.

95. Deloitte Finance, IFPEN;, and SINTEF (2021). Hydrogen4EU. Charting pathways for net zero. https://www.concawe.eu/wp-content/uploads/Hydrogen4EU_Report_Final.pdf.

96. Sørensen, B., and Spazzafumo, G. (2018). Hydrogen. Hydrog. Fuel Cells, 5–105. https://doi.org/10.1016/B978-0-08-100708-2.00002-3.

97. IPCC (2005). Carbon dioxide capture and storage.

98. Sundén, B. (2019). Hydrogen. Hydrog. Batter. Fuel Cells, 37–55. https://doi.org/10.1016/B978-0-12-

816950-6.00003-8.

99. Antonini, C., Treyer, K., Streb, A., van der Spek, M., Bauer, C., and Mazzotti, M. (2020). Hydrogen production from natural gas and biomethane with carbon capture and storage – A techno-environmental analysis. Sustain. Energy Fuels *4*, 2967–2986. https://doi.org/10.1039/D0SE00222D.

100. Howarth, R.W., and Jacobson, M.Z. (2021). How green is blue hydrogen ? Energy Sci. Eng., 1676–1687. https://doi.org/https://doi.org/10.1002/ese3.956.

101. IEAGHG (2019). Reference data and Supporting Literature Reviews for SMR Based Hydrogen Production with CCS. Int. Energy Agency *i*, 294. https://doi.org/10.1016/j.energy.2019.07.072%0Ahttp://dx.doi.org/10.1016/j.ijhydene.2016.01.009%0Awww.ieaghg.org%0Ahttps://www.intechopen.com/books/advanced-biometric-technologies/liveness-detection-in-biometrics.

102. Muradov, N. (2015). Low-carbon production of hydrogen from fossil fuels. Compend. Hydrog. Energy, 489–522. https://doi.org/10.1016/b978-1-78242-361-4.00017-0.

103. Bauer, C., Treyer, K., Antonini, C., Bergerson, J., Gazzani, M., Gencer, E., Gibbins, J., Mazzotti, M., McCoy, S.T., McKenna, R., et al. (2022). On the climate impacts of blue hydrogen production. Sustain. Energy Fuels *6*, 66–75. https://doi.org/10.1039/d1se01508g.

104. IEAGHG (2017). Techno - Economic Evaluation of SMR Based Standalone (Merchant) Hydrogen Plant with CCS. IEA Greenh. gas R&D Program. https://ieaghg.org/exco_docs/2017-02.pdf.

105. Ueckerdt, F., Verpoort, P.C., Anantharaman, R., Bauer, C., Beck, F., Longden, T., and Roussanaly, S. (2024). On the cost competitiveness of blue and green hydrogen. Joule *8*, 104–128. https://doi.org/10.1016/j.joule.2023.12.004.

106. Khojasteh Salkuyeh, Y., Saville, B.A., and MacLean, H.L. (2017). Techno-economic analysis and life cycle assessment of hydrogen production from natural gas using current and emerging technologies. Int. J. Hydrogen Energy *42*, 18894–18909. https://doi.org/10.1016/j.ijhydene.2017.05.219.

107. IEA (2024). Global Methane Tracker. https://iea.blob.core.windows.net/assets/d42fc095-f706-422a-9008-6b9e4e1ee616/GlobalMethaneTracker_Documentation.pdf.

108. Ecoinvent (2022). Ecoinvent v 3.8. https://ecoinvent.org/.

109. Norsk olje and gass (2017). Environmental work by the oil and gas industry. Facts and development trends environmental report. https://www.readkong.com/page/2017-environmental-report-environmental-work-by-the-oil-6335314.

110. Mehmeti, A., Angelis-dimakis, A., Arampatzis, G., McPhail, S., and Ulgiati, S. (2018). Life Cycle Assessment and Water Footprint of Hydrogen Production Methods : From Conventional to Emerging Technologies. Environments *5*, 1–19. https://doi.org/10.3390/environments5020024.

111. Blanco, H. (2022). What's best for Hydrogen transport: ammonia, liquid hydrogen, LOHC or pipelines? - Energy Post. energypost.eu. https://energypost.eu/whats-best-for-hydrogen-transport-ammonia-liquid-hydrogen-lohc-or-pipelines/.

112. Anthonsen, K.L., and Christensen, N.P. (2021). EU Geological CO_2 storage summary. https://www.catf.us/2021/10/europe-geologic-storage-summary/.

113. Financial Times (2022). 'Put up or shut up': can Big Oil prove the case for carbon capture? https://www.ft.com/content/b8d6848d-1e8a-4c57-b65b-52105b48b178.

114. Corporate Europe Observatory (2023). The dirty truth about the EU's hydrogen push. https://corporateeurope.org/en/dirty-truth-about-EU-hydrogen-push#:~:text=Together%2C the top 25 hydrogen,lobby register%27s top 100 spenders.

115. The Guardian (2021). Oil firms made 'false claims' on blue hydrogen costs, says ex-lobby boss. https://www.theguardian.com/environment/2021/aug/20/oil-firms-made-false-claims-on-blue-hydrogen-costs-says-ex-lobby-boss.

116. IEEFA (2023). Blue hydrogen: Not clean, not low carbon, not a solution. Inst. Energy Econ. Financ. Anal. https://ieefa.org/resources/blue-hydrogen-not-clean-not-low-carbon-not-solution#:~:text=The fossil fuel industry promises,neither clean nor low-carbon.

117. Global CCS Institute (2022). Global Status of CCS 2022. https://status22.globalccsinstitute.com/wp-

content/uploads/2022/12/Global-Status-of-CCS-2022_Download_1222.pdf.

118. IEAGHG (2017). The carbon capture project at Air Products' Port Arthur Hydrogen production facility. *01*, 1–7. https://ieaghg-publications.s3.eu-north-1.amazonaws.com/Technical+Reports/2018-05+The+CCS+Project+at+Air+Products'+Port+Arthur+Hydrogen+Production+Facility.pdf.

119. Reuters (2023). Carbon capture project back at Texas coal plant after 3-year shutdown. https://www.reuters.com/business/energy/carbon-capture-project-back-texas-coal-plant-after-3-year-shutdown-2023-09-14/.

120. Reuters (2020). Problems plagued U.S. CO2 capture project before shutdown: document. https://www.reuters.com/article/business/environment/problems-plagued-us-co2-capture-project-before-shutdown-document-idUSKCN2523K7/.

121. ABC News (2021). As carbon capture, storage commitments near $4b, what are the options for heavy industry? https://www.abc.net.au/news/2021-08-21/taxpayer-bill-for-carbon-capture-and-storage-hits-4-billion/100375854.

122. RISC (2023). Asia-Pacific CCS Overview & Gorgon. 'Not a CCS problem.'

123. The Guardian (2022). The cost to capture carbon? More water and electricity | Water. https://www.theguardian.com/environment/2022/oct/15/emissions-capture-carbon-cost-water-electricity.

124. IEA (2021). Methane Tracker. https://www.iea.org/data-and-statistics/data-tools/methane-tracker-data-explorer.

125. Saunois, M., Stavert, A.R., Poulter, B., Bousquet, P., Canadell, J.G., Jackson, R.B., Raymond, P.A., Dlugokencky, E.J., Houweling, S., Patra, P.K., et al. (2020). The Global Methane Budget 2000–2017. Earth Syst. Sci. Data *12*, 1561–1623. https://doi.org/10.5194/essd-12-1561-2020.

126. Sherwin, E.D., El Abbadi, S.H., Burdeau, P.M., Zhang, Z., Chen, Z., Rutherford, J.S., Chen, Y., and Brandt, A.R. (2024). Single-blind test of nine methane-sensing satellite systems from three continents. Atmos. Meas. Tech. *17*, 765–782. https://doi.org/10.5194/amt-17-765-2024.

127. Scarlat, N., Dallemand, J.-F., and Fahl, F. (2018). Biogas: Developments and perspectives in Europe. Renew. Energy *129*, 457–472. https://doi.org/10.1016/j.renene.2018.03.006.

128. Biocycle (2018). Basics of biogas upgrading. https://www.biocycle.net/basics-biogas-upgrading/.

129. World Biogas Association (2019). Global potential of biogas. World Biogas Assoc., 56. https://www.worldbiogasassociation.org/global-potential-of-biogas/.

130. Cherubini, F., and Strømman, A.H. (2011). Life cycle assessment of bioenergy systems: State of the art and future challenges. Bioresour. Technol. *102*, 437–451. https://doi.org/10.1016/j.biortech.2010.08.010.

131. IPCC (2013). AR5 Climate Change 2013: The Physical Science Basis. https://www.ipcc.ch/report/ar5/wg1/.

132. Petersson, A., and Wellinger, A. (2009). Biogas upgrading technologies–developments and innovations. IEA Bioenergy, 20. http://typo3.dena.de/fileadmin/biogas/Downloads/Studien/IEA-BiogasUpgradingTechnologies2009.pdf.

133. Alberto Huerta-Reynoso, E., Alfredo López-Aguilar, H., Alberto Gómez, J., Guadalupe Gómez-Méndez, M., and Pérez-Hernández, A. (2019). Biogas Power Energy Production from a Life Cycle Thinking. New Front. Life Cycle Assess. - Theory Appl. https://doi.org/10.5772/intechopen.82250.

134. Energigas Sverige (2020). Produktion av biogas och rötrester och dess användning år 2020. https://www.energigas.se/media/3zyj1lrf/biogasstatistikrapport_2020-energigas-sverige.pdf.

135. IEA Bioenergy (2017). Methane emissions from biogas plants. IEA Bioenergy - Task 37, 52. https://www.ieabioenergy.com/wp-content/uploads/2018/01/Methane-Emission_web_end_small.pdf.

136. European Commission (2021). Assessment of Hydrogen Delivery Options. Eur. Com., 4.

137. Reuß, M., Dimos, P., Léon, A., Grube, T., Robinius, M., and Stolten, D. (2021). Hydrogen Road Transport Analysis in the Energy System: A Case Study for Germany through 2050. Energies *14*, 3166. https://doi.org/10.3390/en14113166.

138. Demir, M.E., and Dincer, I. (2018). Cost assessment and evaluation of various hydrogen delivery scenarios. Int. J. Hydrogen Energy *43*, 10420–10430. https://doi.org/10.1016/j.ijhydene.2017.08.002.

139. IRENA (2022). Global Hydrogen Trade to Meet the 1.5°C Climate Goal: Technology Review of Hydrogen Carriers. Glob. Hydrog. Trade to Meet 1.5°C Clim. Goal Technol. Rev. Hydrog. Carriers. https://www.irena.org/publications/2022/Apr/Global-hydrogen-trade-Part-II.

140. Gerboni, R. (2016). Introduction to hydrogen transportation. Compend. Hydrog. Energy, 283–299. https://doi.org/10.1016/B978-1-78242-362-1.00011-0.

141. Service, C.R. (2021). Pipeline Transportation of Hydrogen: Regulation, Research, and Policy. Congr. Res. Serv. (CRS). CRS Report, 46700, 1–29. https://crsreports.congress.gov/product/pdf/R/R46700.

142. Wulf, C., Reuß, M., Grube, T., Zapp, P., Robinius, M., Hake, J.-F., and Stolten, D. (2018). Life Cycle Assessment of hydrogen transport and distribution options. J. Clean. Prod. *199*, 431–443. https://doi.org/10.1016/j.jclepro.2018.07.180.

143. Enagás, Energinet, Fluxys Belgium, Gasunie, GRTgaz, NET4GAS, OGE, ONTRAS, Snam, Swedegas, et al. (2020). European Hydrogen Backbone. https://gasforclimate2050.eu/wp-content/uploads/2020/07/2020_European-Hydrogen-Backbone_Report.pdf/.

144. Jin, T., Tian, H., Gao, X., Liu, Y., Wang, J., Chen, H., and Lan, Y. (2017). Simulation and performance analysis of the perforated plate flowmeter for liquid hydrogen. Int. J. Hydrogen Energy *42*, 3890–3898. https://doi.org/10.1016/j.ijhydene.2016.09.072.

145. Lundblad, T., Taljegard, M., and Johnsson, F. (2023). Centralized and decentralized electrolysis-based hydrogen supply systems for road transportation – A modeling study of current and future costs. Int. J. Hydrogen Energy *48*, 4830–4844. https://doi.org/10.1016/j.ijhydene.2022.10.242.

146. Aasadnia, M., and Mehrpooya, M. (2018). Large-scale liquid hydrogen production methods and approaches: A review. Appl. Energy *212*, 57–83. https://doi.org/10.1016/j.apenergy.2017.12.033.

147. BNEF (2020). Hydrogen Economy Outlook. Key messages. Bloom. New Energy Financ., 12.

148. Vendt, M., and Wallmark, C. (2022). Prestudy H2 ESIN: Hydrogen, energy system and infrastructure in Northern Scandinavia and Finland. https://www.diva-portal.org/smash/record.jsf?pid=diva2%3A1719894&dswid=1954.

149. Ligen, Y., Vrubel, H., and Girault, H. (2020). Energy efficient hydrogen drying and purification for fuel cell vehicles. Int. J. Hydrogen Energy *45*, 10639–10647. https://doi.org/10.1016/j.ijhydene.2020.02.035.

150. Sunden, B. (2019). Fuel cell systems and applications. Hydrog. Batter. fuel cells, 203–216. https://doi.org/https://doi.org/10.1016/B978-0-12-816950-6.00011-7.

151. Hirscher, M. (2010). Handbook of Hydrogen Storage. https://doi.org/10.1002/9783527629800.

152. Allevi, C., and Collodi, G. (2017). Hydrogen production in IGCC systems. Integr. Gasif. Comb. Cycle Technol., 419–443. https://doi.org/10.1016/B978-0-08-100167-7.00012-3.

153. Di Profio, P., Arca, S., Rossi, F., and Filipponi, M. (2009). Comparison of hydrogen hydrates with existing hydrogen storage technologies: Energetic and economic evaluations. Int. J. Hydrogen Energy *34*, 9173–9180. https://doi.org/10.1016/j.ijhydene.2009.09.056.

154. Idealhy.eu (2014). Liquid Hydrogen Outline. https://www.idealhy.eu/index.php?page=lh2_outline.

155. Reuß, M., Grube, T., Robinius, M., Preuster, P., Wasserscheid, P., and Stolten, D. (2017). Seasonal storage and alternative carriers: A flexible hydrogen supply chain model. Appl. Energy *200*, 290–302. https://doi.org/10.1016/j.apenergy.2017.05.050.

156. Cardella, U., Decker, L., Sundberg, J., and Klein, H. (2017). Process optimization for large-scale hydrogen liquefaction. Int. J. Hydrogen Energy *42*, 12339–12354. https://doi.org/10.1016/j.ijhydene.2017.03.167.

157. Berstad, D., Skaugen, G., and Wilhelmsen, Ø. (2019). Concepts for efficient hydrogen liquefaction. 11–12.

158. Holladay, J., Meinhardt, K., Polikarpov, E., Thomsen, E., Barclay, J., Cui, J., Anderson, I., and Jensen, B. (2022). MagnetoCaloric Hydrogen Liquefaction. DOE Annu. Merit Rev. PD131, 35.

159. Office of Energy Efficiency & Renewable Energy (2017). Hydrogen Pipelines. https://www.energy.gov/eere/fuelcells/hydrogen-pipelines.

160. IRENA (2022). Global hydrogen trade. https://www.irena.org/Energy-Transition/Technology/Hydrogen/Global-hydrogen-trade.

161. European commission (2020). Hydrogen strategy for a climate neutral Europe. https://doi.org/10.18668/NG.2020.12.09.

162. Siemens energy (2020). Hydrogen infrastructure - the pillar of energy transition. Munich Siemens Energy, Gascade Gastransport GmbH, Nowega GmbH, 32. https://www.nowega.de/wp-content/uploads/200915-whitepaper-h2-infrastructure-EN.pdf.

163. European Commission (2022). Assessment of Hydrogen Delivery Options. Feasibility of Transport of Green Hydrogen within Europe. Eur. Com., 136. https://doi.org/10.2760/869085.

164. Wetegrove, M., Duarte, M.J., Taube, K., Rohloff, M., Gopalan, H., Scheu, C., Dehm, G., and Kruth, A. (2023). Preventing Hydrogen Embrittlement: The Role of Barrier Coatings for the Hydrogen Economy. Hydrog. *4*, 307–322. https://doi.org/10.3390/hydrogen4020022.

165. Gasunie (2018). Gasunie hydrogen pipeline from Dow to Yara brought into operation. https://www.gasunie.nl/en/news/gasunie-hydrogen-pipeline-from-dow-to-yara-brought-into-operation.

166. Collins, L. (2022). Why the Netherlands' planned hydrogen network will be difficult to replicate in other countries | Recharge. https://www.rechargenews.com/energy-transition/why-the-netherlands-planned-hydrogen-network-will-be-difficult-to-replicate-in-other-countries/2-1-1253792.

167. Energimarknadsinspektionen (2021). The Swedish natural gas system structure. https://www.ei.se/ei-in-english/natural-gas/the-swedish-natural-gas-system-structure.

168. Biec International Inc (2022). GalvaLume. https://www.galvalume.com/.

169. Brasil (2024). Anuário Estatístico Brasileiro do Petróleo, Gás Natural e Biocombustíveis 2024. https://www.gov.br/anp/pt-br/centrais-de-conteudo/publicacoes/anuario-estatistico/anuario-estatistico-brasileiro-do-petroleo-gas-natural-e-biocombustiveis-2024#Secao4.

170. Kanchiralla, F.M., Brynolf, S., and Mjelde, A. (2024). Role of biofuels, electro-fuels, and blue fuels for shipping: environmental and economic life cycle considerations. Energy Environ. Sci. *17*, 6393–6418. https://doi.org/10.1039/D4EE01641F.

171. Office of Energy Efficiency & Renewable Energy (2017). Hydrogen Storage. https://www.energy.gov/eere/fuelcells/hydrogen-storage.

172. Kawasaki (2015). From LNG Carriers to Liquefied Hydrogen Carriers. https://answers.khi.co.jp/en/energy-environment/20150430e-01/.

173. Kawasaki (2023). Technological Development of Cargo Tank for Large Liquefied Hydrogen Carriers Completed. https://global.kawasaki.com/en/corp/newsroom/news/detail/?f=20230606_4159.

174. Kawasaki (2022). World's First AiP Granted to Kawasaki's 2.4 MW Class Dual Fuel Generator Engine Using Hydrogen Gas as Fuel. https://global.kawasaki.com/en/corp/newsroom/news/detail/?f=20221130_6926.

175. Element Energy (2018). Hydrogen supply chain evidence base. https://assets.publishing.service.gov.uk/government/uploads/system/uploads/attachment_data/file/760479/H2_supply_chain_evidence_-_publication_version.pdf.

176. Masoudi, M., Hassanpouryouzband, A., Hellevang, H., and Haszeldine, R.S. (2024). Lined rock caverns: A hydrogen storage solution. J. Energy Storage *84*, 110927. https://doi.org/10.1016/j.est.2024.110927.

177. Crotogino, F., Schneider, G.-S., and Evans, D.J. (2018). Renewable energy storage in geological formations. Proc. Inst. Mech. Eng. Part A J. Power Energy *232*, 100–114. https://doi.org/10.1177/0957650917731181.

178. Muhammed, N.S., Haq, B., Al Shehri, D., Al-Ahmed, A., Rahman, M.M., and Zaman, E. (2022). A review on underground hydrogen storage: Insight into geological sites, influencing factors and future outlook. Energy Reports *8*, 461–499. https://doi.org/10.1016/j.egyr.2021.12.002.

179. Hyunder (2013). Why storing large scale intermittent renewable energies with hydrogen? https://hyunder.eu/.

180. Abreu, J.F., Costa, A.M., Costa, P.V.M., Miranda, A.C.O., Zheng, Z., Wang, P., Goulart, M.B.R., Bergsten, A., Ebecken, N.F.F., Bittencourt, C.H., et al. (2023). Large-scale storage of hydrogen in salt caverns for carbon footprint reduction. Int. J. Hydrogen Energy *48*, 14348–14362. https://doi.org/10.1016/j.ijhydene.2022.12.272.

181. Małachowska, A., Łukasik, N., Mioduska, J., and Gębicki, J. (2022). Hydrogen Storage in Geological Formations—The Potential of Salt Caverns. Energies *15*, 5038. https://doi.org/10.3390/en15145038.

182. Marnate, K., and Grönkvist, S. (2024). Looking beyond compressed hydrogen storage for Sweden: Opportunities and barriers for chemical hydrides. Int. J. Hydrogen Energy *77*, 677–694. https://doi.org/10.1016/j.ijhydene.2024.06.106.

183. Hybrit (2022). HYBRIT: A unique, underground, fossil-free hydrogen gas storage facility is being inaugurated in Luleå. https://www.hybritdevelopment.se/en/hybrit-a-unique-underground-fossil-free-hydrogen-gas-storage-facility-is-being-inaugurated-in-lulea/.

184. Johansson, F., Spross, J., Damasceno, D., Johansson, J., and Stille, H. (2018). Investigation of research needs regarding the storage of hydrogen gas in lined rock caverns. Tech. report, 2018 KTH R. Inst. Technol. https://www.diva-portal.org/smash/get/diva2:1221714/FULLTEXT01.pdf.

185. Hystories (2023). Report on the environmental impact of the underground H2 storage. 1–48. https://hystories.eu/wp-content/uploads/2023/04/Hystories_D6.3-Results-for-E-LCA.pdf.

186. Derking, H., Togt, L. van der, and Keezer, M. (2019). Liquid Hydrogen Storage: Status and Future Persectives. Cryoworld Adv. Cryog., 18.

187. Linde (2020). Liquid Hydrogen Distribution Technology - HYPER closing seminar. Hyper. Closing Semin., 27. https://www.sintef.no/globalassets/project/hyper/presentations-day-2/day2_1105_decker_liquid-hydrogen-distribution-technology_linde.pdf.

188. Entegris (2023). MegaTorr® PS7-A Hydrogen Gas Purifiers. https://www.entegris.com/shop/en/USD/products/gas-filtration-and-purification/gas-purifiers/MegaTorr-PS7-A-Hydrogen-Gas-Purifiers/p/GateKeeperMGTAutoRegenerableHydrogenGasPurifiers.

189. Ivysads (2023). Hydrogen Purification Solutions. https://ivysads.com/hydrogen/.

190. Plötz, P. (2022). Hydrogen technology is unlikely to play a major role in sustainable road transport. Nat. Electron. *5*, 8–10. https://doi.org/10.1038/s41928-021-00706-6.

191. Vätgas Sverige (2022). Major expansion of hydrogen filling stations. https://vatgas.se/en/fakta/refuel-with-hydrogen/.

192. Andersson, L., and Jansson, J. (2024). Personal communication with Volvo experts.

193. Marcinkoski, J., Vijayagopal, R., Adams, J., James, B., Kopasz, J., and Ahluwalia, R. (2019). DOE Advanced Truck Technologies - Subsection of the Electrified Powertrain Roadmap. 1–31. https://www.hydrogen.energy.gov/docs/hydrogenprogramlibraries/pdfs/19006_hydrogen_class8_long_haul_truck_targets.pdf?Status=Master.

194. Volvotrucks (2020). Hydrogen fuel cells: All your questions answered. https://www.volvotrucks.com/en-en/news-stories/insights/articles/2020/jun/hydrogen-fuel-cells-all-your-questions-answered.html.

195. ICCT (2023). The european heavy-duty vehicle market until 2040: analysis of decarbonization pathways. Int. Counc. Clean Transp., 1–58. https://theicct.org/wp-content/uploads/2023/01/hdv-europe-decarb-costs-jan23.pdf.

196. Daimler (2023). Fuel-Cell Technology: Daimler Truck Builds First Mercedes-Benz GenH2 Truck Customer-Trial Fleet. https://www.daimlertruck.com/en/newsroom/pressrelease/fuel-cell-technology-daimler-truck-builds-first-mercedes-benz-genh2-truck-customer-trial-fleet-52552943.

197. Man (2024). MAN expands its zero-emission portfolio. https://press.mantruckandbus.com/corporate/man-expands-its-zero-emission-portfolio/.

198. Löfving, J., Brynolf, S., and Grahn, M. (2025). Geospatial distribution of hydrogen demand and refueling infrastructure for long-haul trucks in Europe. Int. J. Hydrogen Energy *128*, 544–558.

https://doi.org/10.1016/j.ijhydene.2025.04.257.

199. Zhang, C., Kotz, A., Kelly, K., and Rippelmeyer, L. (2021). Development of heavy-duty vehicle representative driving cycles via decision tree regression. Transp. Res. Part D Transp. Environ. *95*, 102843. https://doi.org/10.1016/j.trd.2021.102843.

200. Wei, H., Hu, Z., Ma, J., Ma, W., Yuan, S., Hu, Y., Hu, K., Zhou, L., and Wei, H. (2023). Experimental study of thermal efficiency and NOx emission of turbocharged direct injection hydrogen engine based on a high injection pressure. Int. J. Hydrogen Energy *48*, 12905–12916. https://doi.org/10.1016/j.ijhydene.2022.12.031.

201. Ferrara, A., Jakubek, S., and Hametner, C. (2021). Energy management of heavy-duty fuel cell vehicles in real-world driving scenarios: Robust design of strategies to maximize the hydrogen economy and system lifetime. Energy Convers. Manag. *232*, 113795. https://doi.org/10.1016/j.enconman.2020.113795.

202. ICCT (2024). Euro 7: The new emission standard for light-and heavy-duty vehicles in the European Union. ICCT Policy Updat. *1*, 1–9. https://data.consilium.europa.eu/doc/document/ST-16960-2023-REV-1/.

203. Council of the European Union (2023). Regulation on type-approval of motor vehicles and engines and of systems, components and separate technical units intended for such vehicles, with respect to their emissions and battery durability (Euro 7). *2023*, 1–99. https://data.consilium.europa.eu/doc/document/ST-16960-2023-REV-1/en/pdf.

204. Polukarova, M., Hjort, M., and Gustafsson, M. (2024). Comprehensive approach to national tire wear emissions: Challenges and implications. Sci. Total Environ. *924*. https://doi.org/10.1016/j.scitotenv.2024.171391.

205. Geilenkirchen, J. (2018). Methods for calculating the emissions of transport in the. 67. https://www.pbl.nl/uploads/default/downloads/pbl-2020-methods-for-calculating-the-emissions-of-transport-in-the-netherlands-2020-4139.pdf.

206. Ten Broeke, H., Hulskotte, J., and Denier van der Gon, H. (2008). Road traffic tyre wear. Emission estimates for diffuse sources Netherlands Emission Inventory.

207. Scania (2021). Battery electric vs diesel driven: LCA of distribution vehicles. Scania. https://www.scania.com/group/en/home/newsroom/press-releases/press-release-detail-page.html/3999115-scania-publishes-life-cycle-assessment-of-battery-electric-vehicles.

208. Arrigoni, A., and Bravo, L. (2022). Hydrogen Emissions from a Hydrogen Economy and their Potential Global Warming Impact. Inst. gas Eng. Manag. https://www.h2knowledgecentre.com/content/researchpaper3964.

209. Cooper, J., Dubey, L., Bakkaloglu, S., and Hawkes, A. (2022). Hydrogen emissions from the hydrogen value chain-emissions profile and impact to global warming. Sci. Total Environ. *830*, 154624. https://doi.org/10.1016/j.scitotenv.2022.154624.

210. Esquivel-Elizondo, S., Hormaza Mejia, A., Sun, T., Shrestha, E., Hamburg, S.P., and Ocko, I.B. (2023). Wide range in estimates of hydrogen emissions from infrastructure. Front. Energy Res. *11*, 1–8. https://doi.org/10.3389/fenrg.2023.1207208.

211. Fan, Z., Sheerazi, H., Bhardwaj, A., Corbeau, A.-S., Longobardi, K., Castañeda, A., Merz, A.-K., Caleb, D.R., Woodall, M., Agrawal, M., et al. (2022). Hydrogen leakage: a potential risk for the hydrogen economy. Cent. Glob. Energy Policy (CGEP), Columbia Univ., 1–33.

212. Abe, J.O., Popoola, A.P.I., Ajenifuja, E., and Popoola, O.M. (2019). Hydrogen energy, economy and storage: Review and recommendation. Int. J. Hydrogen Energy *44*, 15072–15086. https://doi.org/10.1016/j.ijhydene.2019.04.068.

213. Van Ruijven, B., Lamarque, J.F., Van Vuuren, D.P., Kram, T., and Eerens, H. (2011). Emission scenarios for a global hydrogen economy and the consequences for global air pollution. Glob. Environ. Chang. *21*, 983–994. https://doi.org/10.1016/j.gloenvcha.2011.03.013.

214. Rechberger, K., Spanlang, A., Sasiain Conde, A., Wolfmeir, H., and Harris, C. (2020). Green Hydrogen-Based Direct Reduction for Low-Carbon Steelmaking. steel Res. Int. *91*, 1–10. https://doi.org/10.1002/srin.202000110.

215. Nurdiawati, A., Zaini, I.N., Wei, W., Gyllenram, R., Yang, W., and Samuelsson, P. (2023). Towards fossil-free steel: Life cycle assessment of biosyngas-based direct reduced iron (DRI) production process. J. Clean. Prod. *393*, 136262. https://doi.org/10.1016/j.jclepro.2023.136262.

216. Jernkontoret (2023). Facts and key ratios. https://www.jernkontoret.se/en/the-steel-industry/industry-facts-and-statistics/facts-and-key-ratios/.

217. Nduagu, E.I., Yadav, D., Bhardwaj, N., Elango, S., Biswas, T., Banerjee, R., and Rajagopalan, S. (2022). Comparative life cycle assessment of natural gas and coal-based directly reduced iron (DRI) production: A case study for India. J. Clean. Prod. *347*, 131196. https://doi.org/10.1016/j.jclepro.2022.131196.

218. Yilmaz, C., and Turek, T. (2017). Modeling and simulation of the use of direct reduced iron in a blast furnace to reduce carbon dioxide emissions. J. Clean. Prod. *164*, 1519–1530. https://doi.org/10.1016/j.jclepro.2017.07.043.

219. Echterhof, T. (2021). Review on the Use of Alternative Carbon Sources in EAF Steelmaking. Metals (Basel). *11*, 222. https://doi.org/10.3390/met11020222.

220. Gielen, D., Saygin, D., Taibi, E., and Birat, J. (2020). Renewables-based decarbonization and relocation of iron and steel making: A case study. J. Ind. Ecol. *24*, 1113–1125. https://doi.org/10.1111/jiec.12997.

221. SSAB (2024). HYBRIT: Six years of research paves the way for fossil-free iron and steel production on an industrial scale. https://www.ssab.com/en/news/2024/08/hybrit-six-years-of-research-paves-the-way-for-fossilfree-iron-and-steel-production-on-an-industrial.

222. Vattenfall (2022). Industry decarbonisation. https://group.vattenfall.com/what-we-do/roadmap-to-fossil-freedom/industry-decarbonisation/hybrit.

223. Meng, F., Olivetti, E.A., Zhao, Y., Chang, J.C., Pickering, S.J., and McKechnie, J. (2018). Comparing Life Cycle Energy and Global Warming Potential of Carbon Fiber Composite Recycling Technologies and Waste Management Options. ACS Sustain. Chem. Eng. *6*, 9854–9865. https://doi.org/10.1021/acssuschemeng.8b01026.

224. Zhang, J., Chevali, V.S., Wang, H., and Wang, C.-H. (2020). Current status of carbon fibre and carbon fibre composites recycling. Compos. Part B Eng. *193*, 108053. https://doi.org/10.1016/j.compositesb.2020.108053.

225. Zhu, J.-H., Chen, P., Su, M., Pei, C., and Xing, F. (2019). Recycling of carbon fibre reinforced plastics by electrically driven heterogeneous catalytic degradation of epoxy resin. Green Chem. *21*, 1635–1647. https://doi.org/10.1039/C8GC03672A.

226. Ren, Y., Xu, L., Shang, X., Shen, Z., Fu, R., Li, W., and Guo, L. (2022). Evaluation of Mechanical Properties and Pyrolysis Products of Carbon Fibers Recycled by Microwave Pyrolysis. ACS Omega *7*, 13529–13537. https://doi.org/10.1021/acsomega.1c06652.

227. Ngo, P., and Mohan, A. (2024). Design of type IV compressed gas hydrogen tanks made of carbon fiber for sustainability and circularity.

228. Pospíšil, J., Charvát, P., Arsenyeva, O., Klimeš, L., Špiláček, M., and Klemeš, J.J. (2019). Energy demand of liquefaction and regasification of natural gas and the potential of LNG for operative thermal energy storage. Renew. Sustain. Energy Rev. *99*, 1–15. https://doi.org/10.1016/j.rser.2018.09.027.

229. Bright-Renewables (2023). Biomethane liquefaction (bio-LNG): LiquiPac. https://www.bright-renewables.com/solutions/renewable-gas/biomethane-liquefaction/.

230. Transport & Environment (2021). LNG trucks: a dead end bridge. https://www.transportenvironment.org/discover/lng-trucks-a- dead-end-bridge/.
